# Spatial Architecture of Myeloid and T Cells Orchestrates Immune Evasion and Clinical Outcome in Lung Cancer

**DOI:** 10.1158/2159-8290.CD-23-1380

**Published:** 2024-04-12

**Authors:** Katey S.S. Enfield, Emma Colliver, Claudia Lee, Alastair Magness, David A. Moore, Monica Sivakumar, Kristiana Grigoriadis, Oriol Pich, Takahiro Karasaki, Philip S. Hobson, Dina Levi, Selvaraju Veeriah, Clare Puttick, Emma L. Nye, Mary Green, Krijn K. Dijkstra, Masako Shimato, Ayse U. Akarca, Teresa Marafioti, Roberto Salgado, Allan Hackshaw, TRACERx consortium, Mariam Jamal-Hanjani, Febe van Maldegem, Nicholas McGranahan, Benjamin Glass, Hanna Pulaski, Eric Walk, James L. Reading, Sergio A. Quezada, Crispin T. Hiley, Julian Downward, Erik Sahai, Charles Swanton, Mihaela Angelova

**Affiliations:** 1 Cancer Evolution and Genome Instability Laboratory, The Francis Crick Institute, London, United Kingdom.; 2 Cancer Research UK Lung Cancer Centre of Excellence, University College London Cancer Institute, London, United Kingdom.; 3 Department of Cellular Pathology, University College London Hospitals, London, United Kingdom.; 4 Cancer Genome Evolution Research Group, Cancer Research UK Lung Cancer Centre of Excellence, University College London Cancer Institute, London, United Kingdom.; 5 Cancer Metastasis Laboratory, University College London Cancer Institute, London, United Kingdom.; 6 Flow Cytometry, The Francis Crick Institute, London, United Kingdom.; 7 Experimental Histopathology, The Francis Crick Institute, London, United Kingdom.; 8 Department of Pathology, ZAS Hospitals, Antwerp, Belgium.; 9 Division of Research, Peter MacCallum Cancer Centre, Melbourne, Australia.; 10 Cancer Research UK and University College London Cancer Trials Centre, London, United Kingdom.; 11 Department of Oncology, University College London Hospitals, London, United Kingdom.; 12 Oncogene Biology Laboratory, The Francis Crick Institute, London, United Kingdom.; 13 PathAI, Inc., Boston, Massachusetts.; 14 Pre-cancer Immunology Laboratory, University College London Cancer Institute, London, United Kingdom.; 15 Immune Regulation and Tumour Immunotherapy Group, Cancer Immunology Unit, Research Department of Haematology, University College London Cancer Institute, London, United Kingdom.; 16 Tumour Cell Biology Laboratory, The Francis Crick Institute, London, United Kingdom.

## Abstract

Understanding the role of the tumor microenvironment (TME) in lung cancer is critical to improving patient outcomes. We identified four histology-independent archetype TMEs in treatment-naïve early-stage lung cancer using imaging mass cytometry in the TRACERx study (*n* = 81 patients/198 samples/2.3 million cells). In immune-hot adenocarcinomas, spatial niches of T cells and macrophages increased with clonal neoantigen burden, whereas such an increase was observed for niches of plasma and B cells in immune-excluded squamous cell carcinomas (LUSC). Immune-low TMEs were associated with fibroblast barriers to immune infiltration. The fourth archetype, characterized by sparse lymphocytes and high tumor-associated neutrophil (TAN) infiltration, had tumor cells spatially separated from vasculature and exhibited low spatial intratumor heterogeneity. TAN-high LUSC had frequent *PIK3CA* mutations. TAN-high tumors harbored recently expanded and metastasis-seeding subclones and had a shorter disease-free survival independent of stage. These findings delineate genomic, immune, and physical barriers to immune surveillance and implicate neutrophil-rich TMEs in metastasis.

**Significance:**

This study provides novel insights into the spatial organization of the lung cancer TME in the context of tumor immunogenicity, tumor heterogeneity, and cancer evolution. Pairing the tumor evolutionary history with the spatially resolved TME suggests mechanistic hypotheses for tumor progression and metastasis with implications for patient outcome and treatment.

*This article is featured in Selected Articles from This Issue, p. 897*

## Teaser

Distinct microenvironment architectures and spatial cellular niches were observed in earlystage non–small cell lung cancer and were linked to tumor immunogenicity, metabolism, and clonal evolution, implicating neutrophils in poor patient outcomes.

## INTRODUCTION

The tumor microenvironment (TME) confers a selective pressure on the clonal evolution of lung tumors. Whether the TME promotes or suppresses tumor growth is linked to its spatial organization and cell phenotypes. Recently, highly multiplexed technologies such as imaging mass cytometry (IMC) have unveiled the complexities in the composition and structure of the TME across several cancer types (1–9). These studies have demonstrated the clinical relevance of in-depth spatial approaches, identifying multicellular organizations associated with patient outcomes, tumor phenotypes, and therapy response in lung adenocarcinoma (LUAD), breast cancer, and glioma.

TME spatial organization can, in turn, be modulated by somatic alterations incurred throughout tumor evolution. IMC studies in breast cancer have identified distinct spatial TME structures associated with somatic driver mutations and histology-specific outcomes (2, 4), demonstrating the value of such integrative analyses. We previously showed that increased immune infiltration, inferred from transcriptomic signatures, was associated with frequent cancer cell–intrinsic immune-escape mechanisms that impact neoantigen presentation, including loss of heterozygosity (LOH) of human leukocyte antigen (HLA) alleles, in non–small cell lung cancer (NSCLC; refs. 10, 11). These results suggested the selection of immune evasive tumor cell populations in a predatory microenvironment. However, spatially resolved information is required to further understand immune pressures through, for example, cell-to-cell interactions and physical barriers to immune surveillance that impact tumor evolution. NSCLC studies that pair spatial detail from high-dimensional imaging with genomics and transcriptomics in clinically well-defined cohorts representing the major histologic subtypes remain to be undertaken. Such studies are needed to provide mechanistic insights into immune escape and the TME pressures on cancer evolution.

Here, we used multiregion IMC to comprehensively characterize TME composition and spatial organization in 198 tumor and normal regions from 81 treatment-naïve patients with NSCLC in the TRACERx 100 cohort (12). TRACERx [TRAcking Cancer Evolution through therapy (Rx); ClinicalTrials.gov: NCT01888601] is a prospective study of tumor evolution through multiregion tumor sampling in patients with early-stage resectable disease. Using paired IMC, pathology, whole-exome sequencing (WES), and RNA-sequencing data (13–15), we studied the link between TME organization, tumor immunogenicity, and evolutionary history. We investigated how the TME may be shaped by high neoantigen burden, intrinsic immune-escape mechanisms, and evolutionary patterns associated with poor outcomes. This work begins to unravel the complex TME relationships with NSCLC tumor evolution. By examining TME spatial heterogeneity, this study furthers the current knowledge of a critical open question—how to address TME heterogeneity and utilize the TME context for clinical applications.

## RESULTS

### Building an Atlas of the Early-Stage Non–Small Cell Lung Cancer Microenvironment

**Figure 1.**
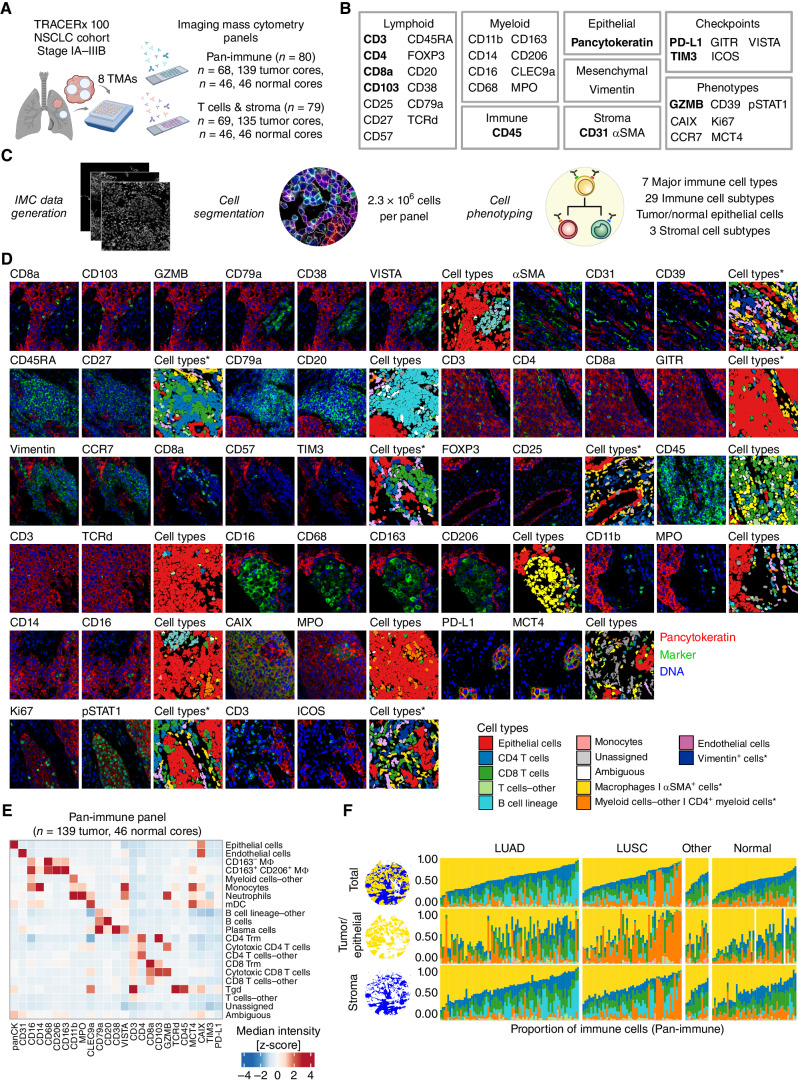
IMC workflow defines the single-cell spatial landscape of the NSCLC tumor microenvironment. **A,** TRACERx 100 IMC cohort. We developed and applied two IMC antibody panels, Pan-immune and T cells and stroma, to tissue microarrays (TMA) from clinical samples collected at surgical resection (created with BioRender.com). **B,** Targets of antibodies described in this study. Bold text indicates targets detected in both IMC panels. **C,** IMC data were acquired from stained TMAs and processed to identify single cells and their phenotypes. **D,** 40,000 μm^2^ crops of IMC images representing the markers from **B** with corresponding cell types from the pan-immune panel, unless annotated with an asterisk for the T cells and stroma panel only. **E,** A heat map of the z-score normalized median intensities of markers from the pan-immune panel across the identified cell subtypes. **F,** Proportion of major immune cell types identified in the pan-immune IMC data set per TMA core, calculated over the total tissue area (illustrated as blue and gold domains), tumor/epithelial compartment (gold domain), or the stromal compartment (blue domain). In two normal cores, the epithelial cell signal reflected very thin cells, which were not resolved into an epithelial compartment. All data from these cores are represented by the stroma compartment. Cell types color legend applies to **D** and **F**, where asterisks denote cell types identified in T cells and stroma panel only. LUAD, lung adenocarcinoma; LUSC, lung squamous cell carcinoma; NSCLC, non–small cell lung cancer; other, other non–small cell lung cancer histologies; IMC, imaging mass cytometry.

With the aim of understanding the role of the TME in tumor evolution, we performed an in-depth spatial and phenotypic analysis of the TME in early-stage (I–IIIA), treatment-naïve NSCLC. We characterized the diversity in cell phenotypes, recurrent spatial communities, and broader TME classes in the TRACERx 100 multiregion cohort (12). Using IMC, we profiled the *in situ* expression of 38 markers on tissue microarrays (TMA) of spatially separated tumor and adjacent normal lung samples acquired at surgical resection (Fig. 1A). The TME organization was analyzed using the pan-immune antibody panel targeting innate and adaptive immune cell types (*n* = 80, 185 cores; Fig. 1B; Supplementary Table S1). Additional T-cell differentiation states and nonimmune stromal cells were interrogated using the T cells and stroma panel (*n* = 79, 181 cores). The major histologic subtypes of NSCLC were represented in the cohort, including LUAD (*n* = 39 tumors, 76 cores, pan-immune panel), lung squamous cell carcinoma (LUSC; *n* = 23, 50 cores), and other NSCLC histologies (NSCLC-Other; *n* = 6, 13 cores) as well as adjacent normal lung samples (*n* = 46, 46 cores). IMC data from multiple tumor cores were available for 41 of the tumors represented. Twelve patients only had IMC data available from adjacent normal lung cores. The majority of cores were profiled with both IMC panels (168/198) and had paired WES and RNA sequencing (RNA-seq) data available (Supplementary Fig. S1A–S1C; Supplementary Table S2; Methods).

To comprehensively characterize the cell phenotypes *in situ*, deep learning–guided cell segmentation was performed and followed by single-cell phenotyping (Fig. 1C; Supplementary Fig. S2A–S2D). We identified 2.3 million cells per antibody panel from seven major immune cell types and 29 immune cell subtypes, in addition to epithelial, endothelial, and αSMA^+^ cells (Fig. 1C–E; Supplementary Fig. S2B; Supplementary Fig. S3A). Additional pathologist-guided labels were created using paired IMC and hematoxylin and eosin (H&E)–stained images to distinguish features unresolvable by marker expression alone. These pathologist labels further distinguished αSMA^+^ perivascular stroma and αSMA^+^ fibroblasts, alveolar macrophages, and tumor and nontumor epithelial cells, which were used to interrogate tumor cell–specific phenotypes and spatial metrics (Fig. 1C; Supplementary Fig. S2C and S2D). Cell phenotypes were quantified on the basis of marker positivity, examining, for example, hypoxia (CAIX), lactate metabolism (MCT4), proliferation (Ki-67), the exhausted terminally differentiated dysfunctional (TDT) T-cell state (CD39, CD57; ref. 16), and immune-checkpoint molecules (Fig. 1B and D; Methods).

Macrophages were the most prevalent major immune cell type in NSCLC cores (median 40% of immune cells, tumor cores; Fig. 1F; Supplementary Fig. S3B), in line with other studies (1), with CD163^+^CD206^+^ macrophages comprising a greater proportion than CD163^−^ macrophages (37% vs. 2%; Supplementary Fig. S3C). Notably, B cells in LUAD and myeloid cells-other, predominantly comprising neutrophils, in LUSC made up >25% of immune cells in a subset of tumor cores (14/76 LUAD, 13/50 LUSC; Fig. 1F; Supplementary Fig. S3C). From the T cells and stroma panel, αSMA^+^ cells were the most abundant nonepithelial cell populations in tumor cores (median 16% of all cells; Supplementary Fig. S3D). Subtypes of CD4 and CD8 T cells were categorized as regulatory T cells (Treg), naïve, cytotoxic, memory, and exhausted populations (Supplementary Fig. S3E). Endothelial cells made up a greater proportion of total cells in adjacent normal cores than tumor cores (22% vs. 7%), in accordance with the physiologic function of the lung in gas transfer through blood flow (Supplementary Fig. S3B and S3D).

To investigate the spatial context of the identified cell phenotypes, we quantified cell densities within two tissue compartments, tumor nest/epithelium, and stroma (Fig. 1F; Supplementary Fig. S2A). Additionally, we performed analysis of local cellular neighborhoods in NSCLC, which have recently been shown to correlate with clinical outcomes in LUAD (1), and revealed 10 recurrent geographical communities (*C0*–*C9*) of frequently colocalizing cells within tumor cores across histologic subtypes (Supplementary Fig. S3F and S3G; Methods).

We assessed the relationship of cell populations and communities with clinical variables (Supplementary Fig. S4A–S4C; Methods). In both LUAD and LUSC, densities of the community *C9:B cells and plasma cells* and plasma cells, when tested separately, were associated with a high tumor mutational burden (TMB). High TMB was further associated with increased densities of CD163^−^ macrophages and CD4 Tem in LUAD and CD8-exhausted TDTs in LUSC, similar to previous observations (16). Among the significant associations, the *C6:macrophages and T cells* community, as well as several cell subtypes that characterize this community, were enriched in current smokers compared with ex- and never-smokers in LUAD.

Together, we integrated spatial and phenotypic information from multiplexed imaging with pathology annotations to develop a framework for studying the TME composition and organization in NSCLC.

### Immune Composition in Tumor Nests and Surrounding Stroma Reveals Four TME Classes in NSCLC

Three broad immune classes have been previously described for solid tumors through histologic examination of tumor-infiltrating lymphocytes (TIL) quantity and location within tumor sections: inflamed, immune-excluded, and cold (17). The TME subtypes and their spatial heterogeneity in NSCLC remain to be comprehensively characterized in association with genetic, molecular, and cellular mechanisms of immune escape.

**Figure 2.**
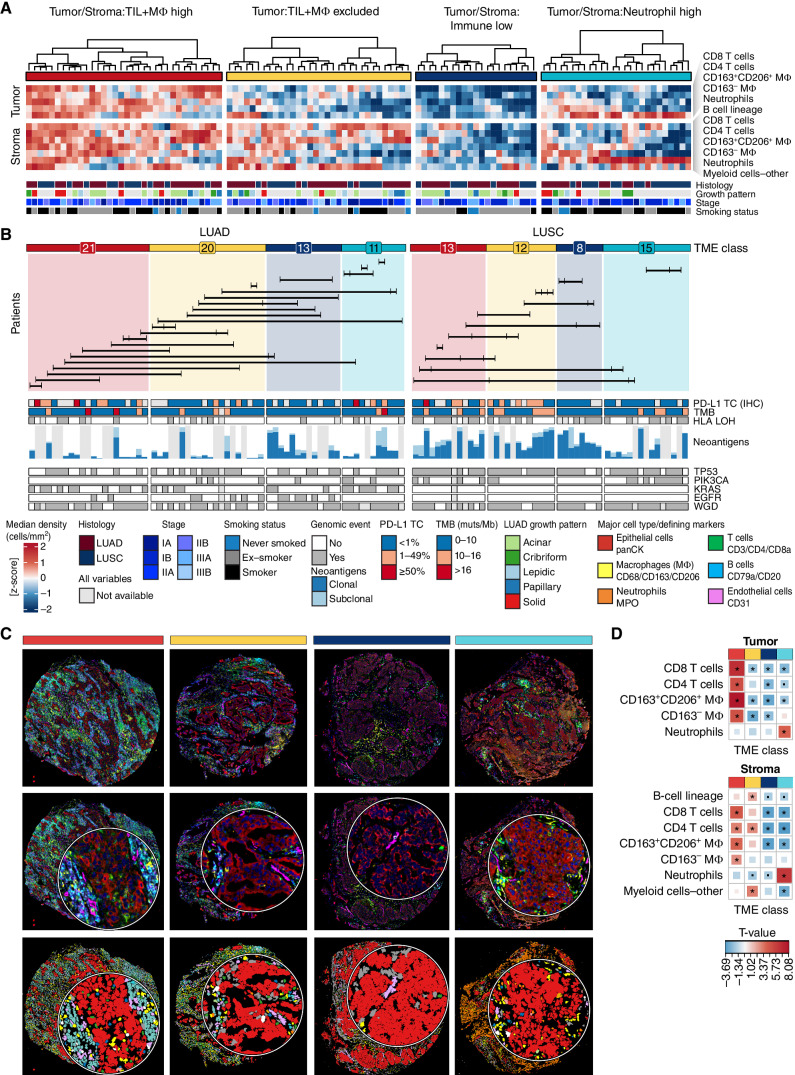
Four TME classes in NSCLC defined by immune composition in tumor nests and surrounding stroma. **A,** Tumor cores were classified into four TME classes, derived by clustering immune cell densities in the tumor nest and stroma. Only LUAD (*n* = 65 cores, 35 tumors) and LUSC (*n* = 48, 23 tumors) tumor cores are featured, and corresponding clinical annotations are displayed. Regional growth patterns are shown for LUAD: lepidic (low grade), acinar and papillary (mid-grade), solid and cribriform (high grade). **B,** TME classifications displayed separately for LUAD and LUSC. Numbers indicate the number of cores with a given TME class for each histology subtype. The barplot shows the total expressed neoantigen count for all predicted HLA alleles in the range 0–269 for LUAD and 23–160 for LUSC, colored by their clonal and subclonal status. Horizontal lines connect tumor cores from the same multiregion tumor (*n* = 33 tumors). The annotation bars display tumor genomic features and PD-L1 tumor cell (TC) staining (SP142 IHC) for the corresponding tumor cores. **C,** Composite images and cell type maps of representative examples for each TME class. Crop insets are 82 μm in diameter. **D,** A heat map of T values derived from an LMEM of the major cell type density across TME classes, adjusted for histology subtype as a fixed effect and patient as a random effect. Significant relationships are indicated with an asterisk for *P* ≤ 0.05. TIL, tumor-infiltrating lymphocyte; MΦ, macrophage; LUAD, lung adenocarcinoma; LUSC, lung squamous cell carcinoma; NSCLC, non–small cell lung cancer; TME, tumor microenvironment; TMB, tumor mutation burden; muts/Mb, mutations per megabase; panCK, pancytokeratin; LMEM, linear mixed effects model.

To understand how TME organization is associated with immune escape and tumor evolution, we performed a broad classification based on the densities of major immune cell types and identified common TME architectures in NSCLC. We quantified the densities of major cell types of adaptive and innate immunity defined within the tumor nest and stroma tissue compartments. Through unsupervised hierarchical clustering, we observed four common TME classes defined across all histologic subtypes in NSCLC (Fig. 2A–C; Supplementary Fig. S5A–S5C). These TME classes were distinguished by differential cell densities of three broad immune cell populations—TILs (T cells and B cells), macrophages (Mφ), and neutrophils—within the tumor nest (T) or stroma (S) of one TME class compared with other TME classes and annotated accordingly as *TS:TIL+MΦ high*, *T:TIL+MΦ excluded*, *TS:Immune low*, and *TS:Neutrophil high*. For a small proportion of tumor cores in the cohort (11.5%), the TME class was labeled *undefined* (Supplementary Fig. S5A; Methods).

The *TS:TIL+MΦ high* class, accounting for 28% of NSCLC tumor cores (*n* = 21 LUAD, 13 LUSC, 5 NSCLC-Other cores), consisted of immunologically “hot” tumors characterized by high infiltration of TILs and Mφs in both the tumor nest and stroma region (TS; Fig. 2D). Most of the identified TIL and Mφ subtypes, ranging from naïve CD8 T cells to CD8-exhausted TDTs, were enriched in this class compared with other TME classes (Supplementary Fig. S5D).

Across all histologic subtypes, we observed a subset of tumor cores with low infiltration of TILs and Mφs in the tumor nest and high infiltration of B cells, CD4 T cells, and a subset of myeloid cells excluding neutrophils and macrophages in the stroma (Fig. 2D). Because of the statistically lower infiltration of TILs and Mφs in the tumor nest (T), these cores were labeled *T:TIL+M*Φ* excluded* (24% of NSCLC cores, *n* = 20 LUAD, 12 LUSC, 1 NSCLC-other cores).

A smaller proportion of tumor cores (17%) had significantly lower TS infiltration of T cells and Mφs compared with those from other TME classes, termed *TS:Immune low* (*n* = 13 LUAD, 8 LUSC, 3 NSCLC-other cores).

Finally, we observed a distinct TME class, *TS:Neutrophil high*, in 19% of tumor cores (*n* = 11 LUAD, 15 LUSC, 1 NSCLC-Other cores) with lower infiltration of TILs and Mφ in both the tumor nest and stroma and significant enrichment of neutrophils in the tumor nest or the stroma (TS) compared with tumor cores from other TME classes. Although the TME classes were defined for all histologic subtypes combined, LUSC cores were enriched for neutrophils in the tumor nest more frequently than LUAD cores (Supplementary Fig. S5D).

To assess the intratumor heterogeneity (ITH) of these TME classes, we estimated the probability of observing the same TME class across all cores of a tumor (*n* = 41 tumors, bootstrap sampling of two to four cores per tumor, Methods). The *TS:Neutrophil high* TME had the highest probability to be detected in all cores (0.5), followed by *TS:Immune low* (0.38), *TS:TIL+M*Φ* high* (0.3), and *TS:TIL+M*Φ* excluded* (0.21).

Comparison by clinical variables showed that the *TS:Immune low* class was associated with low TMB and <1% PD-L1 immunohistochemistry (IHC) tumor score in LUAD and LUSC (Fig. 2B) and tumors from never and ex-smokers in LUAD (Supplementary Fig. S5E). IHC PD-L1 tumor cell positivity was also absent (<1%) in *TS:Neutrophil high* LUSCs, whereas a positivity score of ≥1% was significantly enriched with *TS:TIL+M*Φ* high* LUADs and *T:TIL+M*Φ* excluded* LUSCs compared with cores from other TME classes. In LUSC, the *TS:TIL+M*Φ* high* class was enriched in stage II and III tumors compared with stage I (Supplementary Fig. S5E). The tumor cores with a *TS:Neutrophil high* class in LUAD more frequently had a high-grade growth pattern compared with low and mid-grade patterns combined (50% vs. 10%, *P* = 0.048, Fig. 2A). These results align with previous reports of TIL associations with tumor cell PD-L1 expression and TMB (18).

### Multicellular Communities Associate with Neoantigen Burden and Intrinsic Immune Evasion

We sought to understand how the observed TME organization was related to neoantigen presentation and cancer cell–intrinsic mechanisms of immune evasion and identify potential tumor-extrinsic mediators of immune evasion. Using samples with paired IMC, WES, and RNA-seq data, we investigated the relationships of the spatially resolved cell subtypes, cellular communities, and TME classes with neoantigen burden and antigen presentation machinery (APM) defects.

**Figure 3.**
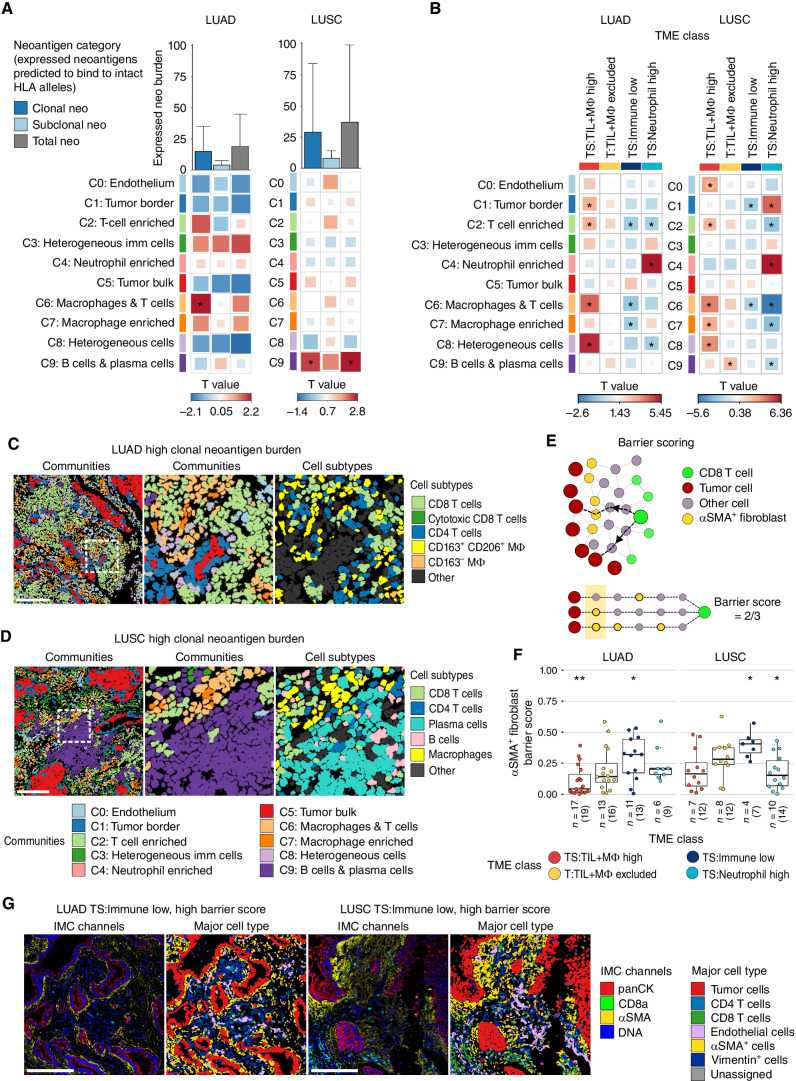
Spatial features associated with neoantigen burden and immune low TMEs. **A,** Correlation of densities of spatial cellular communities and the burden of expressed clonal, subclonal and total neoantigens predicted to bind intact HLA alleles, after accounting for HLA LOH, in LUAD (*n* = 31, 51 tumor cores) and LUSC (*n* = 17, 37 tumor cores). Bar plot shows the median neoantigen burden with whiskers extending to the 75th percentile. **B,** Comparison of the densities of spatial cellular communities in a given TME class compared with all other TME classes combined. LUAD: *n* = 21 *TS:TIL+M*Φ* high* cores, *n* = 20 *T:TIL+M*Φ* excluded* cores, *n* = 13 *TS:Immune low* cores, *n* = 11 *TS:Neutrophil high* cores. LUSC: *n* = 13 *TS:TIL+M*Φ* high* cores, *n* = 12 *T:TIL+M*Φ* excluded* cores, *n* = 8 *TS:Immune low* cores, *n* = 15 *TS:Neutrophil high* cores. Box sizes in **A** and **B** correspond to T values. **C,** Community and cell subtype maps from a LUAD tumor core with a high burden of expressed clonal neoantigens and high densities of *C2:T-cell enriched* and *C6:macrophage and T cells* communities. **D,** Community and cell subtype maps from a LUSC tumor core with a high burden of expressed clonal neoantigens and high densities of community *C9:B cells and plasma cells*. Single cells in **C** and **D** are colored by community according to the color legend below **D** or cell subtype as indicated. Scale bars, 200 μm. Middle, an enlargement of the area highlighted with a white box in the left plot with matched cell subtypes shown in the right plot. **E,** Schematic of αSMA^+^ fibroblast barrier score calculation. The barrier score measures the degree of spatial interpositioning of tumor cell–adjacent αSMA^+^ fibroblasts between CD8 T cells and their nearest tumor cell(s) in a tissue core. In the lower half of the schematic, three nearest tumor cells are defined for the green CD8 T cell, all six hops away. Tumor cell–adjacent αSMA^+^ fibroblasts are found on two of these three paths from CD8 T-cell to tumor cell, resulting in a barrier score of ⅔. **F,** Boxplot comparing the αSMA^+^ fibroblast barrier scores in a given TME class compared with all other TME classes combined in LUAD (*n* = 36, 57 tumor cores) and LUSC (*n* = 22, 45 tumor cores). Boxplots show median and lower and upper quartile values, and whiskers extend up to 1.5 × IQR above and below the quartiles. **G,** Representative IMC images and cell type maps from LUAD and LUSC tumor cores classified as *TS:Immune low* with a high barrier score. Scale bars, 200 μm. *P* values in **A**, **B**, and **F** and T values in **A** and **B** were calculated in a linear mixed-effects model with patient as a random effect, using smoking status as a fixed effect in **A** with a *P* value < 0.05 considered significant. LUAD, lung adenocarcinoma; LUSC, lung squamous cell carcinoma; panCK, pancytokeratin; TS, tumor/stroma; T, tumor; TIL, tumor-infiltrating lymphocytes; MΦ, macrophage; *, *P* < 0.05; **, *P* < 0.01.

We first correlated IMC-derived densities of cellular communities with the number of expressed neoantigens predicted to bind intact HLA alleles (Methods). We identified histology-specific correlations between spatial communities and neoantigen burden, correcting for multiregion sampling and patient smoking status. In LUAD, clonal neoantigen burden was associated with community *C6:macrophages and T cells* (linear mixed-effects model, LME *P* = 0.04), a community enriched in *TS:TIL+M*Φ* high* TMEs and depleted in *TS:Immune low* TMEs (Fig. 3A–C). Community *C6:macrophages and T cells* was characterized by increased densities of several CD4 and CD8 T-cell populations relative to other communities, including cytotoxic CD8 T cells and CD8 T resident memory (Trm) cells; however, enrichment of CD163^−^ and CD163^+^CD206^+^ macrophage populations distinguished *C6:macrophages and T cells* from *C2:T-cell enriched* (Supplementary Fig. S3F). A minority of cell subtypes were significantly associated with clonal neoantigen burden when considered independently of community localization (Supplementary Fig. S6A), suggesting the local niches in which they reside are relevant to understanding the antitumor immune response.

In LUSC, the burden of expressed clonal and total neoantigens predicted to bind intact HLA alleles was correlated with *C9:B cells and plasma cells* (LME *P* < 0.03), a community enriched in *T:TIL+M*Φ* excluded TMEs* and depleted in *TS:Neutrophil high* TMEs (Fig. 3A, B, and D). Both B cells and plasma cells were enriched in community *C9:B cells and plasma cells*, but only stroma-localized plasma cell densities were significantly correlated with clonal and total neoantigen burden in LUSC (Supplementary Figs. S3F and S6A). This spatial analysis reveals that plasma cells and the community in which they reside are associated not only with high TMB but also with the burden of clonal and total neoantigens in early-stage LUSC.

Of note, the T-cell–enriched community, which harbored CD4 T cells, CD8 T cells, and B cells, was only associated with the burden of clonal neoantigens in LUAD and subclonal neoantigens in LUSC when HLA LOH was not considered (HLA LOH-uncorrected, LME *P* < 0.018; Supplementary Fig. S6B). The T-cell–enriched community was also significantly correlated with stromal and tumor-infiltrating Tregs in LUSC, which may suggest hindered antitumor immunity in regions with a high burden of subclonal neoantigens (HLA LOH-uncorrected, LME *P* = 1.9e−05, LME *P* = 0.015; Supplementary Fig. S6C). Communities associated with HLA LOH-uncorrected neoantigen burden, including the T-cell enriched community, were enriched in *TS:TIL+M*Φ* high* TMEs, adding further resolution on cell organization in inflamed environments (LME *P* < 0.02; Fig. 3B; Supplementary Fig. S6B).

We next sought to establish the spatial immune context associated with HLA LOH and other somatic disruptions to HLA class I APM genes, hereafter referred to as class I/APM disruption (Methods). In LUAD, tumor regions with class I/APM disruption had increased densities of communities *C6:macrophages and T cells* and *C7:macrophage enriched* (LME *P* = 0.001, LME *P* = 0.023; Supplementary Fig. S6D). In LUSC, class I/APM disruption was observed more frequently in *TS:TIL+M*Φ* high* tumor cores compared with other TME classes (91% vs. 60% cores with available HLA data; Supplementary Fig. S6E).

These results uncover spatial niches of immune cells in high neoantigen burden tumors, including the spatial context of effector CD8 T-cell populations. Consideration of HLA LOH identified different spatial communities that are further affected by neoantigen clonality and NSCLC histology subtype.

### Peritumoral αSMA^+^ Fibroblasts Spatially Separate CD8 T Cells and Tumor Cells in Immune-Low TMEs

The exclusion of T cells from the tumor nest has been associated with the limited efficacy of immunotherapies. Previous work in NSCLC has found that dense fibrous stroma surrounding tumor islets in human lung tumor slices can limit T-cell ingress, likely mediated by distinct collagen-producing cancer-associated fibroblast (CAF) subsets (19, 20) and that the geometrical complexity of the tumor–stroma interface has been shown to be increased with overall low compared with high lymphocytic infiltrate (21). Both of these studies highlight a potential role for CAFs in T-cell exclusion in NSCLC. However, the diversity of cell subtypes interrogated in these works was limited.

Building on this, we harnessed the αSMA marker on our T cells and stroma antibody panel and explored whether immune TME classes were associated with distinct αSMA^+^ fibroblast arrangements, which may represent potential barriers to tumor–immune engagement. Using a barrier metric derived from constructing a cellular spatial graph for each tumor core (Fig. 3E, Methods), we found that *TS:Immune low* TMEs were characterized by a higher degree of physical occlusion of CD8 T cells from tumor cells by tumor-adjacent αSMA^+^ fibroblasts than other TMEs combined in both LUAD and LUSC (LME *P* = 0.02, *P* = 0.04, respectively, Fig. 3F and G). Barrier score distributions across TME classes did not reflect overall densities of αSMA^+^ fibroblasts in tumor cores (Supplementary Fig. S6F).

However, notably, αSMA^+^ fibroblast barriers were insufficient to explain the lack of immune cell infiltration into tumor nests in the *T:TIL+M*Φ* excluded* TME class in LUAD or LUSC, although high barrier scores were noted in individual cases (Fig. 3F). Nevertheless, cytotoxic CD8 T cells and leukocytes-other had significant avoiding relationships with tumor cells in *T:TIL+M*Φ* excluded* TMEs compared with other TME classes in LUAD and LUSC, respectively (Supplementary Fig. S6G).

Collectively, these results suggest that peritumoral αSMA^+^ fibroblasts may represent a feature of early-stage NSCLC TMEs with overall low levels of immune infiltration and a putative physical barrier to CD8 T-cell and tumor cell engagement.

### Tumors Infiltrated with Neutrophils and Sparse T Cells Are Metabolically Rewired and Distant from Vasculature

Immune classification revealed a distinct TME class with high neutrophil cell densities and sparse TIL infiltration in the tumor nest and stroma, *TS:Neutrophil high*, which was detected in at least one core in 28% of NSCLCs (*n* = 18/64 tumors with defined TME). Studies of NSCLC showing a neutrophil gene signature as the strongest immune predictor of mortality (22) and high neutrophil content inversely correlating with T-cell infiltration (23) led us to investigate a potential tumor-promoting role of this TME class. Of note, neutrophil infiltration levels in LUSC were higher than in LUAD tumor cores by a factor of two (median of 752 cells/mm^2^ compared with 343 cells/mm^2^, respectively); therefore, the *TS:Neutrophil high* TME class was examined separately and compared between the major histologic subtypes.

**Figure 4.**
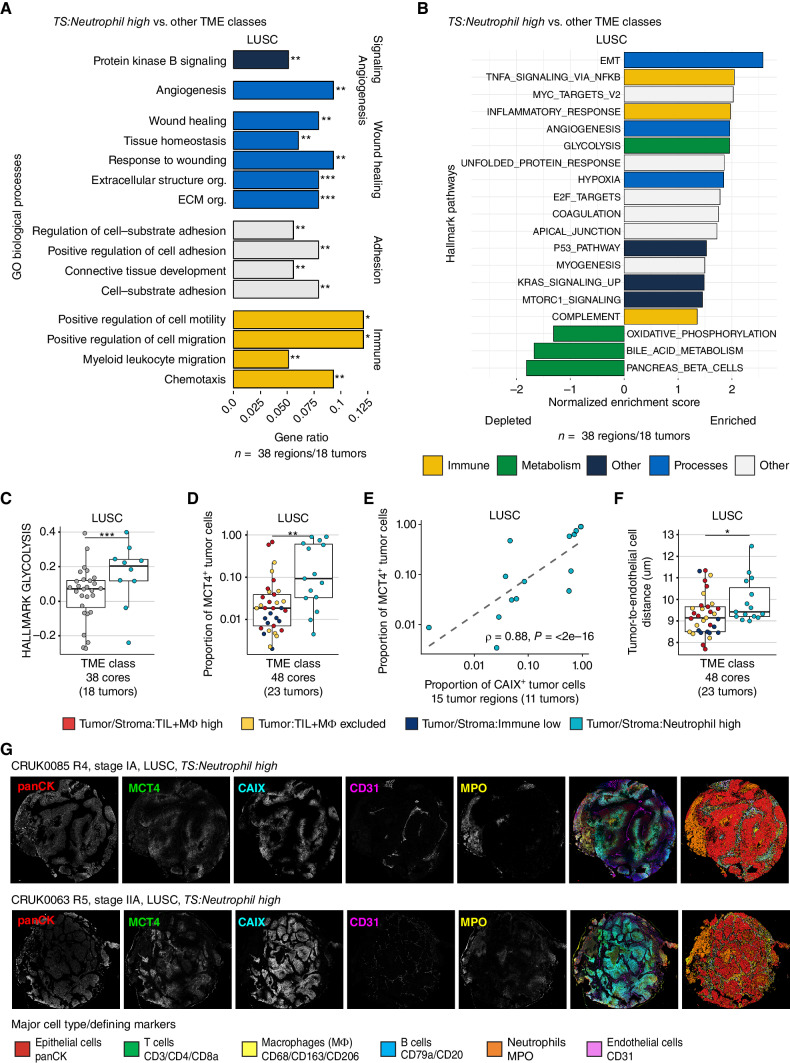
Neutrophil infiltration in LUSC is associated with distinct metabolic and immunosuppressive phenotypes. **A,** Gene Ontology (GO) biological processes enriched among upregulated genes in the *TS:Neutrophil high* TME class (*n* = 10 cores) compared with other TME classes combined (*n* = 28 cores) in LUSC (FDR < 0.01, gene ratio > 0.05). **B,** GSEA of hallmark gene sets compared between tumor cores from the *Tumor/Stroma:Neutrophil high* TME class and other TME classes combined, using the t-statistic derived from the limma–voom model on TMM-normalized gene expression. Significantly enriched pathways were colored by type of pathway (FDR < 0.05). **C,** Normalized enrichment score derived from single-sample GSEA visualized for *TS:Neutrophil high* and cores from other TME classes. The *P* value is derived from GSEA of LUSC as shown in **A** and adjusted for other hallmark pathways using the Benjamini–Hochberg method. **D,** Proportion of tumor cells assigned MCT4^+^ in *TS:Neutrophil high* tumor cores compared with tumor cores from other TME classes combined in LUSC. **E,** Spearman correlation coefficient and *P* value comparing the proportion of MCT4^+^ and CAIX^+^ tumor cells in *TS:Neutrophil high* LUSC TMEs. **F,** Median distance between LUSC tumor cells to their nearest endothelial cell per core in *TS:Neutrophil high* TME class compared with all other TME classes combined. **G,** Single-channel images and composite image alongside cell type map displaying tumor cells, neutrophils (MPO, yellow), endothelial cells (CD31, magenta), and regions of hypoxia (CAIX, cyan) and MCT4 (green) expression. Boxplots show median and lower and upper quartile values, and whiskers extend up to 1.5 × IQR above and below the quartiles. *P* values for **D** and **F** were calculated in a linear mixed-effects model with patient as the random-effect covariate. LUSC, lung squamous cell carcinoma; TS, tumor/stroma; FDR, false discovery rate; TMM, trimmed mean of *M*-values; panCK, pancytokeratin; *, *P* < 0.05; **, *P* < 0.01; ***, *P* < 0.001.

We assessed signaling pathways that were differentially regulated in this TME class, using paired TRACERx 100 RNA-seq data (*n* = 18 tumors, 38 regions). In LUSC, overrepresentation analysis of gene ontology (GO) biological processes showed upregulation of several processes, including protein kinase B (PKB) signaling, angiogenesis, and transcriptional programs associated with wound healing and myeloid leukocyte migration in *TS:Neutrophil high* tumor cores relative to cores from other TME classes (FDR < 0.01, Fig. 4A; Supplementary Fig. S7A). Additionally, we identified hallmark gene sets enriched in *TS:Neutrophil high*, which included epithelial–mesenchymal transition (EMT) and hypoxia (Fig. 4B). Although several metabolic processes including oxidative phosphorylation (OXPHOS) were significantly downregulated, glycolysis was upregulated in *TS:Neutrophil high* cores compared with those with other TME classes (FDR = 3e−06; Fig. 4C), suggesting metabolic reprogramming of the tumor cells in this class.

Tumor cells can increase glycolytic activity (24, 25) and upregulate the lactate transporter, MCT4, to shuttle excess lactate, a product of glycolysis, into the microenvironment (26). Therefore, we compared the levels of MCT4 expression on tumor cells and observed a greater proportion in *TS:Neutrophil high* than other TME classes in LUSC (LME *P* = 0.002; Fig. 4D). MCT4 expression by nontumor epithelial cells was negligible (Supplementary Fig. S7B). Therefore, LUSC tumors with *TS:Neutrophil high* TMEs downregulated the OXPHOS transcriptional program, while increasing MCT4^+^ protein expression, likely leading to increased glycolytic activity and lactate accumulation in the TME.

Nutrient-restrictive and hypoxic conditions can drive cancer cells to switch to substitute energy sources (27). We, therefore, compared the presence of CAIX, a hypoxia-induced enzyme on tumor cells, and found that the proportion of CAIX^+^ tumor cells in the *TS:Neutrophil high* TME class was not significantly higher than other TME classes (Supplementary Fig. S7C). However, the proportion of MCT4^+^ tumor cells strongly correlated with the proportion of CAIX^+^ tumor cells in LUSC in the *TS:Neutrophil high* TME class (ρ = 0.88, *P* < 2e−16, Fig. 4E). We evaluated the proximity to vasculature as the median distance between each tumor cell to its nearest endothelial cell and found a significantly greater distance in the *TS:Neutrophil high* class compared with other classes in LUSC (Fig. 4F and G). The tumor proliferation levels assessed as the proportion of Ki-67^+^ tumor cells were lower in *TS:Neutrophil high* relative to other TME classes in LUSC (Supplementary Fig. S7D). These results suggest that the increase in glycolytic activity in this TME class is associated with a decrease in oxygen supply in tumors at a larger distance to vasculature.

Restricted vascular access consistent with a larger distance between tumor and endothelial cells can result in tumor necrosis. The presence of necrosis, as evaluated by histopathologic review of paired IMC and H&E images (Methods), was frequently detected in *TS:Immune low* (75% of cores) and *TS:Neutrophil high* (60%) TMEs in LUSC (23%–28% other TMEs; Supplementary Fig. S7E). Notably, the presence of necrosis alone was not associated with increased tumor–endothelial cell distance nor fraction of MCT4^+^ tumor cells (LME n.s.).

The *TS:Neutrophil high* class in LUAD was also characterized by a larger distance of tumor cells from endothelial cells than in other TME classes (*P* = 0.01, LME model; Supplementary Fig. S7F) and a higher frequency of necrosis in 36% of cores compared with 4.8% to 7.7% in other TMEs (chi-squared test *P* = 0.01; Supplementary Fig. S7G). As observed for LUSC, gene set enrichment analysis (GSEA) of LUAD also showed upregulation of the hallmark gene sets EMT and KRAS signaling, and several metabolic processes including OXPHOS were downregulated (*n* = 28 tumors, 49 regions; Supplementary Fig. S7H). However, the glycolysis hallmark gene set was depleted, and the proportion of MCT4^+^ and CAIX^+^ tumor cells were not significantly different in the *TS:Neutrophil high* TME class compared with other TME classes in LUAD (Supplementary Fig. S7I–S7J). Overall, LUAD tumor cores had a significantly lower proportion of CAIX^+^ tumor cells compared with LUSC (Supplementary Fig. S7K). These results suggest different metabolic and hypoxic environmental cues in the *TS:Neutrophil high* TME class between LUAD and LUSC.

In both LUAD and LUSC, the *TS:Neutrophil high* class had sparse TIL infiltration (Supplementary Fig. S5D). Spatial cell-to-cell interaction analyses revealed an avoiding relationship between neutrophils and cytotoxic CD8 T cells in this TME class more frequently than in other TME classes in LUAD and LUSC. No other significant interactions between neutrophils and any of the identified cell subtypes were observed (Supplementary Fig. S7L), suggesting immunosuppression in the proximity of neutrophils in both histologic subtypes.

In summary, we identified a distinct TME class defined by predominant infiltration of neutrophils, increased distance from tumor to vasculature, upregulation of EMT, and metabolic rewiring of cancer cells in both LUAD and LUSC. Although the metabolic cues may differ between LUAD and LUSC, *TS:Neutrophil high* represented an immunosuppressed TME with sparse TIL infiltration in both histologic subtypes.

### Gain-of-Function Mutations in Phosphoinositide 3-Kinase (PI3K) Signaling Implicated in Neutrophil Recruitment in LUSC Tumors

Cancer cell–intrinsic signaling can regulate tumor metabolism, immunosuppression, and angiogenesis in NSCLC (28, 29). Here, we set out to dissect genomic aberrations enriched in tumors with a *TS:Neutrophil high* TME that potentially enhance fitness, modulate inflammation, or support glycolytic activity. Therefore, we examined whether somatic alterations in components of the transcriptionally upregulated pathways, such as KRAS and PKB signaling (Fig. 4A and B; Supplementary Fig. S7H), were frequently enriched in tumor cores with a *TS:Neutrophil high* TME.

**Figure 5.**
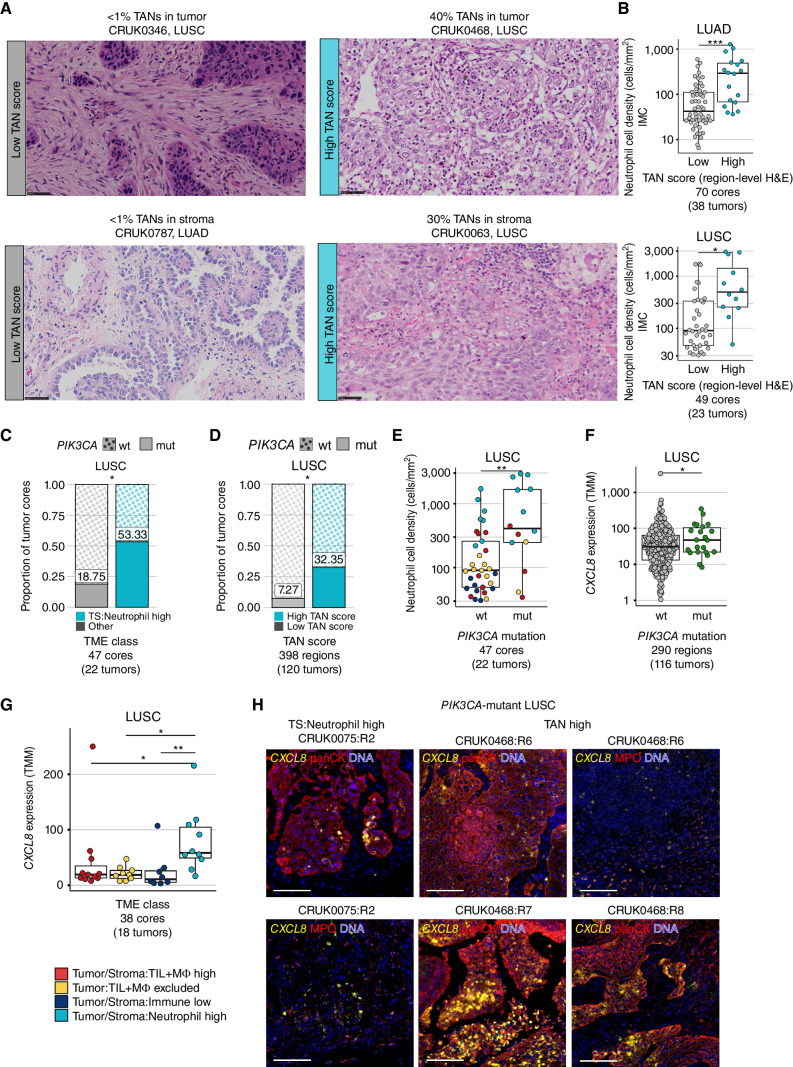
Neutrophil-rich TMEs are associated with activating mutations in PI3K and tumor-intrinsic *CXCL8* upregulation. **A,** Representative crops of tumor-level H&E images with low TAN scores in the tumor nest and stroma (left) and high TAN scores in the tumor nest and stroma (right), inferred as the proportion of the neutrophil area in tumor/stroma from the total tumor/stroma tissue area. Scale bar, 50 μm; 400× magnification. **B,** Neutrophil cell density as defined by IMC compared between region-level TAN-low versus TAN-high tumor cores based on H&E scores in LUAD and LUSC. **C** and **D,** Proportion of tumor cores with (mut) and without (wt) *PIK3CA* driver mutations compared between *TS:Neutrophil high* versus other TME classes combined (**C**) and region-level TAN-High versus TAN-Low cores (**D**) in LUSC. *P* values were derived from a Chi-square test. **E,** Neutrophil cell density by *PIK3CA* mutation status, points colored by TME class assignment. **F** and **G,** TMM expression values for *CXCL8* compared by *PIK3CA* mutation status (**F**) and between TME classes (**G**) in LUSC. **H,** Immunofluorescence images of *CXCL8* RNAscope multiplexed with antibody staining of pancytokeratin (panCK) or MPO in an LUSC tumor region with a *TS:Neutrophil high* TME and subclonal *PIK3CA* mutation, and an LUSC patient with multiple TAN-high tumor regions and a clonal *PIK3CA* mutation. panCK and MPO examples for CRUK0075:R2 illustrate the same region of interest, whereas different regions of interest are shown for CRUK0468:R6. Scale bar, 100 μm. *P* values for **B** and **E** were calculated in a linear mixed-effects model with patient as the random-effect covariate. *P* values in **F** and **G** were derived from a limma–voom differential expression analysis correcting for multiple regions per tumor. **·**, *P* < 0.1; *, *P* < 0.05; **, *P* < 0.01; ***, *P* < 0.001; LUAD, lung adenocarcinoma; LUSC, lung squamous cell carcinoma; TMM, trimmed mean of *M*-values; TS, tumor/stroma; TIL, tumor-infiltrating lymphocyte; MΦ, macrophage; TAN, tumor-associated neutrophils; H&E, hematoxylin and eosin; mt, mutant; wt, wild-type.

To systematically examine driver mutations and copy-number changes, we expanded our TRACERx 100 cohort to TRACERx 421 (14). We used pathologist-derived tumor-associated neutrophil (TAN) scoring from H&E images of paired regional blocks of the study TMAs (region-level TAN score) and tumor-matched diagnostic blocks (tumor-level TAN score). The TAN scoring approach evaluated the TANs in the tumor nest and stroma adapting a standardized method used for TIL quantification (Fig. 5A; Methods; ref. 30). Tumor cores were stratified into TAN-high and TAN-low using the *TS:Neutrophil high* TME as a reference for high TAN scores (Methods). H&E-derived TAN scores recapitulated the presence of neutrophils derived from paired IMC in LUAD and LUSC (Fig. 5B; Supplementary Fig. S8A, Spearman correlation ρ = 0.5–0.6, *P* < 2.5e−07). The probability of detecting a TAN-high TME across all sampled regions was 0.5 in TAN-high tumors (Methods), equal to the probability estimated for the *TS:Neutrophil high* TME class. Notably, TAN-high tumor regions were enriched for a TAN transcriptional signature (31) and had a higher proportion of IMC-derived PD-L1^+^ neutrophils and MCT4^+^ tumor cells compared with TAN-low LUSC tumors (Supplementary Fig. S8B–S8D).

Using matched genomic profiles, we assessed whether activating driver mutations in components of the PKB/PI3K–AKT signaling pathway were enriched in the *TS:Neutrophil high* TME class of LUSC. We found a higher frequency of gain-of-function *PIK3CA* driver mutations within the *TS:Neutrophil high* TME class compared with other TMEs (*P* = 0.04, 53% vs. 19% cores; Fig. 5C). We further validated this observation in the extended TRACERx 421 cohort using TAN scores (*n* = 398 cores, 120 LUSC tumors). Driver *PIK3CA* mutations were significantly enriched in TAN-high tumors compared with TAN-low tumors (*P* = 0.03, 25% vs. 5% tumors, tumor level) and detected in 32% of TAN-high compared with 7% of TAN-low tumor regions (Fig. 5D). Copy-number analysis using GISTIC2.0 revealed no evidence of copy-number amplifications and deletions in driver and immune evasion genes enriched only in the TAN-high compared with TAN-low tumor regions (Supplementary Fig. S8E).

Nearly all *PIK3CA* mutations were of clonal origin in LUSC (*n* = 46/53 regions), indicating that these mutations were detected in all tumor cores. Tumors with a clonal *PIK3CA* mutation had a twice higher probability of having a TAN-high TME compared with *PIK3CA* wild-type tumors (0.47 vs. 0.25, adjusted for the number of sampled regions). Given that tumor cores with a *PIK3CA* mutation had significantly higher neutrophil cell densities than *PIK3CA* wild-type cores in LUSC (LME *P* = 0.005, Fig. 5E), we examined neutrophil-attracting chemokines as defined with the GO term neutrophil chemotaxis (Methods). We compared their expression between tumor cores with a *TS:Neutrophil high* TME and those with other TMEs as well as between *PIK3CA* mutant and *PIK3CA* wild-type tumor regions in the expanded TRACERx 421 cohort. The only chemokine unregulated in both comparisons was IL-8, encoded by the gene *CXCL8* (Supplementary Fig. S9A). *CXCL8* was more highly expressed in *PIK3CA* mutant vs. *PIK3CA* wild-type tumor cores (*P* = 0.01, *n* = 290 regions, Fig. 5F). Higher expression levels of *CXCL8* in *PIK3CA* mutant versus *PIK3CA* wild-type LUSC was validated in The Cancer Genome Atlas (TCGA) data set (*n* = 464, *P* = 0.01; Supplementary Fig. S9B). *CXCL8* showed the highest expression in the *TS:Neutrophil high* TME compared with other TMEs (*P* = 0.004, 1.4 log fold change; Fig. 5G) and correlated with neutrophil cell density (ρ = 0.6, *P* = 3.5e−05; Supplementary Fig. S9C). We performed *CXCL8* RNA *in situ* hybridization to determine whether tumor cells expressed the chemokine in four *PIK3CA* mutant cores and two *PIK3CA* wild-type cores with *TS:Neutrophil high* TMEs or high TAN scores. In addition to *CXCL8* expression by neutrophils, as indicated by MPO costaining, *CXCL8* expression was detected predominantly within tumor cells, as indicated by panCK costaining, in LUSC cores with *TS:Neutrophil high* or TAN-high TMEs independently of the *PIK3CA* mutation status (Fig. 5H; Supplementary Fig. S9D). *CXCL8* expression correlated positively with glycolytic markers in TRACERx 100 and TCGA LUSC cohorts (Supplementary Fig. S9E).

Following the observed upregulation of KRAS signaling from GSEA, we also assessed whether activating mutations in *KRAS* were enriched in cores with *TS:Neutrophil high* TMEs. *KRAS* mutations, which were absent in LUSC cores, were found in 50% of LUAD cores with this TME class and 41% of the *TS:TIL+M*Φ* high* class compared with 14% to 18% in other classes (n.s., Supplementary Fig. S9F), suggesting that any potential immunomodulatory effects of *KRAS* driver mutations observed in mouse models in LUAD (32) are not limited to the *TS:Neutrophil high* TME.

In summary, somatic driver mutations in *PIK3CA* in LUSC and transcriptional activation of KRAS signaling in LUAD and LUSC were associated with neutrophil infiltration.

### TANs Infiltrate Regions with Expanded Tumor Subclones and Predict Poor Clinical Outcome in NSCLC

**Figure 6.**
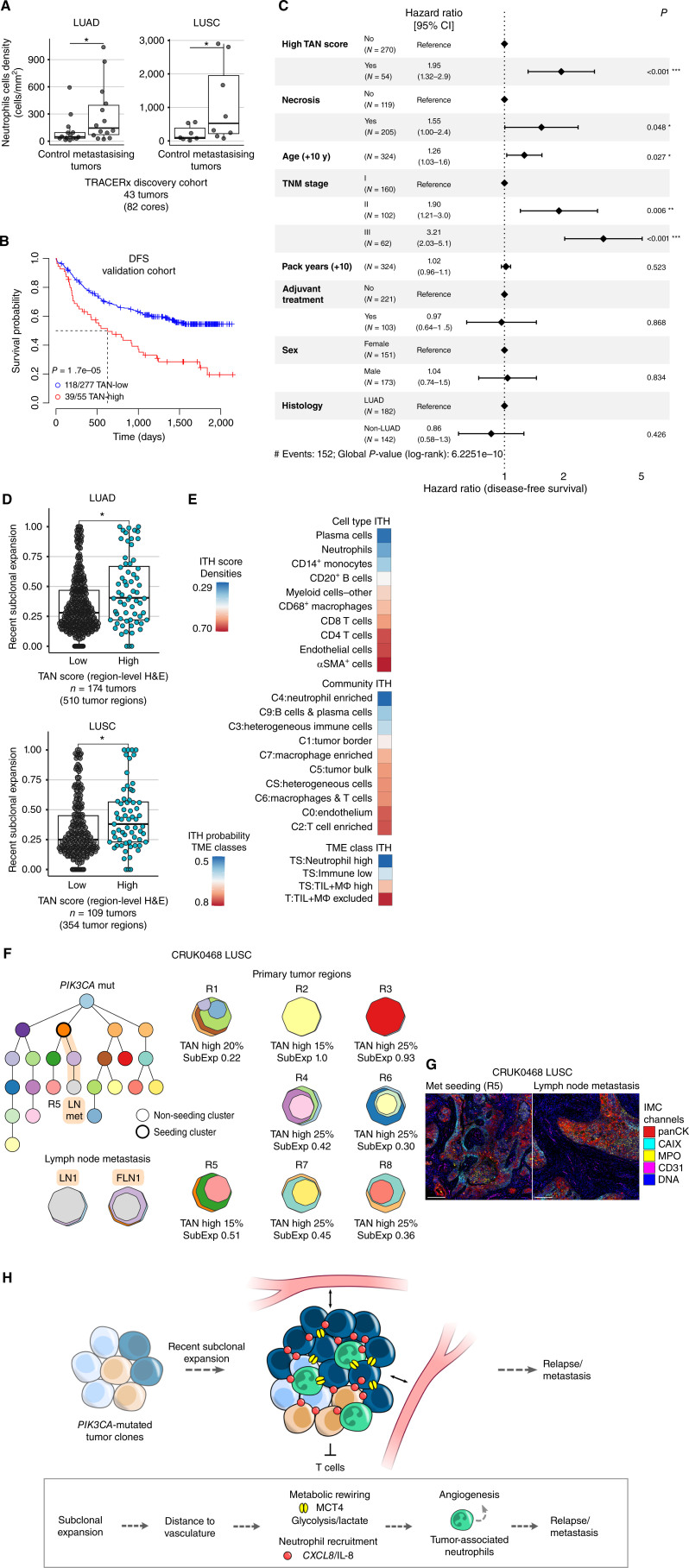
Neutrophil infiltration is associated with recent subclonal expansion and poorer disease-free survival. **A,** Comparison of neutrophil cell density in primary tumors with metastasis-seeding clones detectable at time of surgery or during follow-up (metastasizing tumors) to tumors from patients who were metastasis-free and recurrence-free for more than 3 years of follow-up time (control) in LUAD (*n* = 28) and LUSC (*n* = 15) within the TRACERx discovery cohort. Maximum neutrophil density taken for tumors with multiple tumor cores. *P* values derived from one-tailed Wilcoxon test. **B,** Kaplan–Meier curves for DFS according to tumor-level TAN score in the validation cohort (*n* = 332 patients). *P* value derived from univariate Cox model adjusted for histology. **C,** Multivariable Cox proportional hazard regression analysis of DFS using tumor-level TAN score and tumor-level necrosis evaluation from H&E images of diagnostic tumor blocks. **D,** Recent subclonal expansion score, measured as the maximum cancer cell fraction of subclones at the terminus of the phylogenetic tree, compared between TAN-high and TAN-low tumor regions in LUAD and LUSC patients from the TRACERx 421 cohort. *P* values were derived from a linear mixed effects model with patient as random effect. **E,** Spatial ITH score of cell types and communities and ITH probabilities of TME classes in multiregion analysis (pan-immune *n* = 41 tumors, 112 cores). ITH score was calculated as the average standard deviation of the cell/community density in multiple regions per tumor and z-score transformed. ITH score of αSMA^+^ cells was derived from T cells and stroma panel (*n* = 39 tumors, 105 cores). ITH probability was calculated as 1 − probability of all regions having the same indicated TME class. **F,** Example phylogenetic tree depicting a stage IIIA LUSC case with a clonal *PIK3CA* mutation, high TAN scores, and recent subclonal expansion, including in a region (R5) that seeded a lymph node (LN) metastasis (FLN, FFPE LN). The metastasis-seeding lineage is highlighted in orange. Tumor-level TAN scores and regional subclonal expansion (SubExp) scores are reported for primary tumor regions. The reported TAN score represents the maximum of the tumor nest and stroma scores. Each cluster in the phylogenetic tree is assigned a color that is also represented in the region clone maps. The clone maps illustrate the prevalence of each clone within a region. **G,** IMC images shown for R5 and the LN metastasis. Scale bar, 200 μm. **H,** Summary schematic of the link between tumors with a neutrophil enriched microenvironment with tumor progression. **·**, *P* < 0.1; *, *P* < 0.05; **, *P* < 0.01; ***, *P* < 0.001; LUAD, lung adenocarcinoma; LUSC, lung squamous cell carcinoma; ITH, intratumor heterogeneity.

We set out to assess the propensity of tumors with different TMEs to evolve, expand, and metastasize. Given the protumor features associated with neutrophil-rich TMEs and previously reported implications of neutrophils in metastasis in animal models (33–35), we investigated whether neutrophil infiltration was linked to the risk of disease relapse and metastasis in NSCLC. Using clonal analysis of TRACERx primary tumor regions and paired metastases, we identified the clones that seeded metastases (15). We compared neutrophil cell densities using IMC data between primary tumors with metastasis-seeding clones detectable at the time of surgery or during follow-up (metastasizing tumors) to tumors from patients who remained metastasis-free and recurrence-free with a minimum of three years of follow-up (discovery cohort, *n* = 43 LUAD and LUSC patients; Supplementary Fig. S10A). We observed a significant increase in neutrophil cell densities in the metastasizing tumors compared with tumors from metastasis- and recurrence-free patients, in LUAD (*P* = 0.03) and LUSC (*P* = 0.04 one-tailed test; Fig. 6A).

We endeavored to confirm this observation in a separate validation cohort within TRACERx using the H&E-derived TAN scoring (*n* = 332 patients; Supplementary Fig. S10A; Supplementary Table S3). We hypothesized that primary NSCLCs with a neutrophil-rich TME more frequently seeded metastases and, thus, had a higher risk of relapse after surgical removal of the primary tumor. Univariate disease-free survival (DFS) analysis showed a significant association between high tumor-level TAN scores and poorer DFS in NSCLC (*P* = 1.7e−05), with a median DFS of 21 months for high compared with longer than 70 months for low TAN score (Fig. 6B). This prognostic association was independent of the cutoff used to define TAN scores and was maintained for both the tumor-level and region-level TAN scores (Supplementary Fig. S10B and S10C; Methods). In addition, high tumor-level TAN score was also strongly associated with shorter DFS in LUAD (HR = 3.4, *P* = 2e−05) and LUSC (HR = 2.13, *P* = 0.01), separately (Supplementary Fig. S10C).

Given the higher frequency of necrosis in the neutrophil infiltrated tumors, which has been previously associated with advanced stage and worse prognosis in NSCLC (36), we next evaluated whether the TAN association with outcome was confounded by previously reported predictors of poor DFS. In a multivariable model of DFS using TAN score, necrosis, age, sex, adjuvant treatment, pack years, histology subtype, and TNM stage, TAN score was an independent prognostic factor (*P* < 0.001), alongside necrosis status (*P* = 0.048), age (*P* = 0.027), and stage (*P* ≤ 0.006) in NSCLC (Fig. 6C; Supplementary Fig. S10D and S10E).

To confirm the prognostic association between the TAN scoring approach and DFS, we validated the relationship in a second independent cohort using an automated approach for granulocyte quantification. We applied the deep-learning model PathExplore (37) for cell type and tissue classification on whole-slide images from TCGA. Similarly to the TAN score, tumors were assigned a high score when the granulocyte proportion in the tumor nest or stroma was higher than the respective tumor nest- or stroma cutoff (Supplementary Fig. S11A). As the high TAN score cutoffs derived from the TRACERx cohort were not transferable to the automated scores on the TCGA cohort (Methods), the association with DFS was evaluated across a range of cutoffs based on the tumor nest and stroma proportions. The pattern of high hazard ratios (increased risk of recurrence or death) was consistently seen with increasing granulocyte proportions in the tumor nest or stroma in multivariable analyses of DFS using age, sex, adjuvant treatment, pack years, histology subtype, and TNM stage (*n* = 109 patients with NSCLC, for which automated scores and information on factors used in the multivariable Cox regression model was available; Supplementary Fig. S11B), maintaining a statistical significance across all intervals above 3.1% tumor nest proportion and 2.5% stromal proportion (*P* value for the automated score in the multivariable model *P* < 0.05).

Finally, given the association of TAN score with poor outcome, we investigated whether neutrophil infiltrate was associated with evolutionary patterns previously shown to associate with shorter DFS in TRACERx (14). Specifically, we investigated a genomic measure of recent subclonal expansion in primary tumors, defined as the size of the largest subclone terminal to the phylogenetic tree of a tumor region. TAN-high tumor regions had significantly higher subclonal expansion scores than TAN-low tumor regions in LUAD and LUSC (*P* < 0.04; Fig. 6D), suggesting that neutrophil-rich TMEs have larger expansions of recent subclones compared with other tumor regions and are associated with dynamics of tumor evolution implicated in metastasis and poor outcome. The presence of *PIK3CA* mutations was also associated with a higher recent subclonal expansion score compared with *PIK3CA* wild-type tumors in LUSC (*P* = 0.03; Supplementary Fig. S11C).

The intratumoral heterogeneity of prognostic factors, potential therapeutic targets, or stratification biomarkers is of paramount importance for their clinical applicability and optimal therapy response. Using deep learning-based approaches, a recent LUAD study identified TME features in 1-mm^2^ LUAD cores that collectively predict poor prognosis (1). The model, however, relied on features that had high spatial heterogeneity in NSCLC (Fig. 6E), underscoring the importance of accounting for sampling bias in the design of clinically actionable assays. Neutrophils, their spatial community, and TME class had the lowest spatial heterogeneity, together with plasma cells, compared with other immune cell types and spatial communities (Fig. 6E).

Patient CRUK0468 diagnosed with stage IIIA LUSC, for example, harbors a TAN-high tumor with low spatial heterogeneity. All of the eight tumor regions sampled from the primary tumor had high neutrophil infiltrate with TAN-high score and clonal *PIK3CA* mutation. Seven regions showed a higher recent subclonal expansion score than the cohort median, including the region that seeded a lymph node metastasis, R5 (Fig. 6F). The seeding region, R5, and the seeded lymph node metastasis shared TME features: high infiltration of neutrophils and CAIX^+^ expression along the tumor nest edges, identified with IMC in the primary tumor and the metastasis region (Fig. 6G).

Based on these results, we propose LUADs and LUSCs with high TAN infiltration harbor recently expanded subclones and can be enriched for specific driver mutations acquired at the earliest stages of evolution, such as *PIK3CA* in LUSC (Fig. 6H). Tumors with high TAN infiltration had low spatial heterogeneity and a high risk of metastasis or relapse in NSCLC independent of stage, necrosis, and other clinical variables.

## DISCUSSION

In this integrative study of the TME in NSCLC, we used high-dimensional tissue imaging and paired sequencing to reveal cancer-intrinsic and -extrinsic features underlying TME composition and spatial organization. We identified neoantigen-directed immune dynamics, fibroblast organization, driver mutations, and immune phenotypes as potential determinants of TME architecture. These TME features, common across NSCLC or specific to histologic subtypes, reveal potential therapeutic vulnerabilities and challenges. We present a ranking of the spatial heterogeneity of these TME features, providing a reference for studies seeking to identify low spatial heterogeneity targets. The unique structure of the TRACERx study and its depth of clinical and genomic information enabled us to link the TME to subclonal expansion, the seeding of metastases, and clinical outcome.

We defined four distinct, pan-histology TME classes in early-stage treatment-naïve NSCLC, building on earlier classifications based on the spatial distribution of TILs alone (17) and compositional clustering from gene expression (38–40). We described 10 multicellular communities present across histologic subtypes of NSCLC, confirming those defined in a recent IMC study of LUAD, despite differences in the resolved cell subtypes and the histologic composition of each data set (1). TME classes captured intratumoral immune infiltration or exclusion from the tumor nest, whereas spatial communities revealed multicellular clusters that may represent functional niches.

One of the unifying features of LUAD and LUSC tumor cores was the enhanced spatial interpositioning of peritumoral αSMA^+^ fibroblasts between CD8 T cells and tumor cells in *TS:Immune low* TMEs. We propose that stromal barriers represent a potential physical barrier to tumor–T-cell engagement and may affect T-cell entry into tumors. These results support previous literature implicating CAFs in immunosuppression in different cancer types through both physical exclusion of immune cells from tumor nests and secretion of cytokines (20, 41, 42). However, the αSMA^+^ CAF barrier was insufficient to explain the immune-excluded TMEs captured in this study. In line with this finding, Li and colleagues found, using *in silico* modeling, that chemorepellent expression rather than fibrotic barriers best described short-range T-cell exclusion from tumor islets in triple-negative breast cancer (43). Future work should seek to improve segmentation and resolution of CAF subtypes within and beyond αSMA^+^ fibroblasts, as different CAF subtypes have recently been shown to have different prognostic power in treatment-naïve primary NSCLC (8) and to be differentially localized in the NSCLC TME (20).

Clonal neoantigen burden has been associated with improved responses to CPI (44, 45), warranting exploration of TME structures that may form in response to a high burden of clonal neoantigens. Here, we delineate spatial multicellular niches that increase in prevalence with clonal neoantigen burden and demonstrate that their composition varies depending on the clonality of expressed neoantigens, the LOH of the presenting HLA allele and the histologic subtype. In LUAD, a multicellular niche of macrophages and T cells may form in response to a high clonal neoantigen burden and influence the selection of cancer cell–intrinsic disruptions to MHC class I antigen presentation. Although this community harbored cytotoxic and resident memory CD8 T-cell populations, interactions between macrophages and CD8 T cells can inhibit T-cell motility and promote exhaustion (46, 47). Furthermore, exposure to clonal neoantigens has been associated with increased T-cell dysfunction in this data set (16). *TS:TIL+M*Φ** high LUAD TMEs harbored enrichment of this macrophage and T-cell community, which adds spatial resolution to previous associations between inflamed tumor regions and intrinsic immune escape (10). This underscores the need to characterize macrophage subsets in the proximity of T cells and how these can be targeted therapeutically to support a tumor-specific effector response directed against clonal neoantigens (48).

Plasma cells and their corresponding community were correlated with not only clonal but also total neoantigen burden in LUSC, as well as high TMB (≥10 mutations/Mb) in both LUAD and LUSC. These findings link several features associated with improved CPI response (44, 45, 49–52). Few papers have previously identified links between these cancer cell–intrinsic and –extrinsic features, reporting no association in studies of LUAD alone (53–55). The plasma cell community identified in TRACERx may have formed independently of tertiary lymphoid structures (TLS), as we did not identify TLS in the regional blocks from which TMA cores were derived (Methods). This indicates that the tumor-infiltrating plasma cells may have derived from the tumor-draining lymph node or that TLS were not captured in the tissue analyzed. These associations underscore the importance of dissecting antibody responses to neoantigens as well as deregulated self-antigens (53) and their impact on CPI responses.

This work further revealed that high infiltration of neutrophils was associated with metastasizing tumors and shorter DFS in NSCLC. Although the peripheral neutrophil-to-lymphocyte ratio has been linked to adverse outcomes in NSCLC (56), reports on the prognostic impact of local, intratumoral neutrophils have been conflicting. Among immune cells, neutrophil transcriptional signatures have been shown as the strongest predictor of poor prognosis in NSCLC (22). However, a variable association of intratumoral neutrophils with clinical outcomes has been reported depending on the histologic subtype (57), neutrophil location (58), and their phenotype (1). Here, we developed and validated a TAN scoring approach from routinely collected H&Es that represent *TS:Neutrophil high* TMEs by equally weighting the proportion of neutrophils in the tumor nest or stroma. In both LUAD and LUSC, this clinically accessible TAN score is a robust prognostic factor independent of stage and other clinical variables in two independent cohorts.

TAN-high tumors also displayed low spatial heterogeneity, a feature that is essential for overcoming sampling bias. We observed high spatial heterogeneity of TME features previously used to predict prognosis in LUAD with a deep-learning approach, which failed to maintain the level of prediction accuracy when the number of lineage markers was reduced (1). Spatial heterogeneity of cell types used in unsupervised deep-learning models may represent an obstacle to the translation of deep-learning approaches to clinically accessible assays. The low spatial heterogeneity observed for neutrophils and the spatial structures they formed support the translational potential of the proposed TAN scoring approach.

The potential mechanisms behind the prognostic association of TANs remain to be defined. Mounting evidence depicts neutrophils as highly plastic and adaptable to the tissue context (59). Therefore, studying the spatial setting of neutrophils is fundamental to determining the environmental cues that modulate antitumor and protumor phenotypes. Neutrophils can exert protumorigenic features through promotion of extracellular matrix degradation, EMT, angiogenesis, immunosuppression, and metastasis (33, 60, 61), all of which were associated with the *TS:Neutrophil high* TME class in at least one of LUAD or LUSC. Upregulation of EMT transcriptional programs in tumor cells neighboring neutrophil clusters was also observed in a recent single-cell transcriptomics study (62). In addition, tumor cores with *TS:Neutrophil high* TMEs in LUAD and LUSC had altered metabolic cancer cell states. However, these metabolic states and the hypoxic conditions in this class differed between LUAD and LUSC, in line with previous reports on metabolic differences between the two histologic subtypes (63). The upregulation of MCT4 and CAIX expression in LUSC suggests that hypoxia or lactate efflux may contribute to the immunosuppressed phenotype in this class, for example, through the promotion of PD-L1 expression and T-cell anergy in settings of inflammation (63, 64), consistent with the increased frequency of PD-L1^+^ neutrophils.

In this study, we showed that TANs were enriched in tumor regions with recent expansions of tumor subclones, revealing a link between the TME and the evolutionary history of the tumor in LUAD and LUSC. This association was independent of the presence of necrotic areas and proportions of MCT4^+^ and CAIX^+^ tumor cells in LUAD and LUSC, implicating neutrophils as indicators or promoters of recurrence and metastasis (64). Although neutrophils have been described as the mediators of indirect subclonal cooperation but not expansion that promotes metastasis in a breast cancer model (33), the results of this study suggest tumor evolution dynamics are linked to metastasis in neutrophil enriched tumors. As a result of, for example, tumor subclonal expansion outpacing its vascular supply, metabolic stress can trigger necrosis and neutrophil recruitment, which can be further amplified in a positive feedback loop. Within this spatial context, neutrophils acquired a TAN phenotype. Therefore, neutrophils distinguish a unique microenvironment associated with disease progression, which could not be explained by, for example, necrosis, hypoxia, or glycolysis alone in LUAD and LUSC. Based on these findings, we reason that TAN-High LUADs and LUSCs likely orchestrate a protumor TME to compensate for restrictive vascular access and metabolic dysregulation and polarize neutrophils to a proangiogenic phenotype that results in immunosuppression, a higher risk of metastasis, and subclonal expansions.

Upregulation of oncogenic signaling pathways was also associated with the neutrophil enriched TME, such as KRAS signaling in LUAD and LUSC. We previously showed, in a LUAD mouse model, that tumors with a *KRAS*^G12C^ mutation had neutrophil aggregates and excluded TILs, whereas treatment with the KRAS inhibitor MRTX1257 resulted in reduced neutrophil and increased T-cell infiltrates (65). However, *KRAS* driver mutations were not only enriched in *TS:Neutrophil high* TMEs. In LUSC, we observed that TANs were associated with *PIK3CA* driver mutations that may contribute to or support immune escape. LUSCs with *PIK3CA* driver mutations more frequently underwent recent subclonal expansions. In addition, tumor cells with *PIK3CA* mutations upregulated *CXCL8*/IL-8, in accordance with previous work linking PI3K signaling with *CXCL8* expression in lung tumor and epithelial cells (66, 67). *PIK3CA* mutations have been linked to the regulation of glutamine metabolism (68) and nutrient consumption in proliferating tumors (69), in addition to proinflammatory cytokine expression (70). However, the proportion of Ki-67^+^ or MCT4^+^ tumor cells was not significantly different between *PIK3CA* mutant versus *PIK3CA* wild-type regions. Based on this, and their predominantly clonal status, we propose that these somatic alterations likely predispose the tumor to a neutrophil-rich TME and reveal a potential direct or indirect role for *PIK3CA* mutations in promoting tumor–neutrophil cross-talk in LUSC.

Collectively, these findings have therapeutic implications in early-stage resectable NSCLC, a population for which neoadjuvant immunotherapy plus chemotherapy is now a standard of care. Pathologic complete responses, which are associated with increased overall survival, following neoadjuvant treatment, range between 18% and 25% (71–74), highlighting the need to better characterize the TME and identify strategies to improve patient outcomes. Both *TS:TIL+M*Φ* high* and *T:TIL+M*Φ* excluded* TMEs were subject to a high degree of ITH that may affect checkpoint inhibitor (CPI) efficacy. T-cell–enriched spatial niches were predominantly found in the *TS:TIL+M*Φ* high* TME in LUAD and LUSC. These were, however, subject to selective pressures on neoantigen presentation through HLA LOH in LUAD and LUSC. These findings build upon previous work in NSCLC, which demonstrated an improved association of TMB with CPI responses after accounting for HLA LOH (75) and further highlight the importance of considering neoantigen clonality and the histologic subtype when designing neoantigen-mediated therapeutic strategies. In LUSC, *T:TIL+M*Φ* excluded* TMEs were associated with tumor cell PD-L1 expression and the clonal neoantigen-associated community of plasma and B cells. These results underscore the importance of investigating the predictive value of this low heterogeneity community of plasma cells and B cells in the context of CPI in LUSC and how spatial organization changes over time.

Although *TS:Immune low* and *TS:Neutrophil high* TMEs were subject to lower ITH, they may be less responsive to CPI. Dense, fibroblast networks may inherently restrict T-cell access and consequently CPI therapy response. Combinatorial targeting of CAFs and checkpoint molecules has seen success in preclinical models (76) and may prove beneficial in tumors with immune low TMEs for therapies designed with the increasing knowledge of CAF phenotypes and their functional heterogeneity (77). Finally, in neutrophil-enriched TMEs, CPI response may be limited due to increased levels of IL-8 expression, which has been linked to reduced benefit from CPI therapy based on both tumor and circulating IL-8 levels (78, 79). Nevertheless, clinical trials targeting neutrophils with anti-CXCR1/2 therapies and combinatorial CPI and anti-IL-8 are ongoing (NCT02536469; refs. 35, 80) and may provide an advantage in CPI treatment of neutrophil-high tumors. Of note, the patients in this study did not receive neoadjuvant or adjuvant CPI therapy. Future studies should seek to evaluate larger cohorts for both prognostic factors and predictors of CPI response in neoadjuvant and adjuvant settings and metastatic disease.

In conclusion, this study provides novel insights into the spatial organization of the early-stage lung cancer TME in the context of tumor immunogenicity, tumor heterogeneity, and cancer evolution. It highlights the importance of pairing the tumor evolutionary history with its spatially resolved TME to draw mechanistic hypotheses on tumor progression and metastasis with implications for patient outcome and treatment.

## METHODS

### Clinical Samples

The data from this study are part of the first 421 patients prospectively analyzed from the TRACERx cohort (https://clinicaltrials.gov/ct2/show/NCT01888601). The TRACERx study was approved by an independent research ethics committee (REC), the National Research Ethics Service (NRES) Committee London–Camden and Islington, with the sponsor's approval of the study by University College London (UCL) with the following details: REC reference 13/LO/1546, protocol number UCL/12/0279, IRAS project ID: 138871. Written informed consent for entry into the TRACERx study was mandatory and obtained from every patient. Methods for data obtention have been previously described (12, 14). Snap-frozen multiregion sampled tumor and adjacent normal tissues distant from the tumor within the resection specimen were processed to FFPE blocks after first taking sufficient material for DNA and RNA-seq. A single representative core was taken from each regional FFPE block (1.5 mm diameter) and arranged into TMAs representing 81 patients across eight blocks (Fig. 1A; Supplementary Fig. S1A). Control FFPE tissues for panel development were obtained from UCL/UCLH Biobank for Studying Health and Disease Renewal 2020 (ethics approval 20/YH/0088) and the PEACE study (ethics approval 13/LO/0972). Additionally, two 2-mm diameter cores were sampled from a tonsil FFPE block and assembled into a miniature TMA, which served as an internal staining control in this study.

### Clinical Data

The clinical data from this study have been previously described (12, 14). Growth patterns associated with regional LUAD tumor cores represent the predominant pattern in the corresponding regional block from which the core was derived (81). Cribriform, micropa­pillary, and solid patterns were grouped as high grade; lepidic and papillary as mid-grade; and acinar as low grade.

### IMC Panel Development

The pan-immune and T cells and stroma IMC panels were developed by testing antibody performance by immunofluorescence (IF) and IMC across NSCLC, normal lung, immune-rich tonsil, and immune-low cardiac and brain tissues (Supplementary Fig. S12A). H&E slides from FFPE tissue blocks used for panel development were assessed by a pathologist to identify cell types and features of interest to support staining evaluation and guide IMC scanning. Unconjugated antibodies were first evaluated using IF and then reevaluated following metal conjugation using IMC (Supplementary Fig. S12B). Metal-conjugated antibodies were purchased from Fluidigm, now Standard BioTools, and carrier-free antibodies (various suppliers) were conjugated to metals in-house using the Maxpar X8 Multimetal Labeling Kit (Standard BioTools). Antibody staining was evaluated for expected staining specificity, including costaining (e.g., CD20, CD79a), mutual exclusivity (e.g., CD4, CD8a), and signal-to-noise ratio, and was supported by pathologist evaluation (Supplementary Fig. S12C). The dilutions of all antibodies were derived by assessing a dilution series using IMC, with the experimental panel information applied to the TRACERx 100 cohort summarized in Supplementary Table S1. Examples of IMC staining across NSCLC, tonsil, cardiac, and brain tissue for the pan-immune and T cells and stroma panels are shown in Supplementary Fig. S12C. The results of this study focus on 38 antibody targets; some antibody results were not included as they were either not relevant to the biological conclusions of this study or due to technical performance.

### Tissue Processing for Immunofluorescence

FFPE tissue blocks were cut to 5 μm thickness. Sections were baked at 60°C in preparation for staining. Sections were dewaxed in xylene (2 × 5 minutes) and rehydrated in a graded series of alcohol (ethanol:deionized water 100:0, 100:0, 70:30, 0:100, 0:100, 1 minute each). Heat-mediated antigen retrieval was conducted in Tris-EDTA buffer at pH 9 for 30 minutes in a 900 W microwave. Tissue sections were slowly cooled in a room temperature water bath to prevent buffer crystallization. Sections were then washed in 1× PBS, and the tissue area was circled with a hydrophobic PAP pen to create a reagent barrier. Samples were dipped briefly in PBS-Tween20 (0.2%), before blocking with 1% BSA/PBS for 30 minutes at room temperature. Excess blocking solution was flicked off and the primary antibody/antibodies (diluted in 1% BSA/PBS) was applied and left overnight at 4°C. The next day, sections were washed three times in PBS (1 minute each), dipped briefly in PBS-Tween20, and the secondary antibody/antibodies was applied and left for 45 minutes at room temperature. Sections were washed three times in PBS (1 minute each), dipped briefly in PBS-Tween20, and counterstained with DAPI (1:5,000 in PBS) for 30 minutes at room temperature. Samples were washed three times in PBS (1 minute each) and immersed in 0.1% Sudan Black for 20 minutes at room temperature. Samples were thoroughly rinsed in cold running tap water until clear and rinsed twice in distilled water. Coverslips were mounted after adding 2 to 3 drops of VectaMount AQ aqueous mounting medium on the slides. Slides were stored protected from light until they were imaged using the Zeiss Axio Imager M1 system at 20x magnification.

### Tissue Processing for IMC

Several serial sections of each TRACERx TMA were cut to 5 μm thickness, and a 5 μm section from the tonsil TMA was floated onto the bottom of TMA002-TMA007 to serve as an internal staining control. Separate sections of tonsil were used as staining controls for TMA_REC and TMA001. All sections were floated onto standard positively charged slides for immunostaining. Two serial sections were selected and baked at 60°C in preparation for antibody staining; an additional serial section was subjected to H&E staining. Sections were processed as described for IF. The two metal-conjugated antibody cocktails (diluted in 1% BSA/PBS) were applied to serial sections and left overnight at 4°C. The next day, sections were washed three times in PBS (1 minute each), dipped briefly in PBS-Tween20, and counterstained with iridium (1:500 in PBS, Fluidigm) for 30 minutes at room temperature. Samples were washed three times in PBS (1 minute each) and counterstained with ruthenium (1:1,000) for 5 minutes on ice in a fumehood (82). Samples were washed three times in milliQ water (1 minute each) and left to air dry. Control tissues were processed following the same procedure during panel development.

### TRACERx IMC Data Acquisition

Data were acquired using a Hyperion Imaging System using commercial Standard Biotools IMC software (version 6.7). Regions of interest (ROI) selection was guided by pathologist review of a serial H&E section and were designed to capture each full TRACERx TMA core (1.5 mm diameter) or a 1.0 mm diameter tonsil ROI (Supplementary Fig. S12A). A laser ablated tissue ROIs in a rasterized pattern at 1 μm resolution and 200 Hz. The instrument was tuned between each run using a 3-Element Full Coverage Tuning Slide (Fluidigm, PN 201088).

### Spillover Compensation

To account for isotope signal spillover arising due to isotopic impurities, oxidation, and instrument properties (abundance sensitivity), we adapted the Chevrier and colleagues spillover compensation approach (83) for our two antibody panels. Briefly, data were generated using a slide spotted with lot-specific metal-conjugated antibodies. Superfrost Plus histology slides (Thermo Fisher) were heated on a heat block and coated with a 2% agarose film, ensuring an even spread and thin layer (UltraPure Agarose, Thermo Fisher). The slides were then left to dry until the agarose fully solidified. Metal-conjugated antibodies were mixed 1:1 with Trypan blue and spotted on the slide serially, ensuring no merging of spots into each other. Once the spots had dried, an ROI was chosen for each separate spot on the Hyperion (Fluidigm), corresponding to one metal-conjugated antibody. Each ROI was 10 pixels high by 200 pixels wide and was ablated at a laser power 2 units higher than the tuning laser power.

From this experimental spillover data, we calculated compensation matrices using the scripts from Chevrier and colleagues (83), with minor adaptations made to the code. To address observed median pixel-level ion counts that were lower than a threshold required to achieve an accurate readout of metal isotope impurities (threshold = 250 counts), for each metal–antibody conjugate ROI in turn, we implemented an adaptive binning approach in which pixel count data are automatically and progressively aggregated over adjacent pixels until the count threshold is reached, having also first restricted analysis to the 50% of pixels with the highest ion counts within the spot (Code and Data Availability). Experimental spillover matrices from the pan-immune panel and the T cells and stroma panel were highly similar to the matrix reported by Chevrier and colleagues and channel spillover ranged from 0 to 4.1% (Supplementary Fig. S13A and S13B).

### Cell Segmentation

To segment nuclei, we trained a UNet++ deep-learning model on a large manually labeled data set developed in-house (*n* = 116 images, 42,000 nuclei; see Data availability) to predict three semantic features of nuclei from ground truth data: nucleus center of mass, all nuclear material, and nuclear boundaries, which we then combined via a marker controlled watershed procedure into nucleus label masks. These nuclear masks were then fed into a multiplexed imaging–specific whole-cell segmentation procedure, which uses the many independent cell marker channels available in IMC to produce final cell masks (Supplementary Fig. S14A and S14B).

In brief, we segmented whole cells by first generating a series of independent cell masks, one for each of a set of user-defined cell lineage markers. To achieve this, deep-learning nuclei were associated with each lineage marker in turn, using a minimum overlap criterion applied between the nuclei and a lineage marker mask created by Otsu-thresholding a preprocessed lineage marker image. Preprocessing steps for lineage marker channels were hot pixel removal and median filtering (window size = 3 px). Nuclei associated with an Otsu-thresholded lineage mask were then used as the seeds for lineage marker-specific cell label generation using propagation-based secondary object identification onto the relevant minimally preprocessed lineage marker image. This step yielded a set of instance-level lineage marker cell labels which were then combined into a consensus set of whole-cell labels using a serial masking approach. In this step, only pixels with consensus between different lineage marker channels were retained in the same single-cell object, the aim of which was to minimize segmentation artifacts where mutually exclusive markers are found in the same cell.

We observed extensive nonnucleated and elongated αSMA content in our T cells and stroma panel, putatively fibroblasts, and to account for this in our segmentation, we implemented an additional step to identify these αSMA^+^ stromal cells without an in-plane nucleus for this panel only. To do this, we performed primary object identification directly on the minimally preprocessed αSMA channel, following qualitative optimization of thresholding parameters on a representative subset of cores. We filtered nonnucleated cells identified with this method to ensure overlap with nucleated cell objects was minimal (less than ⅓ of the area of any nonnucleated cell) and that each identified nonnucleated cell had an area of at least 20 pixels following masking by the nucleated cell mask. These steps were implemented to reduce the likelihood of double counting cells and of assignment of cell debris or artifacts to cell area. The nonnucleated αSMA identification step contributed a median of 560 cells per tumor core (median 5.6% of total cells/core).

The whole-cell segmentation procedure was implemented using CellProfiler v3.1.9 (84). Single-cell measurements of all IMC marker mean intensities were input into the TYPEx multiplexed cell phenotyping module.

### Cell Phenotyping

Cell subtypes were identified and quantified in three steps: iterative cell stratification, statistical comparison of marker intensities, and cell subtype assignment. The cells were stratified into groups with similar marker intensities as follows: (i) cells were assigned to the most likely major cell lineage using CellAssign (85) accounting for TMA ID as a potential batch effect; (ii) low and high confidence assignments were identified as those that changed label by perturbing the input to the CellAssign model and excluding one cell lineage. Myeloid cells–other (CD11b) were excluded for the pan-immune panel and Vim^+^ cells (Vimentin) for the T cells and stroma panel; and (iii) clustering with FastPG (biorxiv 2020.06.19.159749v2) was performed within a major cell lineage and confidence group, where the parameter k was set to 30, and no transformation was applied to the raw mean pixel intensities per cell prior to clustering. The clusters were compared by the pixel intensities of all markers of interest in the panel, and the probability distribution for a cluster to have a higher intensity than other clusters was determined. If the probability distribution was higher than the background distribution of all clusters, the marker was considered positive. This separation ensured that rare or unexpected T-cell populations such as CD3^−^CD8a^+^, CD3^−^CD4^+^CD8a^+^, CD3^+^CD4^+^CD8a^+^ were minimized relative to the common T-cell populations, CD3^+^CD4^+^ and CD3^+^CD8a^+^. Cell subtypes were assigned automatically based on the combination of positive markers, given the cell phenotype definitions in Fig. 1E; Supplementary Fig. S2B, and Supplementary Fig. S3A.

The major cell lineage markers used to build the CellAssign model for the pan-immune panel were endothelial cells (CD31), epithelial cells (pancytokeratin/panCK), CD4 T cells (CD45, CD3, CD4), CD8 T cells (CD45, CD3, CD8a), T cells–other (CD45, CD3), B cells (CD45, CD79a, CD20), monocytes (CD45, CD11b, CD14), macrophages (CD45, CD11b, CD14, CD68, CD206, CD163), myeloid cells–other (CD45, CD11b), mDCs (CD45, CLEC9a), and leukocytes–other (CD45). For the T cells and stroma panel, the following major cell lineages were defined: Vim^+^ cells (Vimentin), endothelial cells (Vimentin, CD31), epithelial cells (panCK), aSMA^+^ cells (Vimentin, aSMA), CD4 T cells (CD45, CD3, CD4), CD8 T cells (CD45, CD3, CD8a), T cells–other (CD45, CD3), leukocytes–other (CD45).

To examine potential batch effects arising from staining multiple slides, we compared the raw intensities across tumor cores for all TMAs for both the pan-immune and T cells and stroma panels (Supplementary Fig. S15A and s15B). We did not observe batch effects in this analysis nor in UMAP representations when data were grouped by stained TMA section (Supplementary Fig. S15C and S15D). In addition, we compared the median marker intensities across all the cells per image between the two antibody panels (Supplementary Fig. S16A and S16B). Immune, endothelial, epithelial, and total cell densities were highly correlated between the two antibody panels, with Spearman correlation coefficients of 0.72 for total cells, 0.9 for total T cells, 0.85 for epithelial cells, and 0.82 for endothelial cells.

### Cell Subtype Definitions

The following cell type definitions were applied to define cell subtypes. Macrophage subtypes were defined as follows: CD163^+^CD206^+^ macrophages (CD68^+^CD206^+^CD163^+^) and CD163^−^ macrophages (CD68^+^CD206^+/−^ alone). CD163^+^CD206^+^ macrophages that were located within pathologist-annotated masks of alveolar macrophages were classified as alveolar macrophages (Supplementary Fig. S2C).

Neutrophils were defined as CD11b^+^MPO^+^ cells negative for other cell subtype-specific markers, such as CD14 (monocytes) and CD68/CD163/CD206 (macrophages). Additional cell subtypes were defined as naïve T cells (CD45RA^+^), cytotoxic T cells (GZMB^+^), tissue-resident memory T cells (CD103^+^), exhausted terminally differentiated T cells (CD57^+^CD39^+^CD103^+/−^), regulatory T cells (Treg; FOXP3^+^), central memory T cells (Tcm; CD27^+/−^CCR7^+^), effector memory T cells (Tem; CD27^+^CCR7^−^), CD57^+^ T cells (CD57^+^), plasma cells (CD79a^+^CD38^+^), gamma-delta T cells (Tgd; CD3^+^TCRd^+^), B cells (CD79a^+^CD20^+^), B cell lineage–other (CD79a^+^CD20^−^CD38^−^), and a myeloid cells–other subtype (CD11b^+^MPO^−^CD14^−^CD68*^−^*).

αSMA^+^ cells with cell centers falling within large vessel mask areas, as annotated during pathology review, were assigned as perivascular αSMA^+^ cells. Remaining αSMA^+^ cells in images were assigned as αSMA^+^ fibroblasts (Supplementary Fig. S2C).

A proportion of unassigned cells in the pan-immune data set are likely fibroblasts or other stromal cells, for which this IMC panel did not include markers specific for these cell types. A proportion of Vim^+^ cells in the T cells and stroma panel data is attributed to the myeloid lineage, which was not readily identified in this panel.

In all analyses using the proportion of tumor cells, we have quantified the proportion of positive tumor cells out of all tumor cells, defined as the epithelial cells within pathology expert-annotated tumor areas (Supplementary Fig. S2C).

### Tissue Segmentation of Tumor Nest and Stroma

A three-class random forest classifier was trained to segment background, tumor/epithelium, and stroma tissue regions on composite images from NSCLC tumor, tumor-adjacent lung tissue, and lymph nodes using Ilastik (86). The composite image was generated using DNA intercalators and markers with tissue-specific expression for the epithelium (pancytokeratin) and stroma (immune-specific biomarkers, αSMA, CD31). When available, vimentin (T cells and stroma panel), collagen1 (T cells and stroma panel), and panactin (pan-immune panel) were also considered in areas with mutual exclusivity with the epithelial cell markers. Of note, the tissue area that was not stained by any of the markers in the antibody panel, such as air space, was detected as background. The performance of this classifier was validated through pathology review of paired H&E images.

To account for differences in the imaged tissue area, we used cell density, i.e., the cell count normalized by the imaged tissue area. The imaged tissue area was calculated as the sum of the areas of the tissue compartments, TS, derived from tissue segmentation. Median cell densities are summarized in Supplementary Table S4.

### Pathology Review and Feature Mask Generation

For each imaged ROI, a serial H&E was reviewed by an expert pathologist to confirm the presence or absence of invasive tumor tissue and to annotate tumor and nontumor epithelium, airways, necrosis, large vessels, and alveolar macrophages (Supplementary Fig. S17). Annotations were made using NDP.view2 software (version 2.7) on a pseudo-H&E generated directly from the ruthenium and iridium channels of our IMC images. Masks for each labeled feature were created using Groovy scripting in QuPath and aligned with study outputs.

H&E images from the regional FFPE tissue block from which TMA cores were derived were assessed for the presence of TLS. TLS were defined as lymphoid aggregates with the presence of segregated T-cell and B cell areas, as well as evidence of an ongoing GC reaction, based on the distinction of dark and light zones in GCs. There was no evidence for TLS associated with the tumor regions assessed by IMC in this study.

### Identification of Spatial Cellular Communities

The community identification method (87) was applied to 139 tumor cores that were imaged with the pan-immune panel to identify groups of cells that commonly localized near one another. Cell subtypes included in the analysis had a minimum average of 10 cells per core in all tumor cores. Unassigned and ambiguous cells were excluded from community analysis. A window was defined around every cell in an image and its 10 nearest neighboring cells including the center cell. These windows were clustered by their composition with respect to the 18 cell types (with at least 10 cells on average per image) using MiniBatchKMeans and k = 10. To identify cell types enriched in a community, we calculated if the density of a cell type was significantly higher in the community of interest compared with all other communities using an LME model with patient as a random effect and ANOVA test. Communities were then assigned representative names based on the enriched cell types within them (Supplementary Fig. S3F). The community identities were mapped onto segmented cells and visualized using Cytomapper (Supplementary Fig. S3G; ref. 88), which were then validated by pathologist assessment of serial H&E-stained tissue sections.

To check that the communities we detected in the TME were robust, we performed a series of tests to validate our results. To find the optimal *k* number of communities, we varied the number of communities and measured the stability of constituent cell types within them. For each window size, we tested *k* = 1 to 20 communities and calculated the distortion score, representing the sum of squared distances from each point to its assigned center. Using the elbow locator, we found that the optimal number of points that maximized the decrease in distortion was *k* = 10 communities (Supplementary Fig. S18A). We investigated the differences in output by testing window sizes of *n =* 2, 5, 10, 15, 20 nearest neighbors. We observed that the composition of the resultant communities was largely unchanged, in that they were enriched for similar cell types (results not shown). Robustness of clustering was tested by subsampling one third of the cells three times and comparing the proportion of cells assigned to the same community with each iteration of clustering, resulting in a median concordance of 80% (Supplementary Fig. S18B).

All 10 communities were detected across NSCLC histologic subtypes, with no statistical enrichment in LUAD, LUSC, or NSCLC-Other (Supplementary Fig. S18C).

### Spatial Clustering

We performed spatial clustering of tumor cells in pathologist-annotated tumor areas as fiducial structures for spatial analysis (Supplementary Fig. S2D). Spatial clustering of cell coordinate data were performed using the DBSCAN algorithm from the Python library scikit-learn. The eps parameter of the DBSCAN algorithm was set to 25, resulting in reasonable cell clusters by visual assessment. We used a minimum cluster size of 3 cells for spatial analysis. We used the Python packages Alphashape and Shapely to determine the boundaries of all spatial clusters and whether any cell was located within a given spatial cluster.

### Barrier Score Definition

αSMA^+^ fibroblast barrier scores were adapted from the method of Failmezger and colleagues (89) and calculated as follows. First, a nearest neighbor graph of cell locations was constructed by connecting each cell to its five nearest neighbors. To calculate the αSMA^+^ fibroblast barrier for CD8 T cells with respect to tumor cells, we used the breadth-first search shortest paths algorithm from the Python cuGraph library to find the shortest paths from each CD8 T-cell vertex in the network to all tumor cell vertices. αSMA^+^ fibroblasts were then enumerated along each path. For originating CD8 T cells which had multiple tumor cells at the same distance (e.g., two tumor cells each at a five-hop distance from the CD8 T cells), the score was defined as the average number of αSMA^+^ cells across all paths. To restrict the scoring to αSMA^+^ cells specifically accruing at the edge of tumor bulk, we counted only αSMA^+^ cells adjacent to tumor cells (i.e., neighboring nodes of the cell spatial graph) and only if the tumor cell itself was a member of a spatial cluster of at least 2,000 μm^2^ as defined by DBSCAN, described above. We chose these parameters as the concept of a macroscopic barrier may be poorly defined for single and small numbers of tumor cells. Furthermore, to focus analyses on nonperivascular cell populations, including nonperivascular αSMA^+^ cells, cells with centers falling within pathologist-annotated large vessel masks were annotated as perivascular and excluded from measurement. Nonperivascular epithelial cells with centers captured within the pathologist-annotated tumor mask area were assigned as tumor cells. Barrier scores were calculated as the per-image mean across all nonperivascular CD8 T cells, where a barrier score of 1 is assigned for a CD8 T-cell separated from the nearest tumor cell by a tumor cell–adjacent αSMA^+^ fibroblast (0 otherwise) and where the barrier score per CD8 T cell is calculated as the mean score over all shortest paths to the nearest tumor cell(s).

### TME Classes Definition

Unsupervised hierarchical clustering of z-score normalized cell densities was performed using all cores in the study, including normal, benign tumor–adjacent, and tumor samples of all histologic subtypes. The following major cell types were used to define the TME clusters: CD8 T cells, CD4 T cells and B-cell lineage (TIL), CD163^+^CD206^+^ macrophages and CD163^−^ macrophages (Mφ), neutrophils and myeloid cells–other (mDC, monocytes, other CD11b^+^ cells). The cell densities for each major cell type were calculated individually for the TS. Cells from the B-cell lineage were predominantly found in one tissue compartment, stroma (98%). Only a small number of B-cell lineage cells and myeloid cells–other, fewer than 3,000 cells or 0.3% from all immune cells, were detected in the tumor nest; therefore, the cell densities in the tumor nest for these two cell types were not considered for TME classification. All analyzed cell types were detected in at least 89% of the analyzed cores.

Four TME clusters were defined as the most concordant clusters derived from hierarchical clustering using 75% of the samples in 1,000 subsampling iterations, using the functions ConsensusClusterPlus and calcICL within the ConsensusClusterPlus R package (v 1.58; Supplementary Fig. S5A). The clustering was performed on normalized cell densities using robust z-scores (median divided by median absolute deviation). Clustering was performed using the distance metric maximum and the clustering method ward.D. The cumulative distribution function (CDF) of the consensus matrix for each value of *k*, where *k* is the number of clusters, and the difference in area under the curve comparing the CDF for *k* with the CDF for *k* − 1 were included in Supplementary Fig. S5B. The consensus value indicates the proportion of instances that two cores are assigned to the same cluster out of 1,000 subsampling iterations. We demonstrated that the largest increase in consensus values was observed by increasing the number of clusters from three to four (Supplementary Fig. S5B). This analysis strongly suggests that there are at least four TME classes in the cohort. The consensus values continue to increase with *k* larger than 4 although with a smaller difference, suggesting that the spatial TME classification may be refined in significantly larger cohorts. Five TME clusters resulted in splitting the *TS:Neutrophil high* TME class predominantly by histology-specific differences and CD163^−^ macrophage densities in the tumor nest yielding a predominantly LUSC cluster with a significantly higher density of neutrophils in the TS than for other TME clusters combined and a predominantly LUAD cluster with a higher density of neutrophils and CD163^−^ macrophages in the tumor nest compared with other TME clusters. Because of the high similarity of these two clusters, we considered four TME classes in this study.

We next identified the criteria that distinguish each cluster, using an LME model to compare cores from a given immune cluster to those from other clusters and adjust for multiple regions with patient as a random effect (Fig. 2D). Specifically, *TS:TIL+M*Φ* high* cores were selected for cores with higher TIL or macrophage densities in the tumor nest compared with other TME classes. *TS:Immune low* cores were selected to have lower immune cell density in the tumor nest compared with cores from other TME classes. The criteria for the *T:TIL+M*Φ* excluded* TME required that TIL/macrophage density in the tumor nest was lower than *TS:TIL+M*Φ* high* and that TIL/macrophage density in the stroma was higher than in *TS:Immune low* cores. Finally, as the *TS:Neutrophil high* cluster was characterized by higher neutrophil infiltration in tumor nest or stroma and lower TIL infiltration, the criteria for this TME class required that the neutrophil proportion from all cells was higher than other TME classes in the tumor nest or stroma. The cutoffs for each of these criteria were determined automatically using a binomial generalized linear model for each immune cluster compared with other clusters and are shown in Supplementary Fig. S5C. The performance function in the R package *ROCR v.1.0-11* was used to find the best cutoff as the intersection between the sensitivity and specificity curves. Within each cluster, the cores that did not fulfill these criteria were labeled *undefined* (Supplementary Fig. S5A and S5C).

The TME classes were annotated based on cell types with differential composition in the tumor nest or stroma compartment compared between each TME class versus other TME classes, using an LME model with patient as a random effect (Fig. 2D; Supplementary Fig. S5D). For NSCLC analysis, histology was added as a fixed effect (Fig. 2D). Histology-specific median cell densities are summarized per TME class in Supplementary Table S4.

### Spatial Heterogeneity of TME Classes and TAN-High Tumors

To evaluate the intratumor spatial heterogeneity of TME classes accounting for the different number of sampled regions, we performed bootstrap subsampling with 1,000 iterations. We calculated the probability of observing a TME class for a given number of samples taken per tumor (two, three, and four samples). The heterogeneity of a given TME class was estimated as the ratio of the number of observations when all samples had that same TME to the total number of tumors that have that TME class. This reported probability of observing the same TME class across all samples represents the average ratio across all iterations and across the different number of samples taken (two to four). This approach was used to estimate the probability of all regions to have a high TAN score and the probability for any region to have a high TAN score given a clonal *PIK3CA* mutation in the TRACERx 421 (Tx421) cohort, varying the number of samples from two to eight (the maximum number of regions in the Tx421 cohort).

### Spatial Heterogeneity of Cell Types and Communities

A spatial ITH score for cell type and community densities was calculated using z-score normalized density values in patients for which data were available from two or more tumor cores (pan-immune panel *n* = 41, T cells and stroma panel *n* = 39; Supplementary Fig. S1B). The standard deviation was calculated across tumor core data per patient. The spatial ITH score per feature represented the cohort mean of standard deviation values, per IMC panel.

### PD-L1 IHC of Regional Tumor Blocks

FFPE sections (4 μm) were stained with anti–PD-L1 (SP142) according to the manufacturer's instructions (Ventana). Tumor cell (TC) scoring was performed as instructed by the manufacturer by experienced and qualified pathologists. Briefly, the TC score was defined as the proportion of viable tumor cells showing PD-L1 membranous staining of any intensity. TC scores were categorized as <1%, 1% to 49%, and ≥50%. For statistical analysis, TC scores ≥50% were rare and were therefore combined with 1% to 49% into a ≥1% category. Samples stated as negative for PD-L1 had a staining positivity rate of <1%.

### IHC Validation of Checkpoint Molecule Expression

Multiplexed IHC was performed to validate immune-checkpoint molecule expression on immune cells. FFPE tissue sections of a representative human lung cancer case and of reactive tonsils were subjected to double immunostaining (Supplementary Fig. S19). Briefly, 2- to 5-μm tissue sections were cut and transferred on electrically charged slides to be stained. To establish optimal staining conditions (i.e., antibody dilution and incubation time, antigen retrieval protocols, suitable chromogen), each antibody was tested and optimized on sections of reactive tonsil by conventional single IHC using the automated platform Bond-III Autostainer (Leica Microsystems). For double immunostaining, a protocol previously described was carried out (90). All slides were stained with anti-MUM-1/IRF4 clone MUM1p (1:400, Agilent Dako) and costained with one of anti–PD-L1 clone SP142 (Ventana Medical Systems), anti-TIM3 clone D5D5R (1:100, Cell Signaling Technology Inc.), anti-VISTA clone CL3975 (1:150, Thermo Fisher Scientific Inc.). Slides were counterstained with hematoxylin. Images were acquired on a NanoZoomer 2.0HT whole-slide imaging system (Hamamatsu Photonics) at 40× magnification.

### TAN Scoring from H&E Images in TRACERx

The densities of TANs were assessed on digitally scanned H&E-stained 3 μm sections and scored at 400× magnification. All TANs were evaluated within the histologic limits of the tumor itself. The TANs in the stromal compartment and tumor island compartment were evaluated separately. Calculation of the TANs in the stromal compartment was based on the standardized method used for TIL quantification, as developed by the International Immuno-Oncology Biomarker Working Group on Breast Cancer (30). The tumoral TANs were scored in a similar manner but the areas assessed included viable tumor islands, and neutrophils free floating within the glandular lumen were excluded. As per the published guidelines, the percentage was calculated as the area occupied by the neutrophils over the total stromal area and total tumoral area, respectively. Stromal TANs (sTAN) represent the percentage of stroma compartment area occupied by the TANs; tumoral TANs (tTAN) is the percentage of tumor compartment area occupied by the TANs. The average neutrophils scoring was calculated across the entire slide rather than focusing on the hotspot areas. The percentage is given as a continuous parameter. Eighteen patients were excluded from the validation cohort and were not scored for TANs when H&E slides were not available or no tumor was detected in those sections. Region-level TAN scores were derived from scoring H&E images of paired regional blocks of the study TMAs, and tumor-level TAN scores were assessed on tumor-matched diagnostic blocks.

High TAN score was defined based on whether either the tumor (tTAN) or the stromal TAN (sTAN) scores were high. To classify tTAN and sTAN scores into high and low, we determined an optimal cutoff that best separates the *TS:Neutrophil high* TME class from the other TME classes. We used a binomial generalized linear model of the tTAN or sTAN score to predict the presence of a *TS:Neutrophil high* TME class. We used the *performance* function in the R package ROCR v.1.0-11 to find the best cutoff as the intersection between the sensitivity and specificity curves. The determined cutoffs were 2%/3% for tTAN and 1%/2% for sTAN across all diagnostic slides scores in LUAD/LUSC, and 1%/5% for tTAN and 1%/1% for sTAN across regional TAN scores in LUAD/LUSC.

### RNAscope

FFPE sections (5 μm) were stained on the Leica Bond Rx automated stainer using RNAscope LS Multiplex Fluorescent assay (322800 ACD Bio-Techne) applying a standard 15-minute target retrieval and 15-minute protease treatment using target probe Hs-IL-8 (310388 Bio-Techne) with Opal 570 (Akoya Biosciences). Samples were immunostained with MPO 1:2,000 (ab208670 Abcam) or pan-cytokeratin 1:250 (M3515 Agilent) and detected with Opal 690 (Akoya Biosciences) and counterstained with DAPI. Slides were imaged on the PhenoImager HT (Akoya Biosciences).

### Neighborhood Analysis

The CellProfiler software (v3.1.9; MeasureObjectNeighbors module) was used to compute the neighboring cells for each cell type in an image (84). Cells were defined as being in the “neighborhood” of a cell of interest if they were detected within a pixel radius of 5 px of the cell of interest.

The neighbouRhood tool (91) aggregate_classic function was used to define the image-level cell–cell relationship between any two cell types: interacting relationship, avoiding relationship or nonsignificant relationship.

*TME class comparisons, PIK3CAmut/wt, and subclonal expansion score high/low:* To query if a relationship was significantly enriched in one subgroup compared with another, a logistic regression model correcting for multiple cores per tumor was applied to assess the frequency of a spatial relationship in the TME class of interest compared with all other TME classes (Supplementary Fig. S20), between tumor cores with *PIK3CA* mutation versus *PIK3CA* wild-type (Supplementary Fig. S21A) and between cores with high compared with low subclonal expansion scores (Supplementary Fig. S21B). Benjamini–Hochberg adjustment was performed to correct for multiple testing. We report significant (*P*_adj_ < 0.05) cell–cell relationships if the constituent cell types were present in at least 90% of tumor cores. These relationships were visualized in a heat map if they were present in at least 10% of cores.

*MCT4^+/−^ and CAIX^+/−^ TC neighborhood analyses:* Differences in interaction and avoidance profiles with other cell subtypes in *TS:Neutrophil high* LUSC tumor cores were analyzed between center MCT4^+^ and MCT4^−^ tumor cells (Supplementary Fig. S22A), and between center CAIX^+^ and CAIX^−^ tumor cells (Supplementary Fig. S22B). We report cell–cell relationships if the constituent cell types were present in at least 90% of tumor cores (≥14 cores) for both positive (+ve) and negative (−ve) center tumor cell phenotypes. No significant differences were observed between +ve and −ve center cells for either MCT4 or CAIX, when applying a Chi-square test and *P* value adjustment (Benjamini–Hochberg). Only cell subtypes for which at least two cores exhibited a significant avoidance or interaction for at least one of the two tumor phenotypes were tested.

### TRACERx 100 WES

WES data were available for 98% of tumor cores in the pan-immune data set, and for 98% of tumor cores in the T cells and stroma data set (Supplementary Fig. S1A; Supplementary Table S2). Normal lung samples were not subjected to WES. WES data were processed as described (14).

### TRACERx 100 RNA-sequencing

RNA-sequencing data were processed as described (13). Paired analysis of RNA-sequencing and IMC data were performed for tumor cores only and was available for 78% of the pan-immune and 76% of the T cells and stroma data sets (Supplementary Fig. S1A; Supplementary Table S2).

### TMB Calculation

TMB was calculated using a harmonized approach as the number of somatic mutations per megabase (muts/Mb) in the coding genomic regions (92) on the TRACERx 421 mutation data from WES. The TMB status of each tumor region was categorized as either high (≥10 mutations/Mb) or low (<10 mutations/Mb) to match clinical guidelines.

### Driver Mutation Analyses

Driver mutation calls are derived from TRACERx 421 driver mutation calling from WES, as described previously (14), and include driver single-nucleotide variants, insertion–deletion mutations (indels), and splice mutations in relevant genes. Clonality calls for *PIK3CA* mutations were derived using TRACERx clonality calling for WES data, as described in (14).

Statistical analyses of TME class distributions by regional driver gene mutation status were performed using the lme4 R package glmer function (“binomial” distribution). Analyses were undertaken to compare distributions of one TME class with all other classes combined (for the respective histology subset). Patient ID was included as a random effect, and ANOVA *P* values were calculated by comparing a null model without mutation status as a fixed effect to a model containing mutation status as a fixed effect.

Nearly all the observed driver mutations in the PI3K pathway occurred in the gene *PIK3CA* in TRACERx 421 (23/115 cores). There were no *PIK3CB* mutations in TRACERx 100. Gains and amplifications in regions of the *PIK3CA* gene were a frequent event in nearly all LUSC tumors.

Chi-squared test was used to compare the frequency of observations between *PIK3CA* mutant and *PIK3CA* wild-type tumors. In the case of multiple regions per tumor, we considered whether any tumor region of a given tumor had a mutation.

### Copy-Number Analysis

Somatic copy-number profiles for tumor regions were derived as previously described (14). To compare somatic copy-number alteration (SCNA) profiles in high and low TAN score regions, the previously described unpaired analysis method was adapted (15). Within each of high and low TAN categories separately, for each copy-number segment within an individual tumor, the maximum and minimum log_2_ copy-number values from all respectively assigned regions of a tumor were selected. GISTIC2.0 (93) was then run four times for each of LUAD and LUSC for the combined TAN validation and discovery cohorts using these tumor-level data, once with the maximum values (to examine amplifications) and once with the minimum values (to examine losses), for each of high and low TAN score categories. Driver or immune evasion genes deemed to be significantly amplified/deleted (q<0.1) for the TAN-High group but nonsignificant for the same SCNA event in the TAN-Low group, and for which the absolute value of the G-score was higher in the TAN-high compared with the TAN-low group were assessed. No such peaks were observed in LUAD or LUSC. In this instance, driver genes were defined as in (14, 15). Immune evasion genes tested were *RFX5*, *RFXANK*, *RFXAP*, *TAP1*, *TAP2*, *TAPBP*, *PSMB8*, *PSMB9*, *NLRC5*, *ERAP1*, *CALR*, *CNX*, *PDIA3*, *B2M*, *SPPL3*, *MOGS*, *GANAB*, *CIITA*, *MARCHF1*, *CD74*, *MARCHF8*, *CGAS*, *MB21D1*, *TMEM173*, *TBK1*, *IRF3*, *IFNB1*, *CTNNB1*, *AXIN1*, *AXIN2*, *APC*, *GSK3*, *GSK3B*, *CSNK1A*, *CSNK1A1*, *DKK1*, *PTCH1*, *NKD1*, *PTEN*, *MYC*, *PTGS2*, *CXCL13*, *CXCL9*, *IFNGR1*, *IFNGR2*, *JAK1*, *JAK2*, *STAT1*, *STAT2*, *IRF1*, *IRF9*, *SOCS1*, *IFNAR1*, *IFNAR2*, *SERPINB9*, *SERPINB4*, *FAS*, *CFLAR*, *TNFRSF10A*, *TNFRSF10B*, *TNRFSF10C*, *TNRFSF10D*, *TGFB1*, *TGFB2*, *TGFB3*, *CD274*, *IDO1*, and *PDCD1LG2*.

### Class I/APM Disruption

Class I/APM disruption refers to intrinsic mechanisms of immune escape relating to antigen presentation on MHC class I. Class I/APM disruption was defined if HLA LOH could be determined in any of *HLA-A*, *HLA-B*, or *HLA-C*, and/or if mutations were detected in any of the following antigen presentation (APM) genes: *B2M*, *HLA-A*, *HLA-B, HLA-C, CALR, ERAP1, GANAB, MOGS, NLRC5, PSMB8, PSMB9, PDIA3, RFX5, RFXANK, RFXAP, SPPL3, TAP1, TAP2, TAPBP*, and *CNX*. Mutations were defined as any of nonsynonymous single-nucleotide variants (including stoploss and stopgain), and (non-)frameshift insertions and substitutions, and frameshift deletions. To be included in the HLA analysis, a gene had to pass the following filters: identified at least 10 SNPs that passed the minimum coverage of 30; both alleles of the gene required an expected depth (ED) of ≥10; the 95% confidence interval in the allelic copy number was <2.5. The ED estimates the depth of the reads sourced from the cancer cells, which was calculated from the depth of the matched germline sample and the purity of the tumor region. At an ED below 10, we did not expect to have the required coverage to accurately classify LOH, even if it were present.

### Whole-Genome Doubling

Whole-genome doubling assignments for TRACERx tumor regions, referenced in Fig. 2B, were calculated as described previously (14).

### Neoantigen Analysis

Neoantigen analysis was performed within LUAD and LUSC histologies and required samples with neoantigen prediction and RNA-seq. Clonal (detected in all regions of the same tumor), subclonal (not detected in all regions of the same tumor), and total nonsynonymous mutations were used to predict neoantigens using NetMHCpan4.1 (94). When bulk transcriptomic data were available, a neoantigen was considered to be expressed if at least four RNA-seq reads mapped to the mutation position. Neoantigen counts were further filtered based on whether they were predicted to bind to the corresponding patient's HLA alleles (determined using HLA-HD; ref. 95) that were not subject to HLA LOH. The HLA-binding predictions were filtered based on strong binding affinity using a threshold of rank score <0.5%. LOH status of *HLA-A*, *HLA-B*, and *HLA-C* could not always be determined (see above). Tumor regions with no data for any of *HLA-A*, *HLA-B*, or *HLA-C* were excluded from neoantigen analysis requiring HLA status and represented 3 LUAD tumor regions from 2 patients and 3 LUSC tumor regions from 1 patient (Supplementary Fig. S1A; Supplementary Table S2). In tumor regions where LOH of one or two of *HLA-A*, *HLA-B*, or *HLA-C* was undetermined, the missing HLA was assumed to be intact. The expression level of neoantigen transcripts and RNA-level repression of HLA genes were not considered.

### Differential Gene Expression, Gene Set Enrichment, and GO Analyses

Trimmed mean of M-values (TMM) normalized expression values was analyzed with the limma–voom workflow for differential analysis (13). The t-statistic generated by limma was used as input for GSEA for MSigDB hallmark gene sets using the R package fgsea (v1.10.1) with default parameters. Genes with higher than 2-fold change and limma-derived unadjusted *P* < 0.05 were selected for overrepresentation analysis with GO Biological Processes using the enrichGO function in the clusterProfiler R package (v 4.2.2).

### Recent Subclonal Expansion Score

A recent subclonal expansion score per tumor, reflecting the size of the largest recent subclonal expansion within each tumor region, was calculated as described by Frankell and colleagues (14). In short, using multiregional WES, tumor phylogenetic trees were constructed, and for each of them, the terminal nodes on the tree (i.e., leaf nodes) were identified. The maximum cancer cell fraction (CCF) of any of these leaf nodes was then identified. The recent subclonal expansion score represents the maximum CCF of any of the leaf nodes in a given tumor region. The recent subclonal expansion score was compared between the TAN groups using the LME model with patient as a random-effect covariate. To adjust for the difference in tumor content, we added purity as a fixed-effect covariate in the model. The association of high recent subclonal expansion with TAN-high tumor cores remained significant in LUAD (*P* = 0.047) but did not reach significance in LUSC (*P* = 0.26).

### Phylogenetic Tree and cloneMap Visualization

The tumor phylogenetic tree for tumor CRUK0468 in Fig. 6F was reconstructed using CONIPHER (14, 96) and visualized using the plot function (igraph R package v1.3.5). The sequenced profiles of two samples from a lymph node metastasis, one fresh frozen (LN1) and one FFPE (FLN1), were included with the primary tumor regions to reconstruct the tumor phylogenetic tree. Tumor region clone maps were visualized using the cloneMap function (cloneMap R package v1.0.0.0, bioRxiv 2022.07.26.501523).

### Survival Analysis

To evaluate the prognostic value of TANs in lung cancer, we defined a discovery cohort as the TRACERx100 tumors profiled with IMC for neutrophil markers (panel 2, *n* = 68) and a validation cohort as the nonoverlapping TRACERx421 tumors with a surrogate, H&E-derived TAN score (*n* = 332; Supplementary Fig. S10A). In eight cases with TAN scoring, when the patients harbored synchronous multiple primary lung cancers, we used only data from the tumor of the highest pathologic TNM stage. We excluded two patients for which multiregion sequencing data revealed two tumor masses as collision tumors with two and three independent LUADs in CRUK0881 and CRUK0704, respectively, diagnosed histologically as single primary LUADs (14). One patient (CRUK0682) with synchronous primary lung cancers (LUAD and LUSC), whose tumor with the highest stage (LUAD) was not sequenced, was excluded from the survival analysis.

Within the discovery cohort, we distinguished tumors with metastasis-seeding clones (*n* = 22) as metastasizing tumors, as determined by multiregional genomic profiles from matched primary and metastases (15). All tumors seeding lymph node and intrapulmonary metastasis detectable at the time of surgery (*n* = 12) as well as any relapse detected during follow-up were included (*n* = 10). In addition, we also defined control tumors as those from patients who did not have metastasis or recurrence diagnosed for more than 3 years of follow-up time.

DFS was defined as the period from the date of registration to the time of radiologic confirmation of the recurrence of the primary tumor registered for the TRACERx or the time of death by any cause. Lung cancer–specific DFS was defined as the period from the date of registration to the time of radiologic confirmation of the recurrence of the primary tumor registered for the TRACERx or the time of death from lung cancer.

Kaplan–Meier plots were generated based on the univariate model from the survfit function (survival R package v3.4.0) and compared with the log-rank test. Hazard ratios and *P* values were derived using univariate and multivariable Cox regression analyses with the coxph function (survival v3.4.0). Cox proportional hazards assumptions were fulfilled for univariate and multivariable models. Univariate models also included strata by histology subtype. The multivariable model was adjusted for necrosis, age, sex, pathologic stage (1, 2, 3), smoking pack years, receipt of adjuvant therapy, and histology subtype. The median follow-up time of the cohort was extracted from the summary table of the survfit model.

The necrosis status was evaluated from diagnostic H&E images by a pathologist. High and low recent subclonal expansion scores were defined based on the median score. Hazard ratios of age and pack-years were reported per 10 years. We evaluated several cutoff approaches to define high and low TAN score, median, upper quartile (vs. rest), and optimal cutoff described above in the section *TAN scoring from H&E images in TRACERx*. Both regional and diagnostic TAN scores were evaluated in univariate analysis, whereas the TAN score used for the multivariable analysis was derived from diagnostic H&E images. The association with prognosis remained significant for high TAN tumors in multivariable analyses with median recent subclonal expansion score or *PIK3CA* mutation status (Supplementary Table S5).

### Granulocyte Scoring from H&E Images and Survival Analysis in TCGA

H&E-stained whole-slide images (WSI) from TCGA were analyzed with PathExplore, a deep learning-based model for cell type and tissue classification trained on pathologist-expert annotations on 6,918 WSIs from NSCLC tissue (37). The tissue classification model segments the tumor nests (cancer), cancer stroma, background, necrosis, and artifacts on H&E images. PathExplore outputs for tissue segments were evaluated by pathologists to assess the model's performance in detecting and classifying regions of tissue background, artifact, tumor nests (cancer), cancer stroma, and necrosis. Samples with >10% error for segmenting tumor nests (cancer) and cancer stroma were removed. Samples, for which necrosis, artifacts, and background were not evaluated or had >20% error were excluded. The cell type classification model was trained to predict granulocytes. The granulocyte classification is expected to include eosinophils in addition to neutrophils.

The automated score derived from applying PathExplore on the TCGA cohort used the proportion of granulocytes in the tumor nest/stroma over the total number of cells in the tumor nest/stroma (Supplementary Fig. S11A). The automated score was compatible but not identical to the pathologist-derived TAN score, which was defined based on two factors: the area of neutrophils in the tumor nests/stroma over the area of the tumor nest/stroma. Although the TAN score was based on the cell area proportion, the automated score quantified the cell count proportion. As the area of neutrophils is generally smaller than the area of the cancer cells or fibroblasts in the TS, the pathologist-scored area proportions would be generally lower than the automated cell proportions. Therefore, the TAN score cutoffs derived using the TRACERx cohort could not be directly applied to the TCGA samples. Instead of using a single score cutoff in the TCGA cohort, we used a range of score cutoffs, defined by high granulocyte proportions in the TS. The TAN score and the PathExplore quantification considered only viable tumor islands, excluding necrotic areas and normal tissue.

DFS was defined based on the annotations of new tumor events after initial treatment and vital status in the clinical data downloaded from the TCGA portal (portal.gdc.cancer.gov). The new tumor events annotated as new primary tumors were not considered as DFS events. Patients diagnosed with stage IV tumors or treated for prior malignancy were excluded. The hazard ratio was evaluated for a range of intervals of the two variables: the proportion of granulocytes over all predicted cells in the tumor nest (tumor nest interval) and the stroma (stroma interval). Tumors with high scores were defined as those, for which the proportion of granulocytes in the tumor nest was higher than the tumor nest interval or the proportion of granulocytes in the stroma was higher than the stroma interval.

The multivariable model was adjusted for age, sex, pathologic stage (1, 2, 3), smoking pack years, receipt of adjuvant therapy, and histology subtype. Hazard ratios of age and pack-years were reported per 10 years (Supplementary Fig. S11B).

### Statistical Analysis

All statistical tests were performed in R. No statistical methods were used to predetermine sample size. NSCLC-other was excluded from histology-specific analyses due to a low sample size. Tests involving comparisons of distributions were done using a two-tailed nonparametric Wilcoxon rank-sum test, unless specified one-tailed, using paired or unpaired options where appropriate. Tests involving the comparison of groups were done using a two-tailed Chi-squared test. For all statistical tests, the numbers of data points included are plotted or annotated in the corresponding figure legend. Correlation coefficients and corresponding *P* values were calculated with the Spearman correlation method.

Continuous dependent variable: Where multiple tumor regions/cores per patient were considered, LME models were applied using patient ID as a random effect. The *C6:macrophages and T cells* community was associated with smoking status among the clinical features; therefore, the LME models with this community corrected for smoking status as a fixed effect. ANOVA *P* values were calculated by comparing the effects model to the null model.

Categorical dependent variable: In the case where multiple tumor regions/cores per tumor were considered, statistical analyses of distributions of categorical data (e.g., TME class) with relation to an independent categorical variable (e.g., smoking status) were performed using the lme4 R package glmer function (“binomial” distribution). Patient ID was included as a random effect, and ANOVA *P* values were calculated by comparing the effects model with the null model.

### Code and Data Availability

RNA-seq and WES data (in each case from the TRACERx study) used during this study have been deposited at the European Genome–phenome Archive (EGA), which is hosted by The European Bioinformatics Institute and the Centre for Genomic Regulation (CRG) under the accession codes EGAS00001006517 (RNA-seq) and EGAS00001006494 (WES); access is controlled by the TRACERx data access committee. Details on how to apply for access are available on the linked page. IMC data used or analyzed during this study are available through the CRUK and UCL Cancer Trials Centre (ctc.tracerx@ucl.ac.uk) for academic noncommercial research purposes. Access will be granted upon review of a project proposal, which will be evaluated by a TRACERx data access committee, and entering into an appropriate data access agreement, subject to any applicable ethical approvals.

The code used for IMC analysis in this study was implemented as a Next­flow pipeline and is available on github along with instructions on how to run it on a test data set: https://github.com/FrancisCrickInstitute/TRACERx-PHLEX.

The TRACERx Nuclear IMC segmentation data set, trained neural network model weights, and the test data set can be downloaded from Zenodo: https://zenodo.org/record/7973724.

The core nuclear prediction model, implemented in Python 3, is available here: https://github.com/FrancisCrickInstitute/py-imcyto.

Scripts used for adaptive spillover compensation of IMC isotope channels are available here: https://github.com/FrancisCrickInstitute/TRACERxIMCSpillover.

## Supplementary Material

Supplementary Tables 1-5 Supplementary Table S1. Imaging mass cytometry antibody panel information. Supplementary Table S2. TRACERx 100 imaging mass cytometry cohort information. Supplementary Table S3. TRACERx 421 cohort information. Supplementary Table S4. Median cell densities per histology, tissue type and TME class. Supplementary Table S5. Multivariable model of disease-free survival including TAN score, relevant clinical features together with PIK3CA mutation status (a) or recent subclonal expansion (b).

Supplementary Figures 1-22 Supplementary Figures 1-22 with the corresponding figure legends inline. Supplementary Figure S1. TRACERx 100 imaging mass cytometry cohort. Supplementary Figure S2. Analysis of imaging mass cytometry data. Supplementary Figure S3. Characterisation of cell subtypes and spatial cellular communities in non-small cell lung cancer. Supplementary Figure S4. Clinicogenomic correlations with cell subtypes and communities. Supplementary Figure S5. TME class associations with cell types and clinical variables. Supplementary Figure S6. Cancer cell-intrinsic and -extrinsic features associated with immune cell infiltration. Supplementary Figure S7. Spatial, histological and metabolic features of the Tumour/Stroma:Neutrophil High TME class. Supplementary Figure S8. Transcriptomic features of TS:Neutrophil High TME class and Tumour-Associated Neutrophil scoring. Supplementary Figure S9. Somatic mutations in PIK3CA were associated with neutrophil recruitment through CXCL8 upregulation. Supplementary Figure S10. TAN score association with disease-free survival. Supplementary Figure S11. Validation of the prognostic association from the TAN scoring approach with an automated, deep learning approach in The Cancer Genome Atlas. Supplementary Figure S12. Imaging mass cytometry panel development. Supplementary Figure S13. Spillover matrices for imaging mass cytometry data. Supplementary Figure S14. Multiplexed Consensus Cell Segmentation. Supplementary Figure S15. Investigation of batch effects. Supplementary Figure S16. Raw pixel intensities. Supplementary Figure S17. Pathologist-guided labels. Supplementary Figure S18. Communities methodology and histology associations. Supplementary Figure S19. Multiplexed immunohistochemistry validation of checkpoint molecule expression. Supplementary Figure S20. Cell-cell relationships differ by TME class. Supplementary Figure S21. Cell-cell relationships differ by tumour genomics. Supplementary Figure S22. Cell-cell relationships based on tumour cell phenotypes.

TRACERx Consortium Members All consortium members and their affiliations.
