# Supplementary material for: Spatial Architecture of Myeloid and T Cells Orchestrates Immune Evasion and Clinical Outcome in Lung Cancer
Source: Cancer Discov. 2024 Apr 12;14(6):1018–47. doi: 10.1158/2159-8290.CD-23-1380 (PMC11145179; doi:10.1158/2159-8290.CD-23-1380)
Supplement: Supplementary Figures 1-22 — with the corresponding figure legends inline. Supplementary Figure S1. TRACERx 100 imaging mass cytometry cohort. Supplementary Figure S2. Analysis of imaging mass cytometry data. Supplementary Figure S3. Characterisation of cell subtypes and spatial cellular communities in non-small cell lung cancer. Supplementary Figure S4. Clinicogenomic correlations with cell subtypes and communities. Supplementary Figure S5. TME class associations with cell types and clinical variables. Supplementary Figure S6. Cancer cell-intrinsic and -extrinsic features associated with immune cell infiltration. Supplementary Figure S7. Spatial, histological and metabolic features of the Tumour/Stroma:Neutrophil High TME class. Supplementary Figure S8. Transcriptomic features of TS:Neutrophil High TME class and Tumour-Associated Neutrophil scoring. Supplementary Figure S9. Somatic mutations in PIK3CA were associated with neutrophil recruitment through CXCL8 upregulation. Supplementary Figure S10. TAN score association with disease-free survival. Supplementary Figure S11. Validation of the prognostic association from the TAN scoring approach with an automated, deep learning approach in The Cancer Genome Atlas. Supplementary Figure S12. Imaging mass cytometry panel development. Supplementary Figure S13. Spillover matrices for imaging mass cytometry data. Supplementary Figure S14. Multiplexed Consensus Cell Segmentation. Supplementary Figure S15. Investigation of batch effects. Supplementary Figure S16. Raw pixel intensities. Supplementary Figure S17. Pathologist-guided labels. Supplementary Figure S18. Communities methodology and histology associations. Supplementary Figure S19. Multiplexed immunohistochemistry validation of checkpoint molecule expression. Supplementary Figure S20. Cell-cell relationships differ by TME class. Supplementary Figure S21. Cell-cell relationships differ by tumour genomics. Supplementary Figure S22. Cell-cell relationships based on tumour c [file cd-23-1380_supplementary_figures_1-22_suppsf1.pdf]

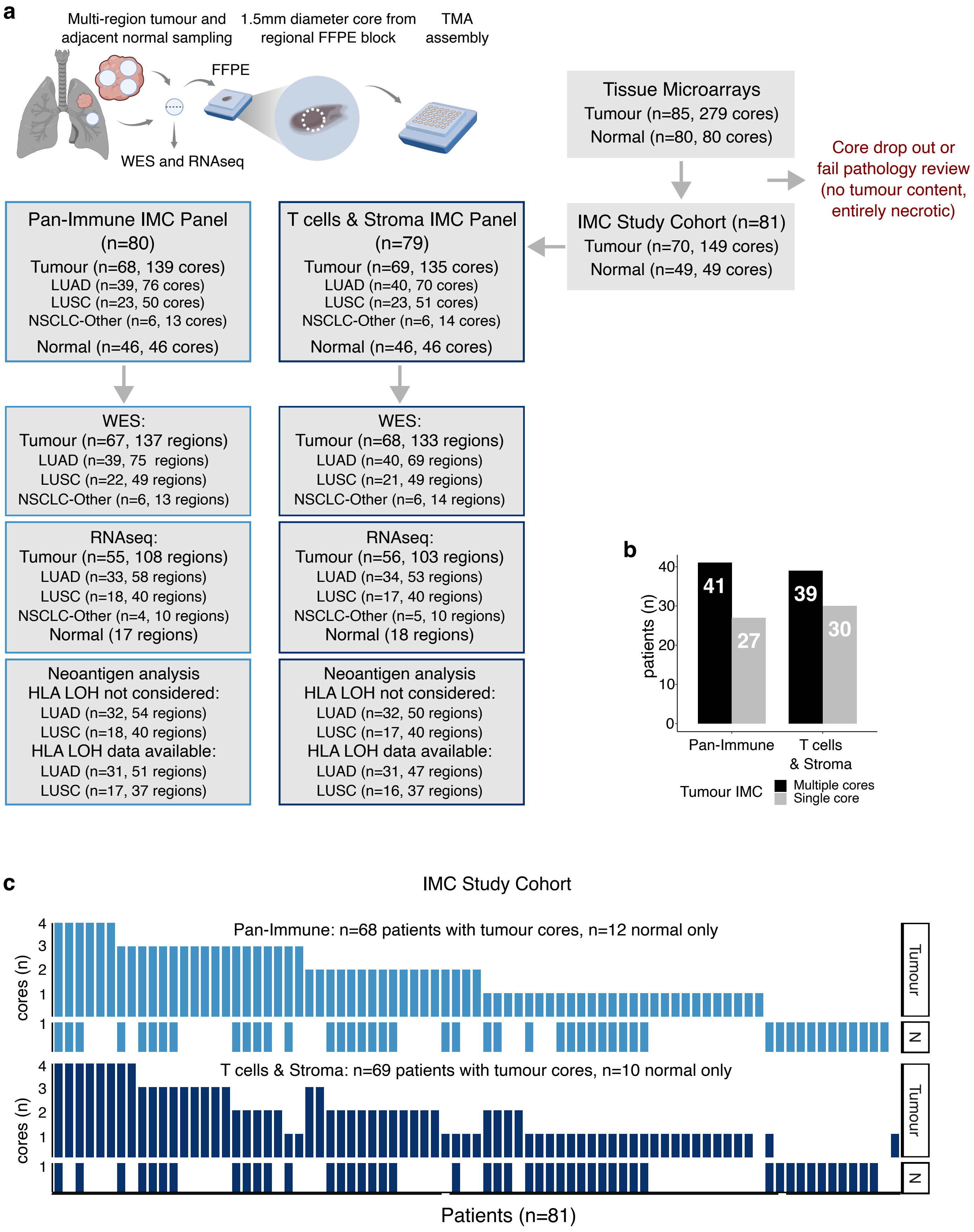

Supplementary Figure S1

Supplementary Figure S1. TRACERx 100 imaging mass cytometry cohort.

a, Illustration of sample processing at time of primary surgery and CONSORT diagram (created with BioRender.com). Excised regions were flash frozen and divided such that one portion was prioritised for DNA and RNA extraction and sequencing, while another portion, given sufficient material, was processed to a Formalin-Fixed Paraffin-Embedded (FFPE) block. Tissue cores were sampled from available regional FFPE blocks and assembled into tissue microarrays (TMA). CONSORT diagram lists the tumour and adjacent normal FFPE cores from the TRACERx 100 cohort embedded into TMAs for which IMC data was available and which passed quality control. Available genomics from the original paired regions is listed, including whole exome sequencing (WES), RNAseq and neoantigen analysis. The neoantigen analysis required neoantigen predictions from WES, expression data from RNAseq (HLA LOH not considered) and HLA LOH data from WES to analyse expressed neoantigens predicted to bind intact HLA alleles (HLA LOH data available). **b**, Number of tumours with single and multiple cores in Pan-Immune and T cells & Stroma IMC panels. **c**, Per patient illustration of the number of tumour and normal (N) cores represented by Pan-Immune and T cells & Stroma IMC panels. LUAD, lung adenocarcinoma; LUSC, lung squamous cell carcinoma; NSCLC, non-small cell lung cancer; HLA LOH, loss of heterozygosity of human leukocyte antigen; IMC, imaging mass cytometry.

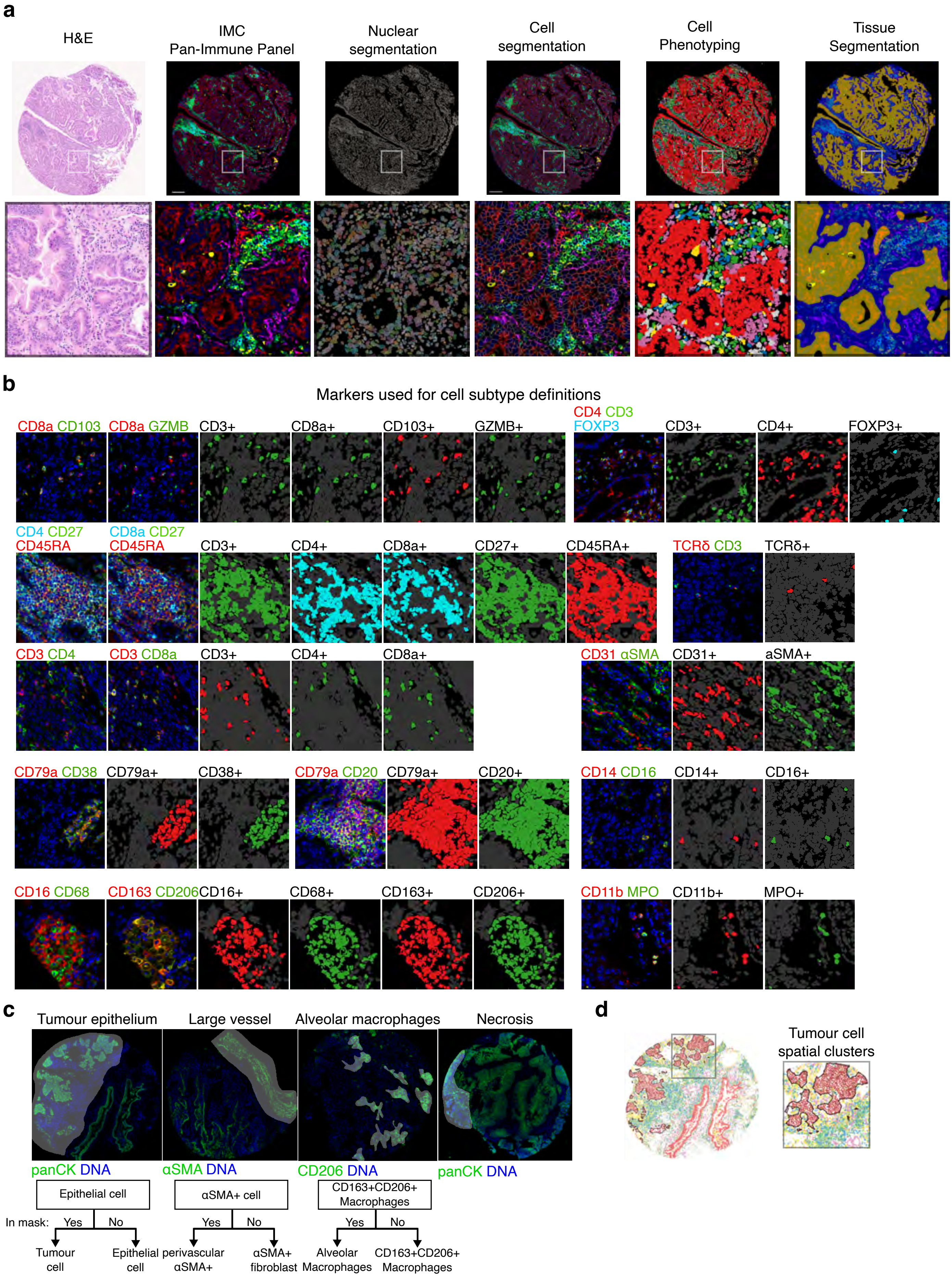

Supplementary Figure S2

Supplementary Figure S2. Analysis of imaging mass cytometry data.

a, Overview of the analytical steps for a representative tumour core. In order from left to right: H&E stain, pseudocoloured IMC channels, machine learning-guided nuclear segmentation, whole cell segmentation, single cell phenotyping and tissue segmentation. H&E and imaging mass cytometry (IMC) images were derived from two serial tissue sections. Cropped area is 160,000µm^2. **b,** An intermediate step of the phenotyping procedure includes automated positivity calling of each marker in the study. Positivity calls for markers defining cell subtypes are shown alongside pseudocoloured composite IMC images with DNA channel shown in blue. Crops are 40,000µm^2. **c,** Pathologist-guided labels overlaid onto representative composite IMC images of indicated markers. These labels were used to further stratify the indicated cell subtypes downstream of cell phenotyping. Additionally, necrosis labelling was used to define presence of absence in a core. **d,** Spatial clustering of tumour cells. H&E, haematoxylin and eosin.

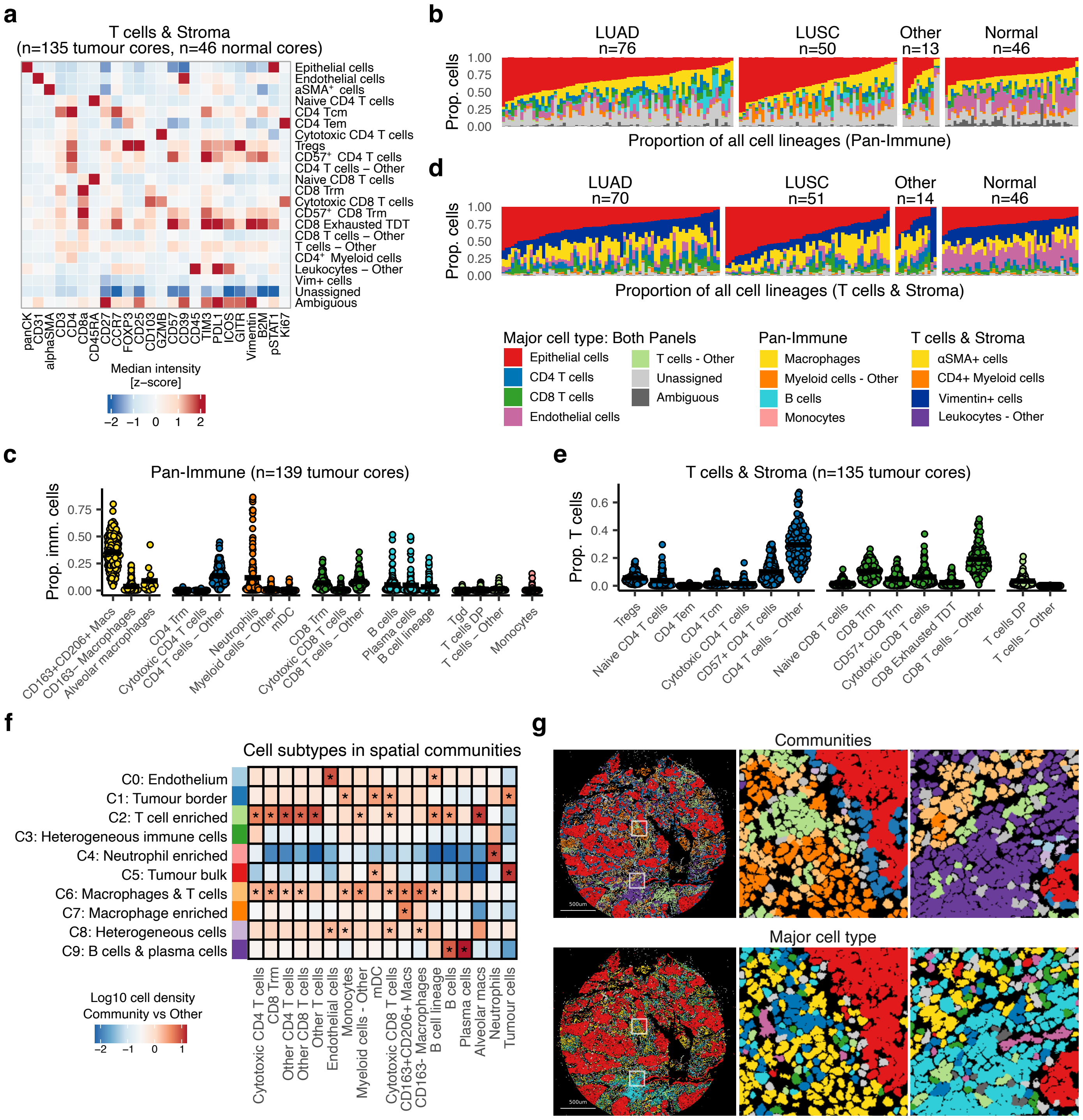

Supplementary Figure S3

Supplementary Figure S3. Characterisation of cell subtypes and spatial cellular communities in non-small cell lung cancer.

a, A heatmap of the z-score normalised median intensities of markers from the T cells & Stroma panel across the identified cell subtypes. **b,** Proportion of major cell types over all cell lineages identified in the Pan-Immune IMC dataset quantified per TMA core. **c,** Proportion of different immune cell subtypes over all immune cells identified in the Pan-Immune IMC dataset quantified per tumour core. **d,** Proportion of major cell types over all cell lineages identified in the T cells & Stroma IMC dataset quantified per TMA core. **e,** Proportion of different T cell subtypes over all T cells identified in the T cells & Stroma IMC dataset quantified per tumour core. **f,** Cell subtypes enriched in each spatial cellular community (n=139 tumour cores, 68 tumours). The heatmap shows the log10-transformed fold change between the mean cell density of a community and all other communities combined. P-values derived from linear mixed effects models with patient as a random covariate to adjust for multiple cores per tumour, comparing the effect model to a null model. P-values are adjusted for multiple testing using the Benjamini-Hochberg method. *:p.adj<0.05. **g,** Example of cells annotated by community (top row) and cell type (bottom row). Scale bar=500µm. Cell objects were represented according to the community colours in **f** or the major cell type colour legend below panel **d** (Pan-Immune). LUAD, lung adenocarcinoma; LUSC, lung squamous cell carcinoma; Other, other non-small cell lung cancer histologies; Tem, effector memory T cell; Tcm, central memory T cell; Trm, resident memory T cell; TDT, Terminally Differentiated T cell; DP, double positive ($CD4^+CD8a^+$).

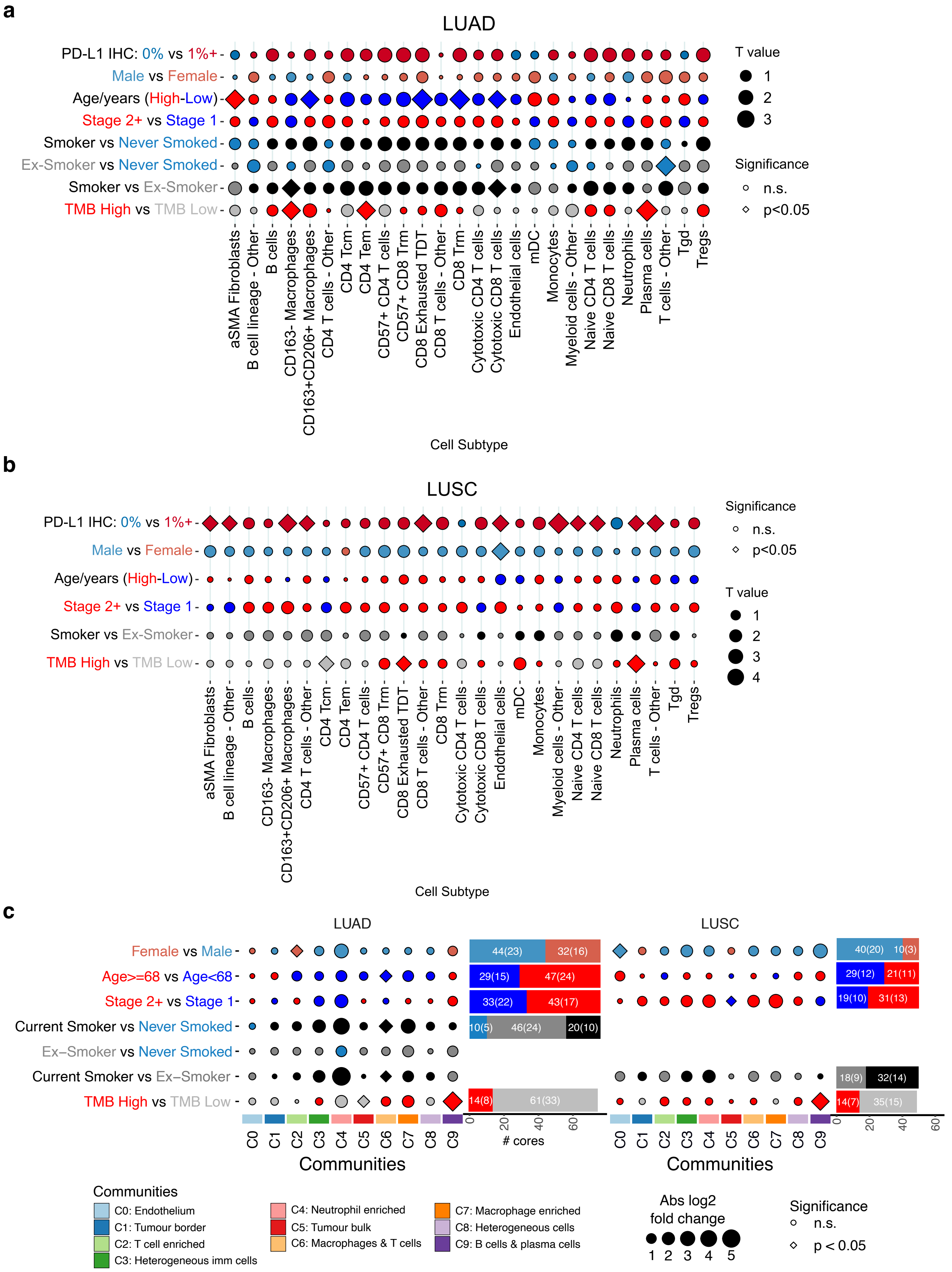

Supplementary Figure S4

Supplementary Figure S4. Clinicogenomic correlations with cell subtypes and communities.

a-b, Associations between cell subtype densities and clinicogenomic features in lung adenocarcinoma (LUAD) (non-TMB features: T cells & Stroma: n=70 cores, 40 tumours, Pan-Immune: n=76 cores, 39 tumours; TMB: T cells & Stroma: n=69 cores, 40 tumours, Pan-Immune: n=75 cores, 39 tumours) **(a)** and lung squamous cell carcinoma (LUSC) tumour cores (non-TMB features: T cells & Stroma: n=51 cores, 23 tumours, Pan-Immune: n=50 cores, 23 tumours; TMB: T cells & Stroma: n=49 cores, 21 tumours, Pan-Immune: n=49 cores, 22 tumours) **(b)**. The colour of the shape indicates the directionality of the clinical variable (row) in which the cell subtype (column) is enriched, as indicated by the text colours of the clinical variables. Significant associations are shown with diamonds ($p<0.05$) and non-significant (n.s.) associations with circles. The size of the shape indicates the T value. P-values and T values were derived from a LMEM with cell subtypes density as a dependent variable, clinical variable as a fixed effect and patient as a random covariate. **c,** Associations between spatial community densities with clinicogenomic features. The p-value, shown unadjusted, is calculated from the ANOVA test between the LMEM model versus a null model with patient as a random covariate. The corresponding bar plots show the number of tumour cores and the number of patients in brackets. PD-L1 immunohistochecmistry (IHC) score in **a, b** refers to tumour cell scoring. TMB High: ≥10 mutations/Mb, TMB Low: <10 mutations/Mb. LUAD, lung adenocarcinoma; LUSC, lung squamous cell carcinoma; NSCLC, non-small cell lung cancer; LMEM, linear mixed effects model; TMB, tumour mutation burden; Tcm, central memory T cell; Tem, effector memory T cell; TDT, terminally differentiated T cell.

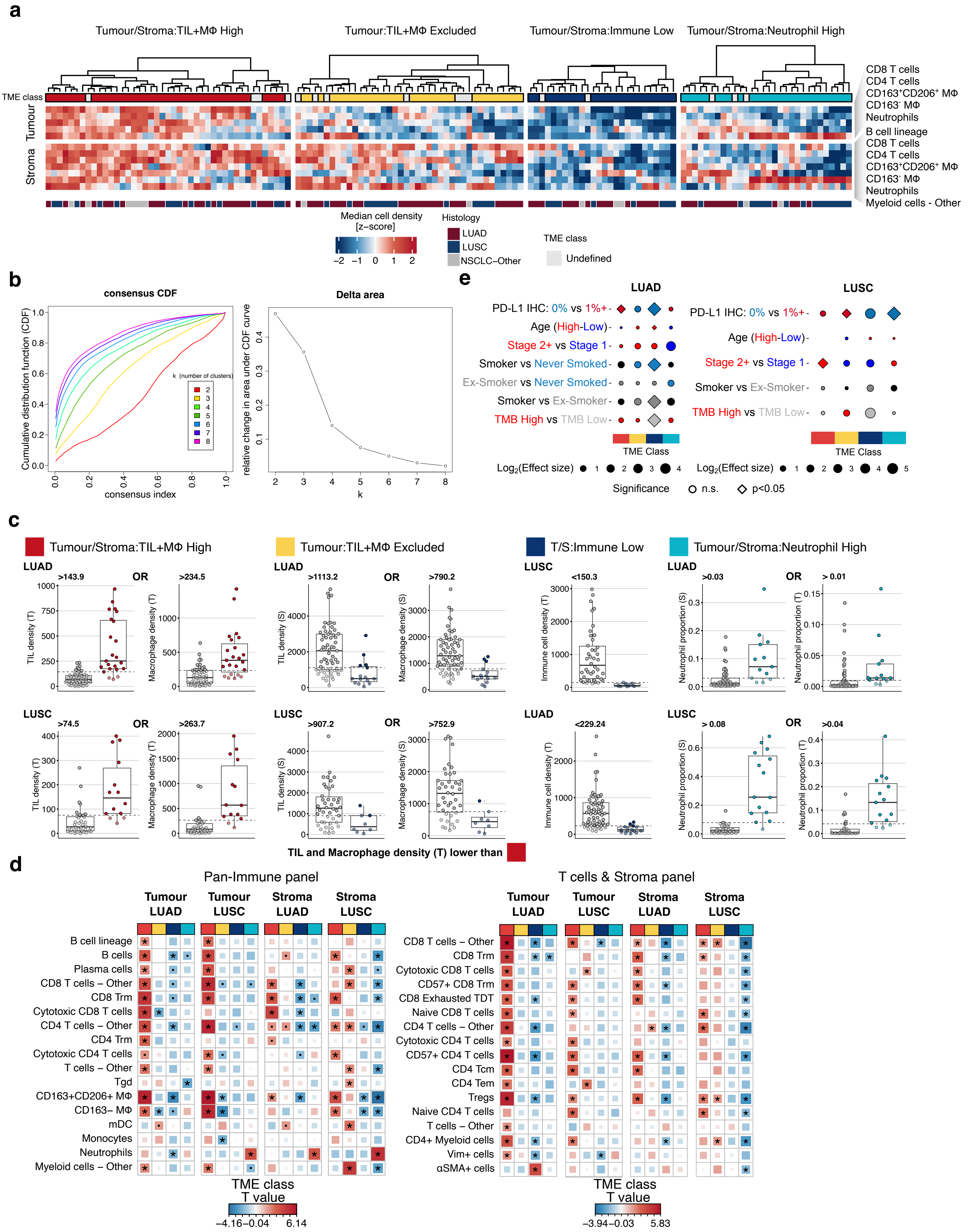

Supplementary Figure S5

Supplementary Figure S5. TME class associations with cell types and clinical variables.

a, Four immune clusters derived from unsupervised hierarchical clustering of immune cell densities in the tumour nest and stroma. In addition to the samples shown in **Figure 2a** (n=65 LUAD, 48 LUSC tumour cores), NSCLC-Other cores (n=10) and a subset of tumour cores labelled with *Undefined* TME class (n=11 LUAD, 2 LUSC, 3 NSCLC-Other) were visualised within each of the immune clusters. Cell density represents cells/mm^2. **b,** The cumulative distribution function (CDF) of the consensus matrix for each value of k, where k is the number of clusters (left). The difference in area under the curve comparing the CDF for k with CDF for k-1 (right). Consensus clustering for TME class definition was performed by clustering a randomly sampled subset of the data. Pairwise consensus values represented the proportion of subsampling iterations where two samples were assigned to the same clusters. **c,** Criteria required to be fulfilled by all tumour cores in each TME class. The cores that did not fulfil these criteria were labelled *Undefined*. *TS:TIL+Mϕ High* cores were required to have higher TIL or macrophage densities (cells/mm^2) in the tumour nest (T); *TS:Immune Low* cores - lower immune cell density in the tumour nest; *T:TIL+Mϕ Excluded* TME - lower TIL or macrophage density in the tumour nest than *TS:TIL+Mϕ High* and higher TIL or macrophage density in the stroma (S) than *TS:Immune Low; TS:Neutrophil High* cluster - higher neutrophil proportion over all cells than other TME classes in the tumour nest or stroma. The cutoffs for each of these criteria were determined automatically using a binomial generalised linear model for each TME class compared to other classes. **d,** Significant enrichments and depletions of intratumour and stroma-localising cell subtypes within tumour cores from each TME class when compared to tumour cores from other TME classes combined for each of the Pan-Immune panel (LUAD n=65 cores, LUSC n=48 cores) and T cells & Stroma panel (LUAD n=57 cores, LUSC n=45 cores). P-values and T values were derived from an LMEM using patient as a random effect. Significant associations are indicated with an asterisk ($p<0.05$). The T value for enrichment-depletion is shown on the red-blue scale, respectively. **e**, TME class associations with clinical variables for LUAD (PD-L1: n=45 cores, 31 tumours; TMB: n=64, 36; other: n=65, 36) and LUSC (PD-L1: n=37, 19; TMB: n=47, 22; other: n=48, 23). TMB High: ≥10muts/Mb, TMB Low: <10muts/Mb. The colour of the shape indicates the directionality of the clinical variable (row) in which the TME class (column) is enriched, as indicated by text colours of the clinical variables. The size of the shape represents the $\log_2$ of the effect size, where effect size is equivalent - for categorical variables - to the ratio of the proportion of cores of the TME class of interest between the two categories of the clinical variable, and - for age, a continuous variable (units: years) - to the ratio of the median age in the TME class of interest to the median age in the other TME classes combined, with age assigned for each core. The p-value is calculated from an analysis of variance of a generalised LMEM with TME class as

dependent variable, clinical variable as a fixed effect and patient as a random effect, compared to a null model. LMEM, linear mixed effects model; LUAD, lung adenocarcinoma; LUSC, lung squamous cell carcinoma; Tumour/Stroma, TS; Tumour, T; TIL, tumour-infiltrating lymphocyte; MΦ, macrophage.

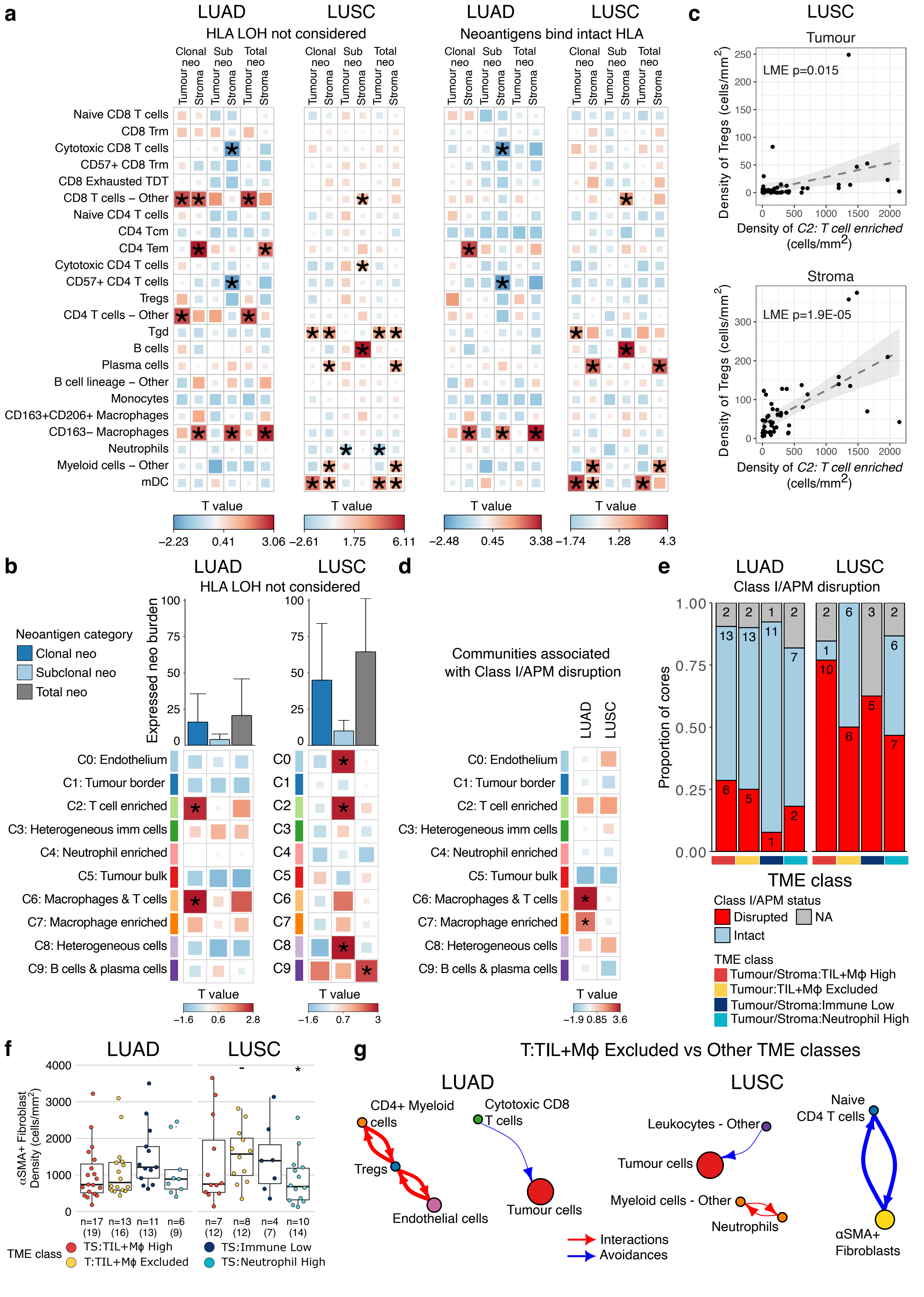

Supplementary Figure S6

Supplementary Figure S6. Cancer cell-intrinsic and -extrinsic features associated with immune cell infiltration.

a, Correlation of intratumour and stroma-localising immune cell densities in LUAD and LUSC tumour cores with clonal, subclonal and total neoantigen burden before (left) and after (right) considering HLA LOH. The T value for enrichment-depletion is shown on the red-blue scale, respectively. **b,** Correlation of densities of spatial cellular communities and clonal, subclonal and total neoantigen (neo) burden before accounting for HLA LOH in LUAD and LUSC tumour cores. Bar plot shows the median with whiskers extending to the 75th percentile. 54 LUAD cores (n=32 tumours) and 40 LUSC cores (n=18 tumours). **c**, Linear regression between the density of *C2:T cell enriched* communities and intratumour and stroma-localised Treg densities in LUSC (n=22 patients, 47 tumour cores). **d,** Association between the density of spatial cellular communities with Class I/APM disruption. LUAD: 16 cores (11 tumours) with Class I/APM disruption, 51 cores (28 tumours) with intact Class I/APM. LUSC: 29 cores (15 tumours) with Class I/APM disruption, 14 cores (8 tumours) with intact Class I/APM. Boxes in **a-b, d** correspond to T values. **e**, The proportion of tumour cores in each TME class harbouring Class I/APM disruption, stratified by histology. Numbers indicate the number of tumour cores. NA values represent tumour regions for which HLA status could not be resolved and no mutations were detected in APM genes. **f**, Boxplot comparing αSMA^{+} fibroblast densities in a given TME class compared to all other TME classes combined in LUAD (n=36 patients, 57 cores) and LUSC (n=22 patients, 45 cores) tumour cores. Boxplots show median and lower and upper quartile values, and whiskers extend up to 1.5*IQR above and below the quartiles. **g**, Network diagram illustrating the cell-cell relationships that were significantly enriched in *T:TIL+MΦ Excluded* (Pan-Immune: 20 LUAD cores, 12 LUSC cores; T cells & Stroma: 16 LUAD cores, 12 LUSC cores) compared to other TME classes combined (Pan-Immune: 56 LUAD cores, 36 LUSC cores; T cells & Stroma: 50 LUAD cores, 33 LUSC cores). The frequency of the cell-cell relationships in tumour cores derived from neighbouRhood permutation testing was compared between tumour cores with *T:TIL+MΦ Excluded* TMEs and other TME classes in a logistic regression model. Only significant relationships that were present in at least 30% of tumour cores and of which constituent cells were present in at least 90% of tumour cores were illustrated. The size of the network node corresponds to the median cell density across all tumour cores, per histology. **a-d, f,** P-values and T values were derived from a linear mixed effects (LME) model with patient as a fixed covariate to account for multiple cores per tumour, using smoking status as a fixed effect in **a** with a p-value<0.05 considered significant. Significance in **d** held after adjusting for smoking history. -:p<0.1, *:p<0.05. LUAD, lung adenocarcinoma; LUSC, lung squamous cell carcinoma; panCK, pancytokeratin; Tumour/Stroma, TS; Tumour, T; TIL, tumour-infiltrating lymphocytes; MΦ, macrophage.

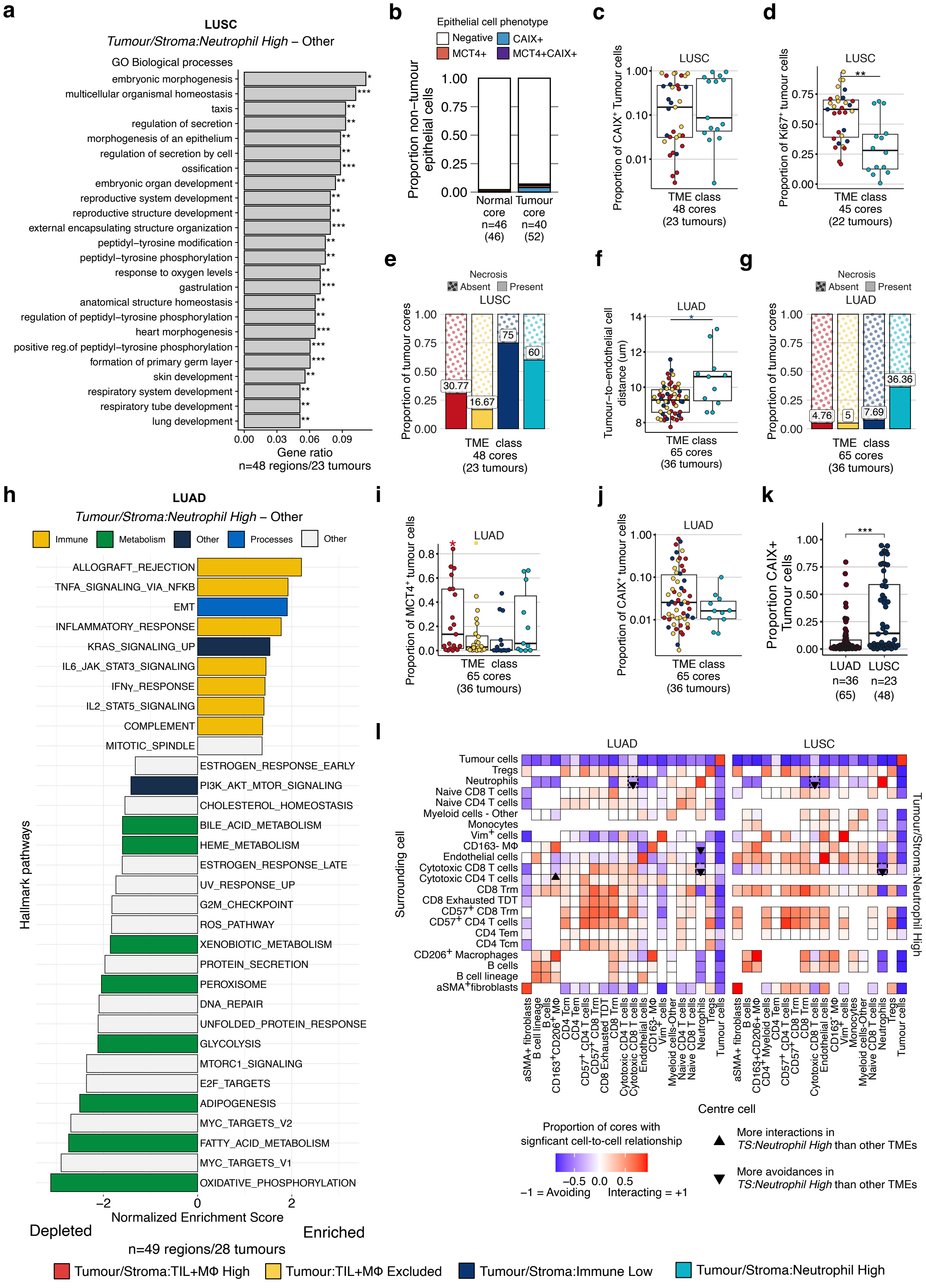

Supplementary Figure S7

Supplementary Figure S7. Spatial, histological and metabolic features of the *Tumour/Stroma:Neutrophil High* TME class.

a, Remaining Gene Ontology (GO) biological processes enriched among up-regulated genes in *TS:Neutrophil High* TME class (n=10 cores) compared to other TME classes combined (n=28 cores) in LUSC (FDR<0.01, gene ratio>0.05), in addition to **Figure 4a**. **b**, Proportion of non-tumour epithelial cells (co-)expressing MCT4 and/or CAIX in normal and tumour cores from patients with NSCLC. **c-d,** Proportion of tumour cells assigned as $CAIX^+$ **(c)** or Ki67+ **(d)** per LUSC tumour core in *TS:Neutrophil High* TMEs compared to other TME classes. **e**, Frequency of LUSC tumour cores with presence of necrotic areas, as detected by histopathological review of paired regional H&Es, across TME classes. **f,** Median distance between tumour cells to their nearest endothelial cell per LUAD tumour core in *TS:Neutrophil High* TME class compared to all other TME classes combined. **g**, Frequency of LUAD tumour cores with presence of necrotic areas, as detected by histopathological review of paired regional H&Es, across TME classes. **h,** Gene-set enrichment analysis (GSEA) of hallmark gene sets compared between LUAD tumour cores from the *TS:Neutrophil High* TME class and other TME classes combined, using the t-statistic derived from the limma-voom model on TMM-normalised gene expression. Significantly enriched pathways were coloured by type of pathway (FDR<0.05). **i,** Proportion of tumour cells assigned as $MCT4^+$ per tumour core in LUAD by TME class. **j,** Proportion of tumour cells assigned as $CAIX^+$ per LUAD tumour core in *TS:Neutrophil High* TMEs compared to other TME classes. **k,** Proportion of tumour cells assigned as $CAIX^+$ in LUAD compared to LUSC tumour cores. Tumour cores with an *Undefined* TME class were excluded. **l,** A heatmap coloured by the proportion of *TS:Neutrophil High* tumour cores with a significant cell-cell relationship in LUAD (left) and LUSC (right). Cell-to-cell relationships were determined using neighbourhood permutation analysis per tumour core as interactions (+1,▲) or avoidances (-1,▼). Triangles indicate whether the frequency of a cell-cell relationship was significantly different in the *TS:Neutrophil High* class compared to the other TME classes combined, using a logistic regression model accounting for multiple cores per tumour, unadjusted p-value<0.05. Squares with dashed borders are highlighted in the text. Significantly enriched cell-cell relationships were reported if present in at least 30% of tumour cores. Cell subtypes present in less than 90% of tumour cores were not compared (no square). LUAD: *TS:Neutrophil High* vs Other (n=11 cores, 6 tumours vs n=69 cores, 37 tumours); LUSC: *TS:Neutrophil High* vs Other (n=15 cores, 11 tumours vs n=39 cores, 18 tumours). P-values for **c-d, f, i-k** were derived from a LMEM with patient as a random covariate to adjust for multiple cores per tumour, and represent the comparison of a given TME class compared to other TME classes combined. The TME class colour legend at the bottom of the figure applies to panels **c-j**. Boxplots show median and lower and upper quartile values, and

whiskers extend up to 1.5*IQR above and below the quartiles. Boxplot x-axes labelled in the format ‘n=X (Y)’ show X as the number of patients for that histology and TME class combination and Y the number of tumour cores. •:$p<0.1$, *:$p<0.05$, **:$p<0.01$, ***:$p<0.001$; LUAD, lung adenocarcinoma; LUSC, lung squamous cell carcinoma; TS, Tumour/Stroma, TMM, trimmed mean of *M*-values; LMEM, linear mixed effects model; TIL, tumour-infiltrating lymphocyte; MΦ, macrophage.

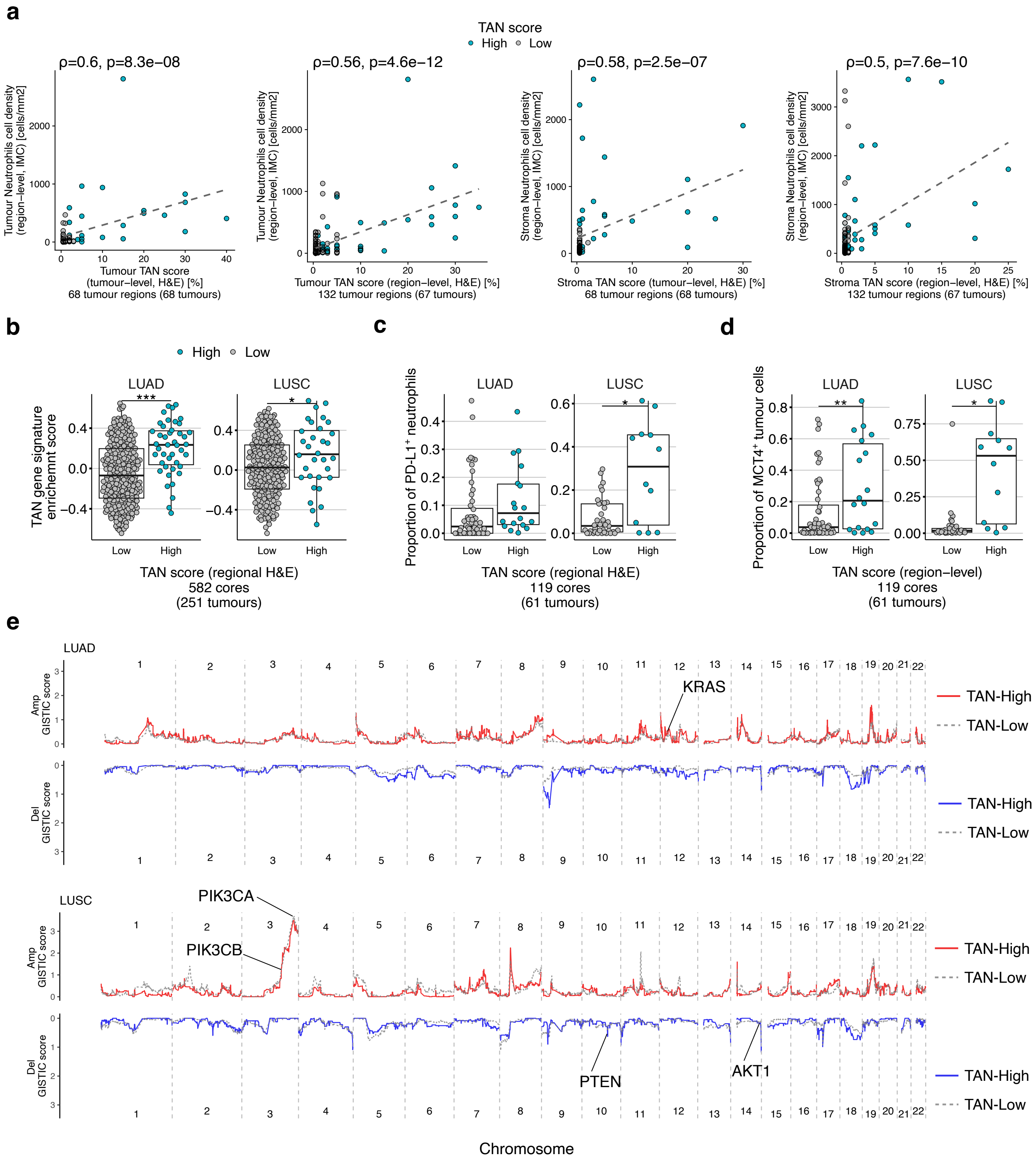

Supplementary Figure S8

Supplementary Figure S8. Transcriptomic features of *TS:Neutrophil High* TME class and Tumour-Associated Neutrophil scoring.

a, Scatter plots of H&E-derived tumour- and region-level tumour-associated neutrophil (TAN) scores in tumour nest and stroma on x-axes and the IMC-derived neutrophil cell densities (y-axis). IMC-derived densities in tumour-level correlations represent the median value in the case of multi-region tumour data. Each tumour region is represented as a point coloured by the corresponding TAN group, TAN-High and TAN-Low. Spearman correlation coefficients and p-values are shown. **b,** Using transcriptional signatures for neutrophil subsets defined in Salcher *et al.*, the TAN-High tumour cores had a significantly higher enrichment score for TAN gene signatures in LUAD and LUSC. **c-d,** Proportions of PD-L1^{+} neutrophils **(c)** and MCT4^{+} tumour cells **(d)** between region-level TAN-High and TAN-Low tumour cores. **e,** Comparison of the GISTIC2.0 across-genome copy number profiles between TAN-High and TAN-low tumour regions (region-level TAN groups) in LUAD and LUSC, to assess significantly occurring somatic alterations of components in the upregulated PI3K and RAS/MAPK/ERK signalling pathways. Chromosome numbers are annotated. P-values in **b-d** were derived from LMEM on region-level TAN groups with patient as a random effect. *:p<0.05, ***:p<0.001; LMEM, linear mixed effects model; H&E, haematoxylin and eosin; LUAD, lung adenocarcinoma; LUSC, lung squamous cell carcinoma; Amp, amplification; Del, deletion.

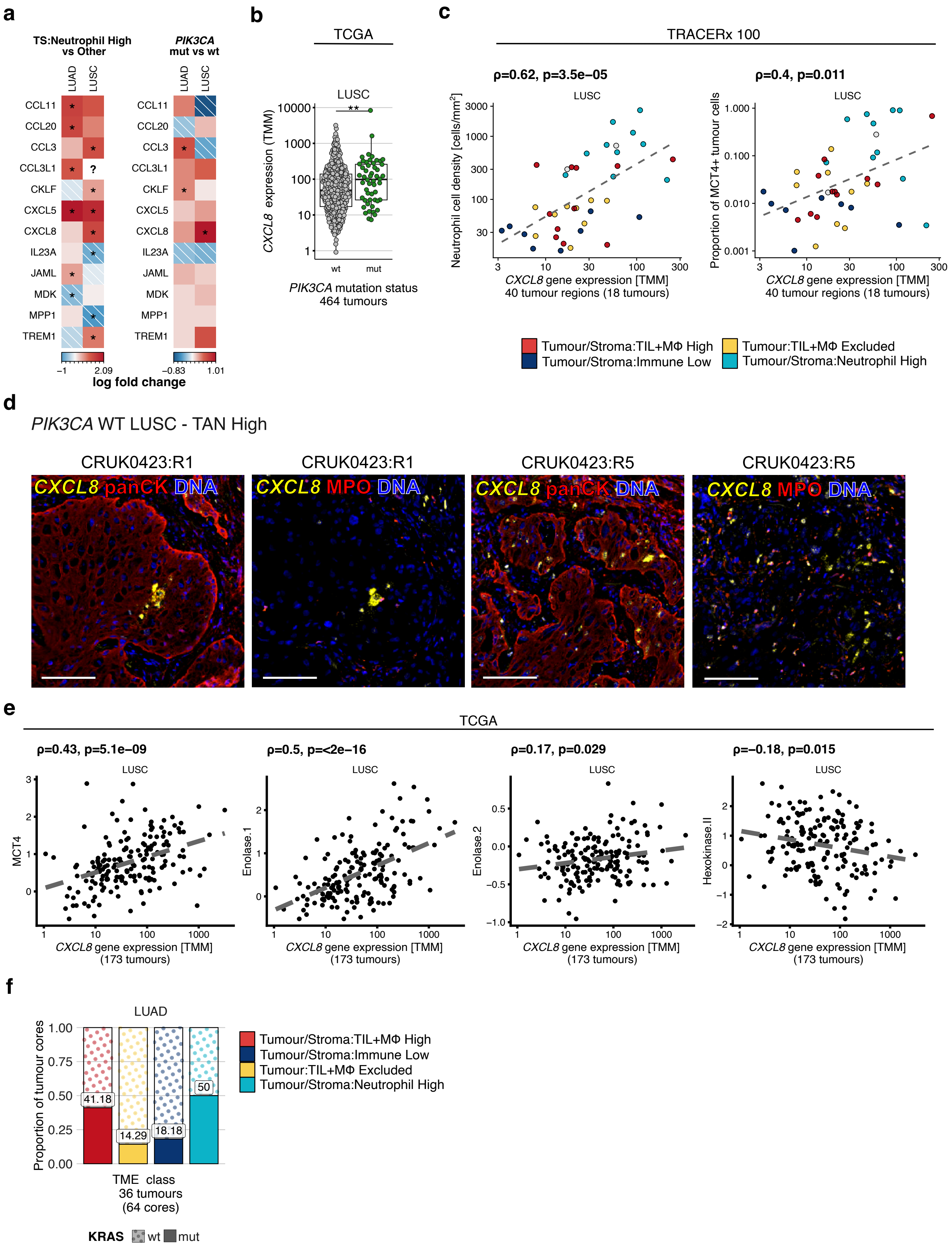

Supplementary Figure S9

Supplementary Figure S9. Somatic mutations in *PIK3CA* were associated with neutrophil recruitment through *CXCL8* upregulation.

a, The subset of differentially expressed genes from the gene set *GOBP_NEUTROPHIL_CHEMOTAXIS* in Gene Ontology. The heatmap on the left shows the differentially expressed genes between *Tumour/Stroma:Neutrophil High* TME class compared to other TME classes combined (left) and the right between the *PIK3CA* mutant (mut) compared to *PIK3CA* wild type (wt) tumour regions (right). P-values were derived from a limma-voom differential expression analysis of TMM expression correcting for multiple regions per tumour. '?', expression not detected. **b,** Comparison of TMM expression values for *CXCL8* between *PIK3CA*mut and *PIK3CA*wt tumour regions in the TCGA LUSC cohort. **c,** Spearman correlation of *CXCL8* expression with neutrophil cell densities (left) and the proportion of $MCT4^{+}$ tumour cells in LUSC tumour cores with paired data in the TRACERx 100 cohort. Data points are coloured by TME class and represent individual tumour cores. **d,** Immunofluorescence images of *CXCL8* RNAscope multiplexed with antibody staining of pancytokeratin (panCK) or MPO in a LUSC patient with multiple TAN-High tumour regions and wild type (WT) *PIK3CA*. Scale bar represents 100µm. **e,** Spearman correlations of *CXCL8 gene* expression (x-axis) with expression of proteins involved in glycolysis (y-axis), using reverse phase protein assays from the TCGA LUSC cohort. Only proteins that passed the antibody quality control were considered. **f,** Proportion of tumour cores with *KRAS* mutations by TME class in LUAD. ·:p<0.1, *:p<0.05, **:p<0.01, ***:p<0.001; TMM, Trimmed mean of *M*-values; LUAD, lung adenocarcinoma; LUSC, lung squamous cell carcinoma.

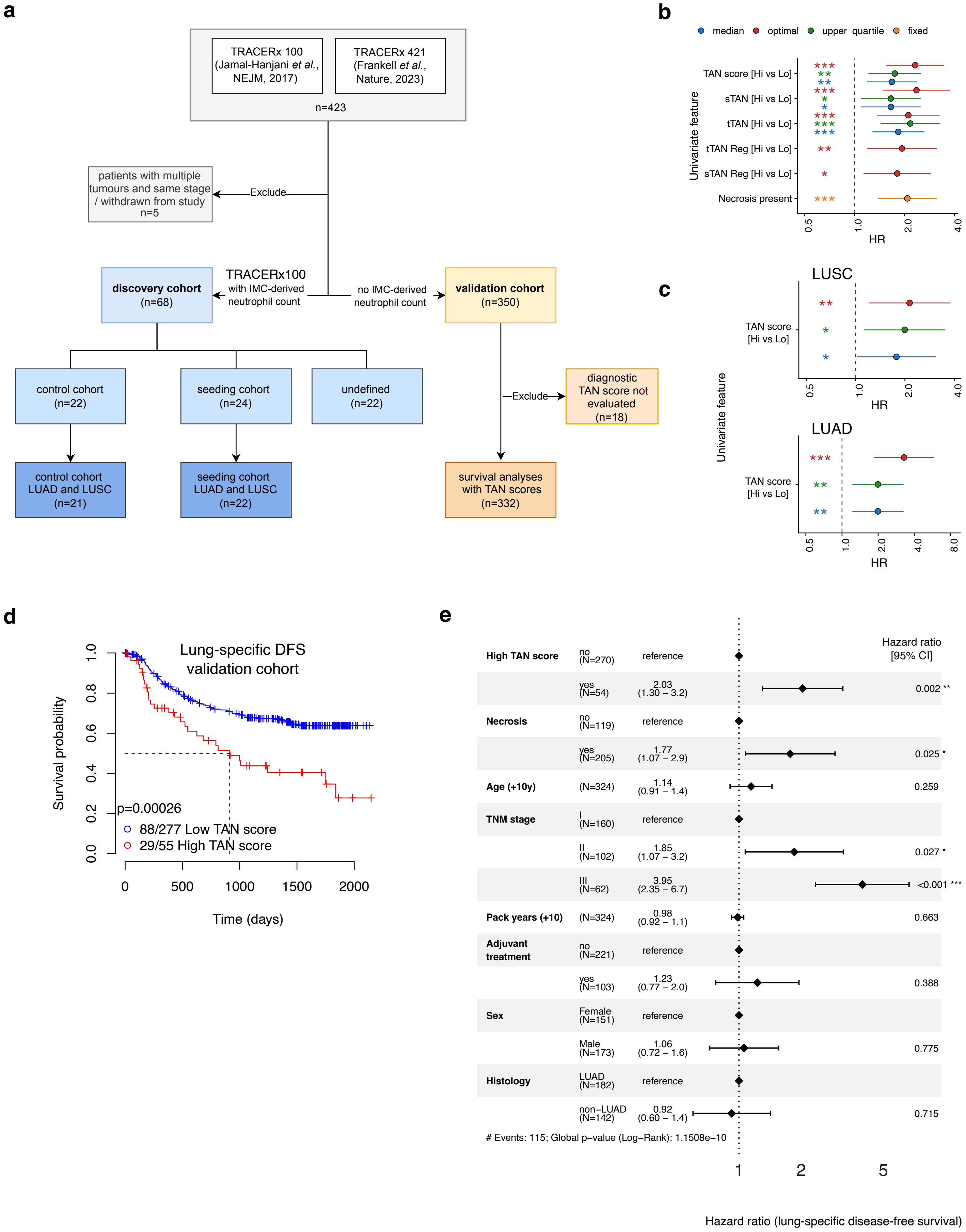

Supplementary Figure S10

Supplementary Figure S10. TAN score association with disease-free survival.

a, CONSORT diagram of discovery and validation TRACERx cohorts. Five patients with incomplete clinical, pathological or genomic information were excluded. In the discovery cohort, tumours classified as *Undefined* did not seed a metastasis or recurrence but had a follow-up time shorter than 3 years, or genomic information was not available to determine whether the primary tumour seeded a metastasis (Methods). **b,** Univariate associations with DFS for tumour-level TAN score (TAN score), tumour-level TAN score in tumour nests (tTAN), tumour-level TAN score in stroma regions (sTAN), region-level TAN score in tumour nest (tTAN Reg, maximum per tumour), region-level TAN score in stroma (sTAN Reg, maximum per tumour) and necrosis presence. **c**, Univariate analysis of DFS by tumour-level TAN scores for LUAD and LUSC separately. **d,** Kaplan-Meier curve for lung cancer-specific DFS by tumour-level TAN score in NSCLC. P-value derived from univariate Cox model adjusted for histology. **e,** Multivariable Cox proportional hazards model using tumour-level TAN score for lung cancer-specific DFS. LUAD, lung adenocarcinoma; LUSC, lung squamous cell carcinoma; TAN, tumour-associated neutrophil; DFS, disease-free survival; CI, confidence interval; HR, hazard ratio; *:p<0.05, **:p<0.01, ***:p<0.001.

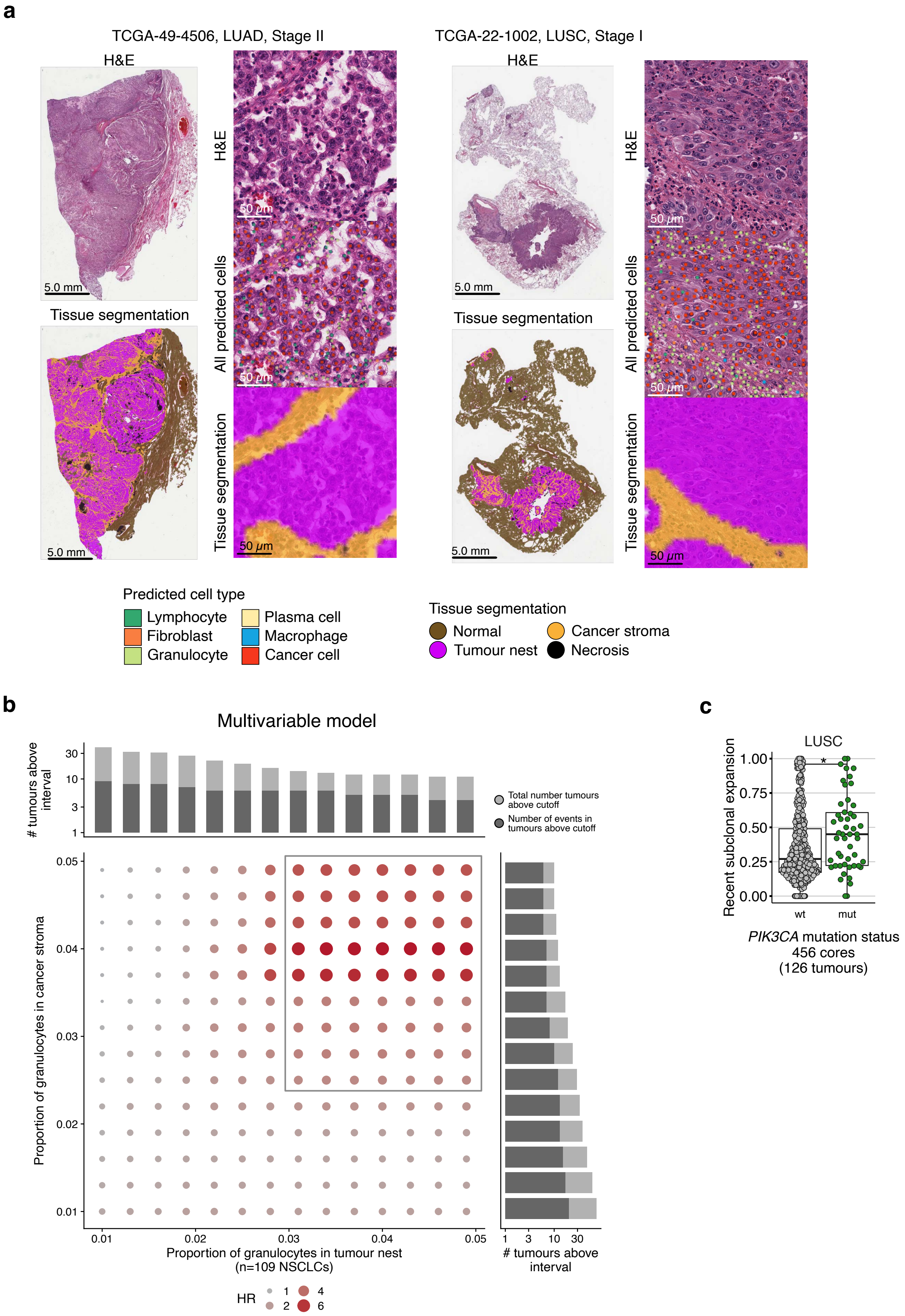

Supplementary Figure S11

Supplementary Figure S11. Validation of the prognostic association from the TAN scoring approach with an automated, deep learning approach in The Cancer Genome Atlas.

a, Representative images of high granulocyte infiltration in the tumour nest and stroma for LUAD (left) and LUSC (right). **b,** Multivariable analyses were performed for a range of cutoffs of the granulocyte proportion in the tumour nest (x-axis) and the stroma (y-axis). The multivariable model included the automated scoring (high vs low), age, sex, adjuvant treatment, pack years, histology subtype and TNM stage (n=109 NSCLC). Tumours were assigned a high score when the granulocyte proportion in the tumour nest or stroma was higher than the respective tumour nest- or stroma cutoffs on the x- and y-axes. The point size and colour represent the corresponding hazard ratio. All cutoffs highlighted in the rectangle have statistical significance for the automated score in a Cox multivariable model ($p<0.05$). An interval size of 0.03% was used for visualisation. **c,** Comparison of recent subclonal expansion scores in *PIK3CA* mutant (mut) and wild type (wt) tumour regions. Boxplots show median and lower and upper quartile values, and whiskers extend up to 1.5*IQR above and below the quartiles. P-value was derived from linear mixed effects model on region-level data with patient as a random effect. LUAD, lung adenocarcinoma; LUSC, lung squamous cell carcinoma; NSCLC, non-small cell lung cancer; HR, hazard ratio; *:$p<0.05$.

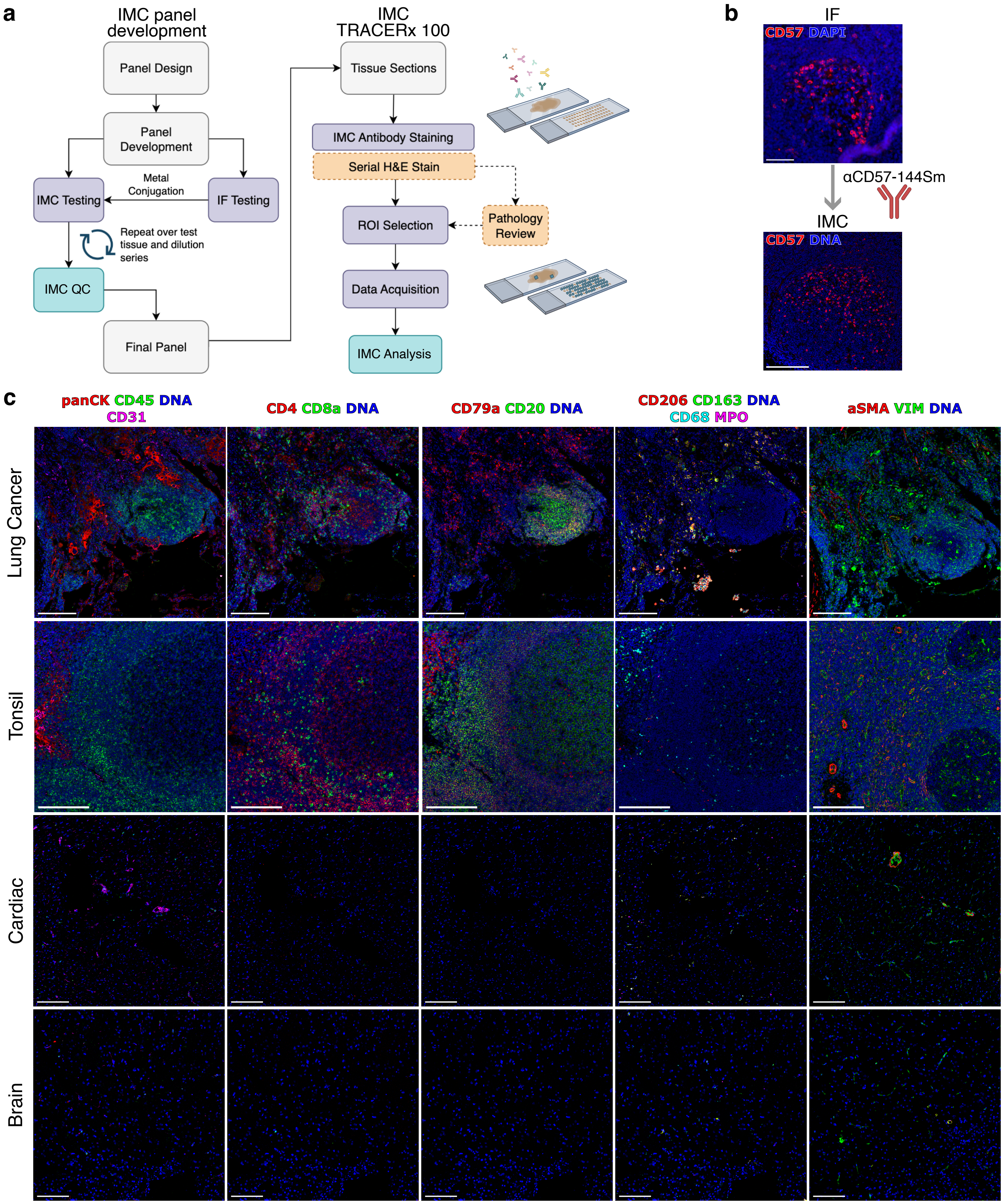

Supplementary Figure S12

Supplementary Figure S12. Imaging mass cytometry panel development.

a, Depiction of imaging mass cytometry (IMC) antibody panel development and application in the TRACERx 100 cohort, which included **b,** antibody testing by immunofluorescence (IF) on control tissues prior to metal conjugation and re-evaluation by IMC (Created with BioRender.com). **c,** All antibodies were tested by IMC on representative lung-derived tissues and staining controls as guided by a pathologist, with a selection of markers representing cell lineages assessed in the study shown. Scale bar=200µm.

a

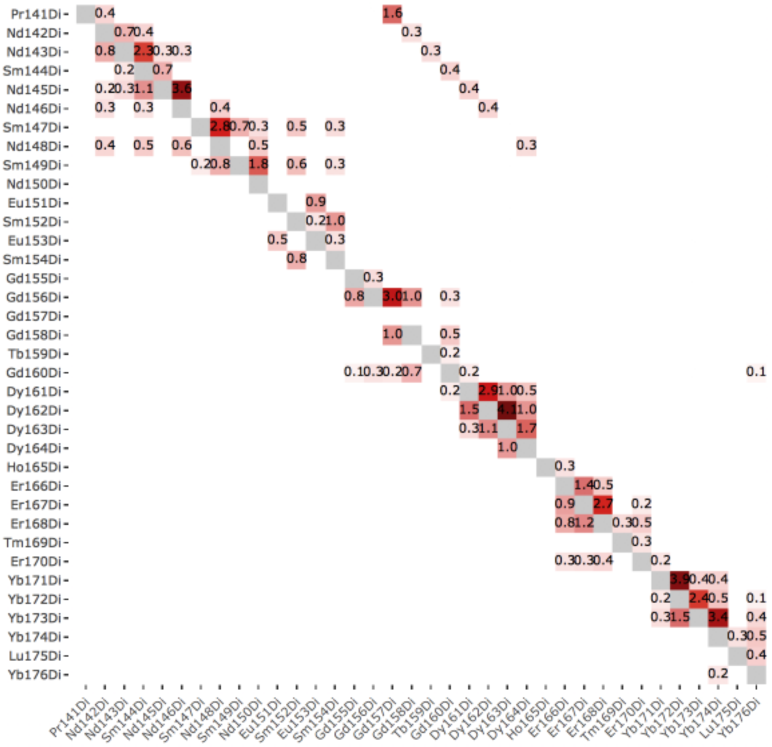

b

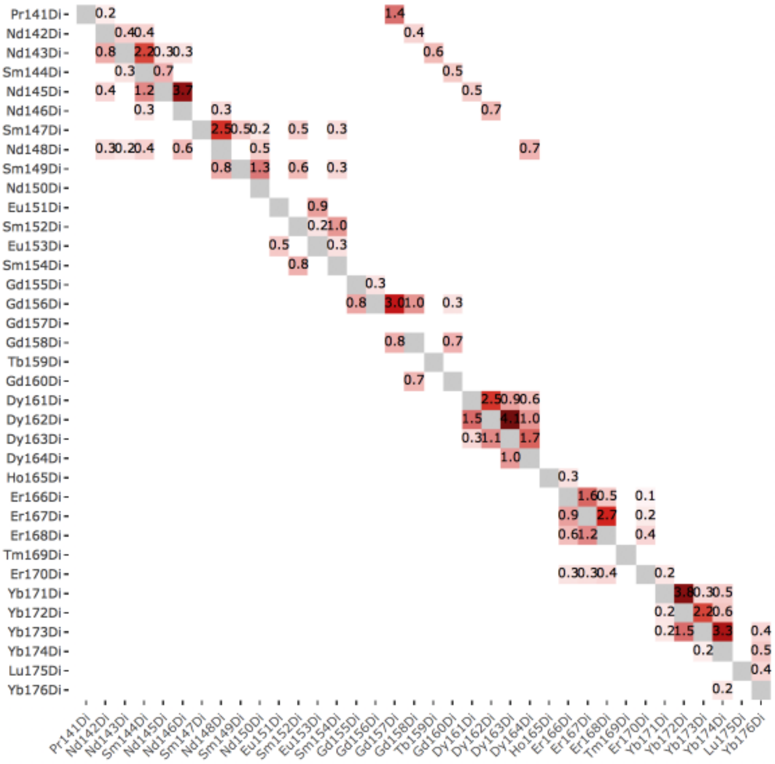

Supplementary Figure S13

Supplementary Figure S13. Spillover matrices for imaging mass cytometry data.

a-b, Spillover compensation matrices derived for the T cells & Stroma panel **(a)**, and Pan-Immune panel **(b)**. Numbers in the squares indicate percentages of spillover by channels in rows into channels in columns. Axes are labelled with metal isotopes in the format (metal)(isotopic mass number)('Di'). The degree of red from light to dark reflects an increasing degree of signal spillover. Grey boxes which do not contain values represent the identity case where the isotope on each axis is identical.

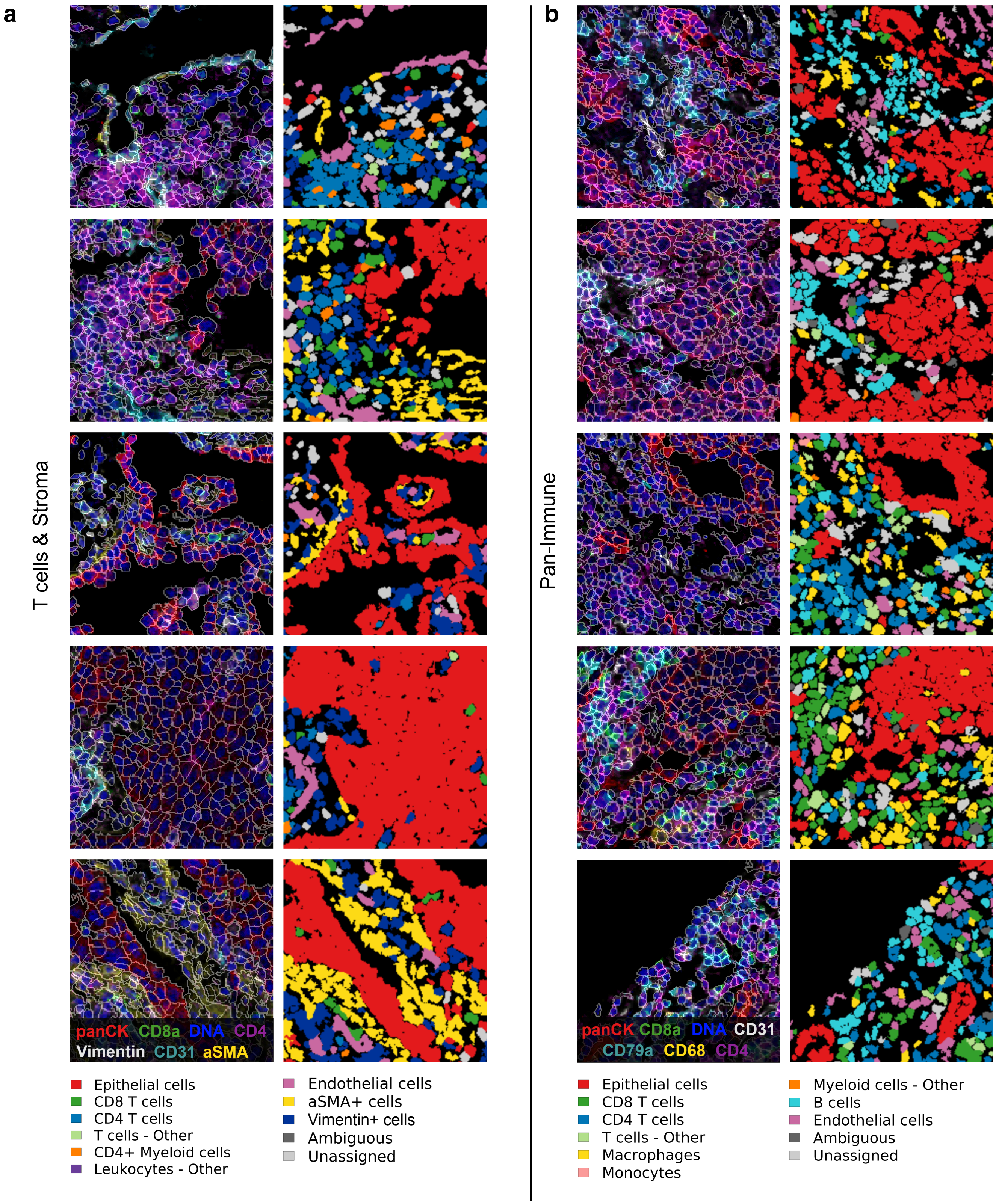

Supplementary Figure S14

Supplementary Figure S14. Multiplexed Consensus Cell Segmentation.

a, Representative overlays of the cell boundary mask produced by the Multiplexed Consensus Cell Segmentation (MCCS) segmentation method for the T cells & Stroma IMC panel (left column) with associated cell type maps showing TYPEx major cell types for the same region (right column). **b,** As for **(a)** but showing the results for the Pan-Immune panel. Colour legends for the cell boundary overlays are shown as an inset in the bottom overlay for each panel. Colour legends for the Cytomapper images are shown below images and span both columns.

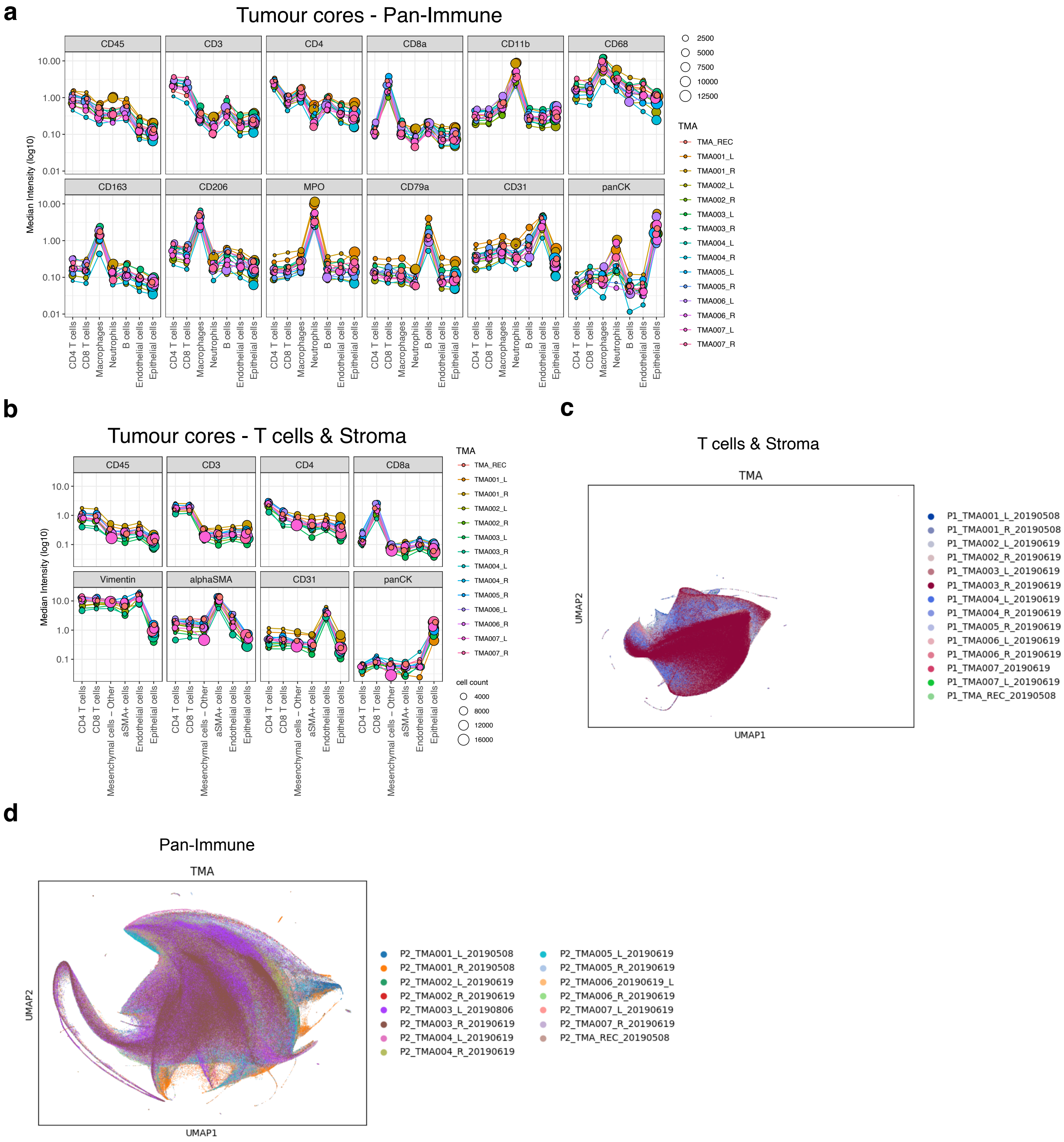

Supplementary Figure S15

Supplementary Figure S15. Investigation of batch effects.

a-b, Comparison of single cell mean intensity values of indicated cell subtypes across a selection of markers. Values were calculated for tumour cores for the Pan-Immune panel **(a)**, and tumour cores for the T cells & Stroma panel **(b)** from indicated tissue microarray (TMA) sections. Median values are represented for each stained slide. Colours are unique to each slide and the size of the circle indicates the median cell count of the indicated cell subtype per slide. **c-d,** UMAPs of raw single cell mean intensities of all markers for the T cells & Stroma **(c)** and Pan-Immune **(d)** IMC panels.

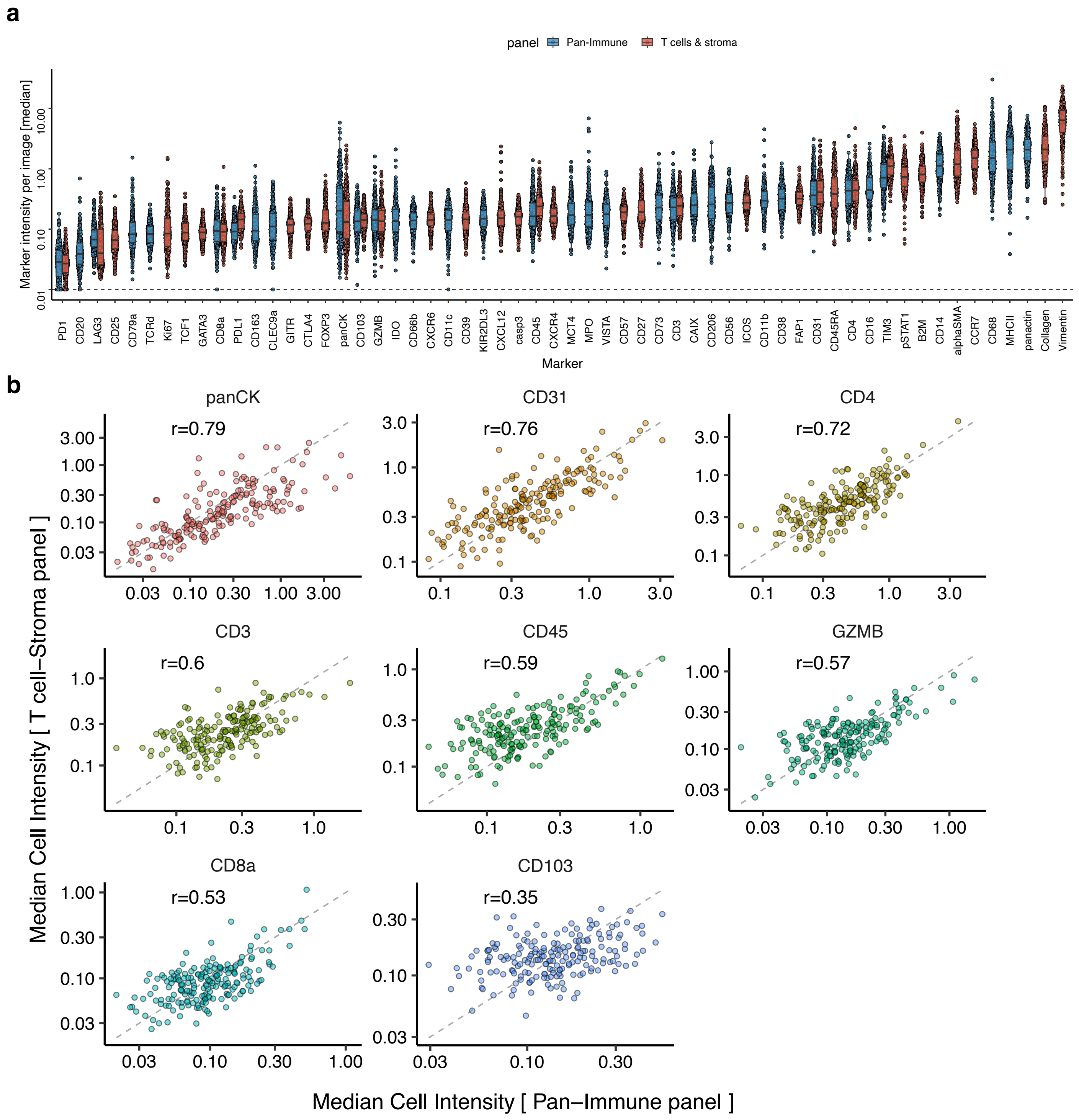

Supplementary Figure S16

Supplementary Figure S16. Raw pixel intensities.

a, Median raw pixel intensity of all cells per image for all markers included in the Pan-Immune and T cells & Stroma panels. Each point represents one image. **b,** Correlation of the median raw pixel intensity for shared cell type-defining markers across all cells per image between Pan-Immune and T cells & Stroma panels (r, Spearman correlation coefficient).

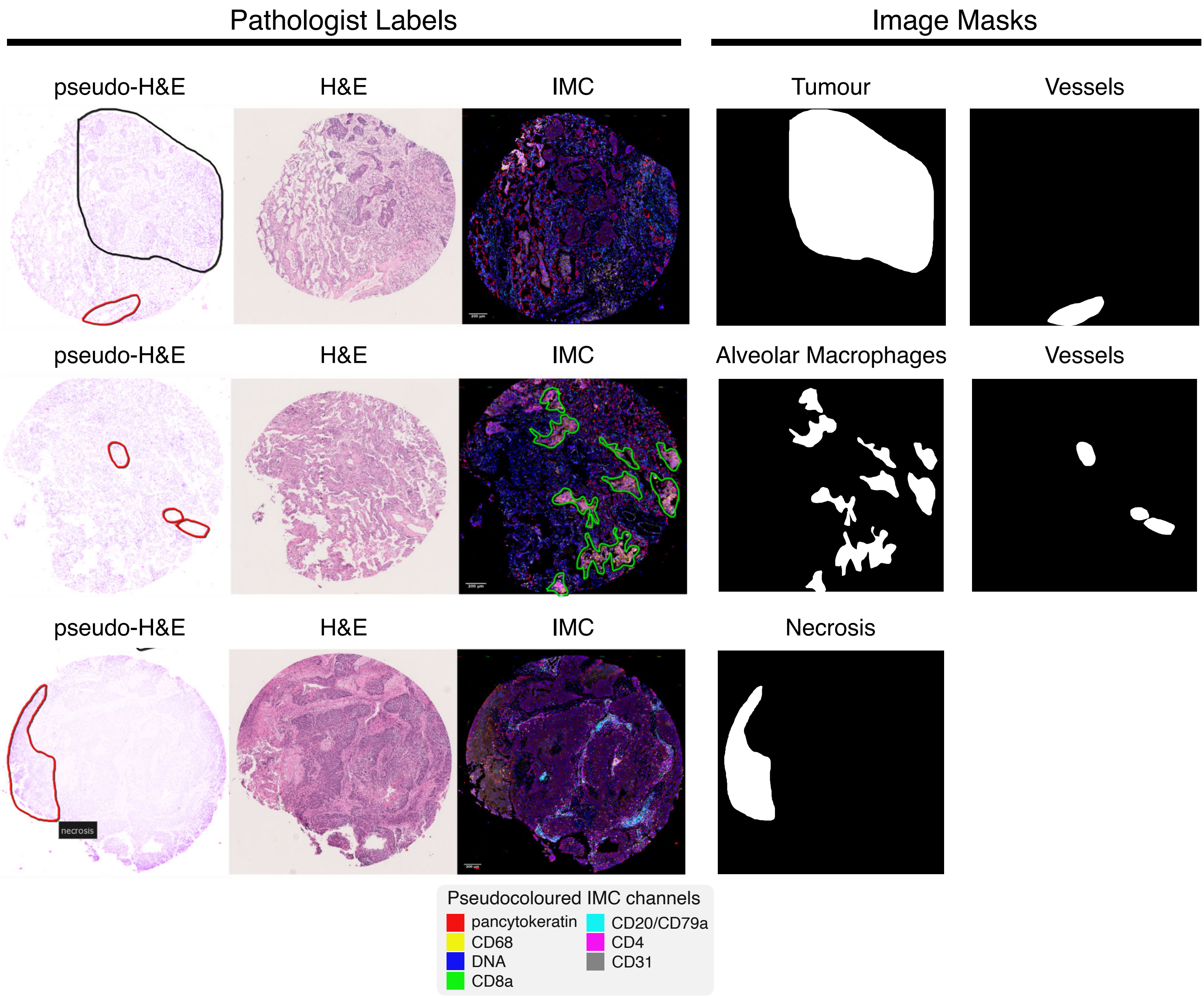

Supplementary Figure S17

Supplementary Figure S17. Pathologist-guided labels.

Pathologist manual annotations of assembled IMC-derived pseudo-H&Es generated from the iridium 191/193 and ruthenium channels, paired H&E images, and IMC pseudocoloured composites. Three tumour cores are shown with resulting image masks generated using QuPath for features included in this study. Scale bar in centre column images=200μm.

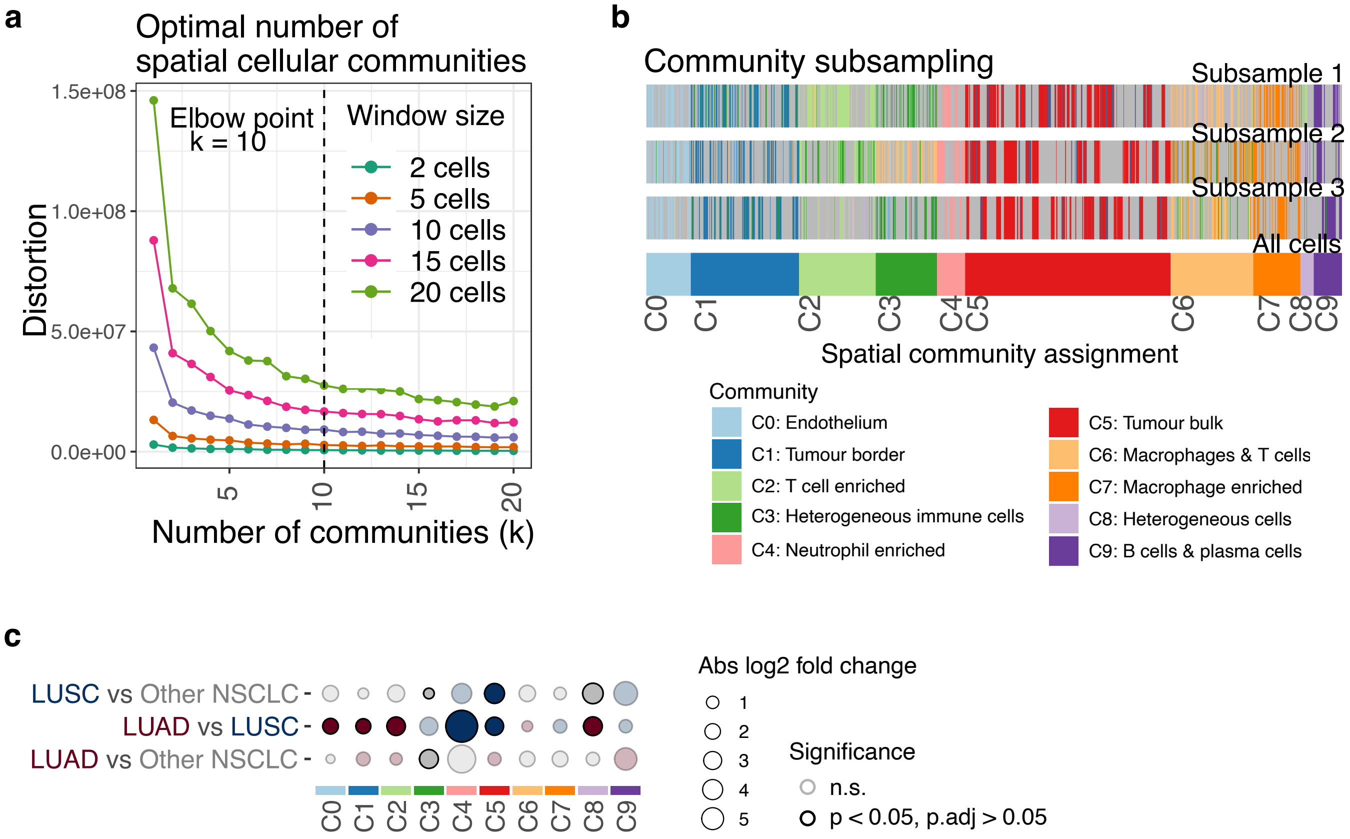

Supplementary Figure S18

Supplementary Figure S18. Communities methodology and histology associations.

a, Elbow plot showing the distortion score when clustering windows into 1 to 20 communities, across window sizes of 2, 5, 10, 15 and 20 cells. The optimal number of communities identified to maximise the decrease in dispersion score was 10 communities for all window sizes. **b,** Community assignment of each cell within a subsample compared to the full dataset. Median concordance of assignment was 80%. **c,** Association between spatial community densities and tumour histology. P-values derived from linear mixed effects models with patient as a random covariate. LUAD, lung adenocarcinoma; LUSC, lung squamous cell carcinoma; NSCLC, non-small cell lung cancer.

TIM3+ macrophages

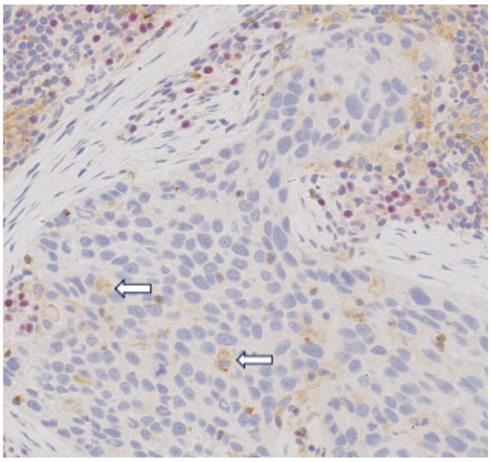

PD-L1+ macrophages

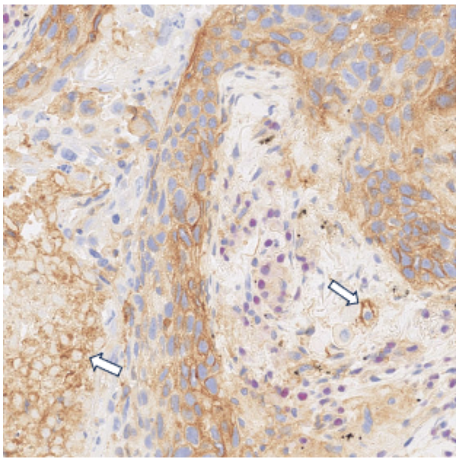

TIM3+ lymphocytes

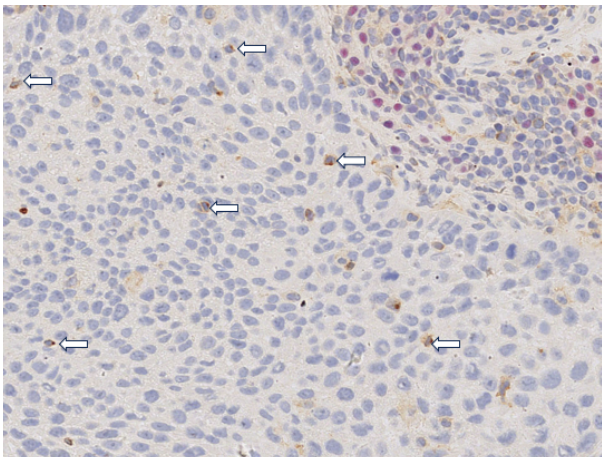

VISTA+ plasma cells

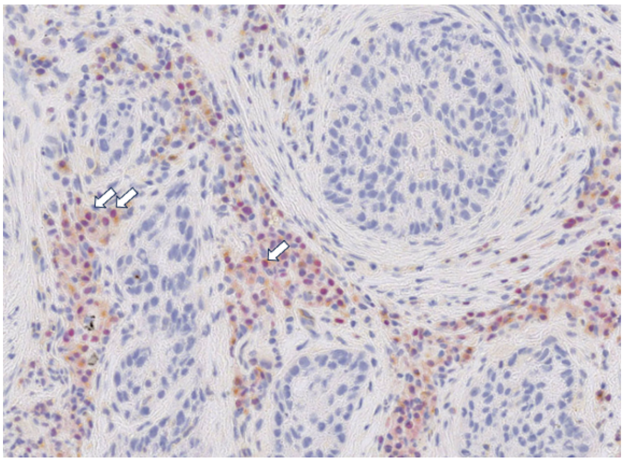

Supplementary Figure S19

Supplementary Figure S19. Multiplexed immunohistochemistry validation of checkpoint molecule expression.

Tissue sections from CRUK0083 Region 3 were co-stained with anti-MUM-1/IRF-4 (nuclear, red), one of anti-TIM3, PD-L1, or VISTA (brown) and counterstained with haematoxylin. Staining validated observations from imaging mass cytometry, including TIM3 and PD-L1 expression on macrophages, PD-L1 expression on tumour cells, and TIM3 expression on lymphocytes; co-staining of MUM-1/IRF-4 and VISTA validated frequent VISTA expression observed on plasma cells. Examples are indicated with white arrows. Images were acquired at 40x magnification.

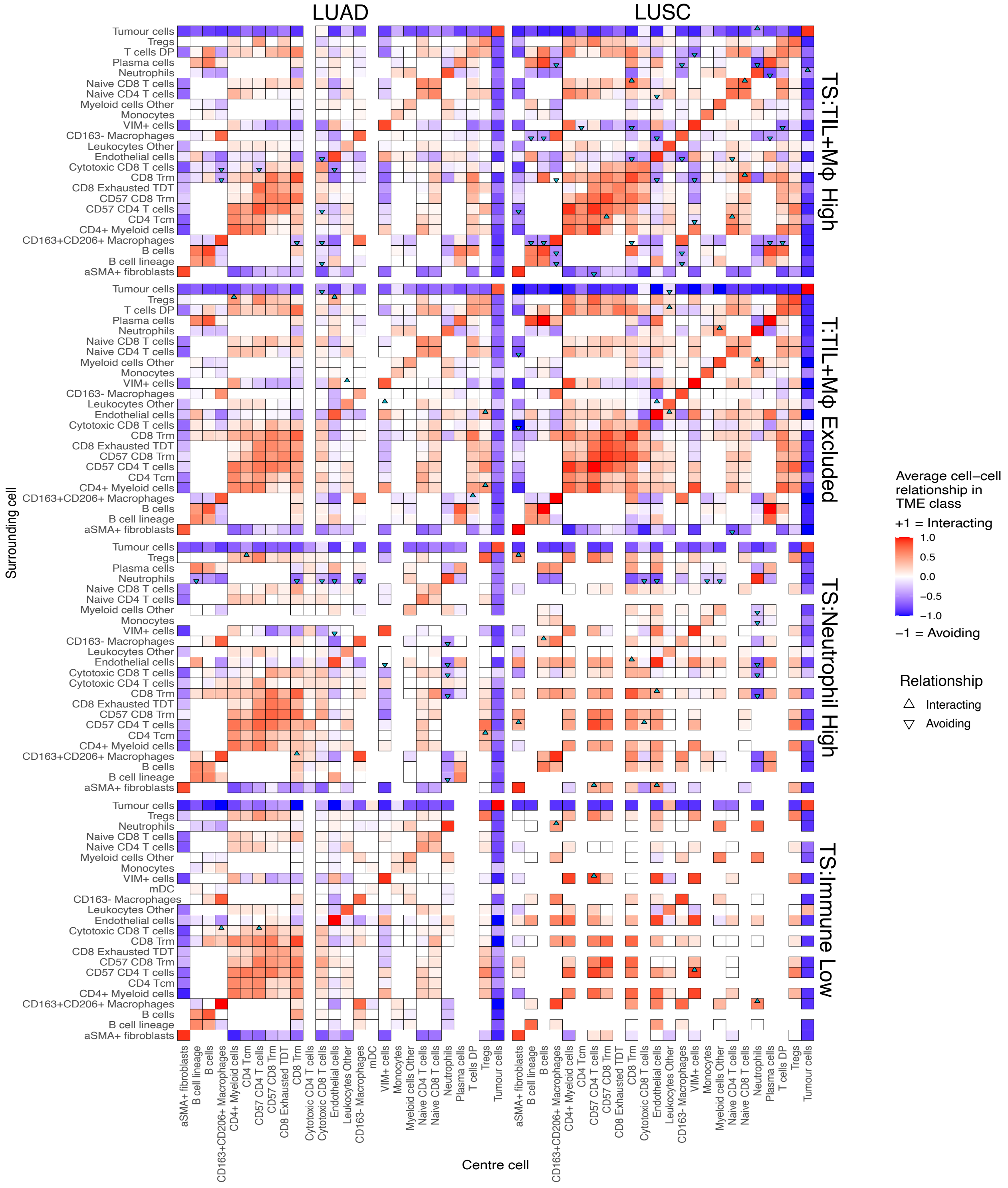

Supplementary Figure S20

Supplementary Figure S20. Cell-cell relationships differ by TME class.

Heatmaps coloured by the proportion of cores with a significant cell-cell relationship by TME class in LUAD (left) and LUSC (right). Cell-to-cell relationships were determined using neighbourhood permutation analysis per tumour core as interactions (+1,▲) or avoidances (-1,▼). The overall cell-cell relationship was summarised for all tumour cores within each subgroup. Using a logistic model, we tested if the frequency of a cell-cell relationship was significantly greater in one subgroup compared to the other. Logistic regression model accounting for multiple cores per tumour, p-values unadjusted. Significantly enriched cell-cell relationships were represented with arrows if they were present in at least 30% of tumour cores, and if the constituent cells of the relationship were present in at least 90% of tumour cores. The direction of the arrow represents the nature of the relationship: interacting (up arrow) or avoiding (down arrow). LUAD: *TS:TIL+MΦ High* vs Other (n=21 cores, 17 tumours vs n=59 cores, 30 tumours), *T:TIL+MΦ Excluded* vs Other (n=20 cores, 15 tumours vs n=60 cores, 37 tumours), *TS:Immune Low* vs Other (n=13 cores, 11 tumours vs n=67 cores, 37 tumours), *TS:Neutrophil High* vs Other (n=11 cores, 6 tumours vs n=69 cores, 37 tumours); LUSC: *TS:TIL+MΦ High* vs Other (n=13 cores, 8 tumours vs n=41 cores, 23 tumours), *T:TIL+MΦ Excluded* vs Other (n=12 cores, 8 tumours vs n=42 cores, 21 tumours), *TS:Immune Low* vs Other (n=8 cores, 4 tumours vs n=46 cores, 22 tumours), *TS:Neutrophil High* vs Other (n=15 cores, 11 tumours vs n=39 cores, 18 tumours). LUAD, lung adenocarcinoma; LUSC, lung squamous cell carcinoma; TS, Tumour/Stroma; T, Tumour; TIL, tumour-infiltrating lymphocytes; MΦ, macrophage; DP, double positive ($CD4^{+}CD8a^{+}$); TDT, terminally differentiated T cell; Trm, resident memory T cell; VIM, vimentin.

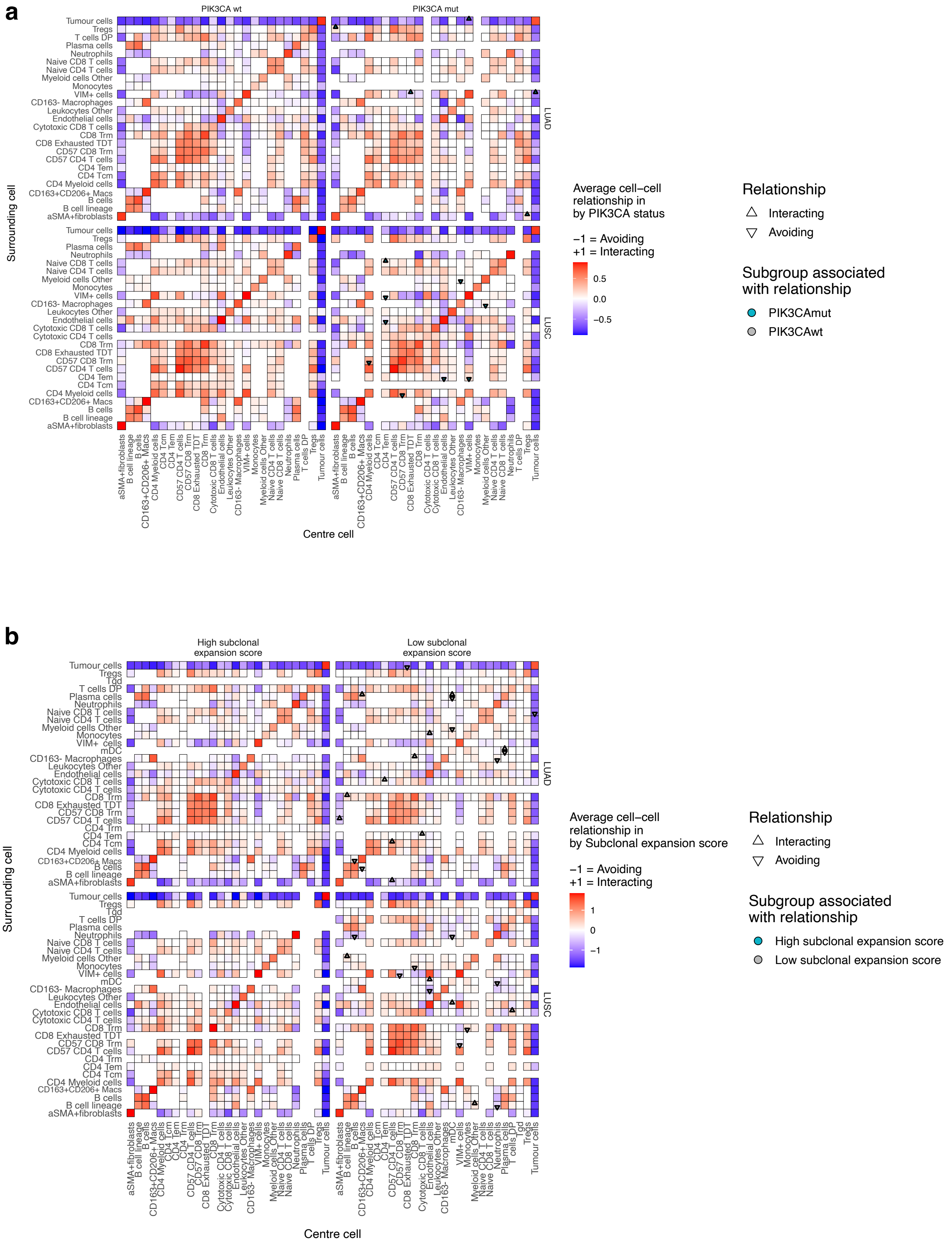

Supplementary Figure S21

Supplementary Figure S21. Cell-cell relationships differ by tumour genomics.

a-b, Heatmaps of the proportion of cores with a significant cell-cell relationship for *PIK3CA* mutation (mt) versus wild type (wt) **(a)**, and tumour cores with high compared to low subclonal expansion score stratified by the histology-specific median subclonal expansion score **(b)**, in LUAD and LUSC. neighbouRhood permutation testing accounting for cell abundance was performed to identify the overall nature of the cell-cell relationship per imaged core: interacting (+1) or avoiding (-1). The overall cell-cell relationship was summarised for all tumour cores within each subgroup. Using a logistic model, we tested if the frequency of a cell-cell relationship was significantly greater in one subgroup compared to the other. Logistic regression model accounting for multiple cores per tumour, p-values unadjusted. Significantly enriched cell-cell relationships were represented with arrows if they were present in at least 30% of tumour cores, and if the constituent cells of the relationship were present in at least 90% of tumour cores. The direction of the arrow represents the nature of the relationship: interacting (▲) or avoiding (▼). LUAD: *PIK3CA* mut vs wt (n=8 cores, 4 tumours vs n=71 cores, 36 tumours); LUSC: *PIK3CA* mut vs wt (n=16 cores, 7 tumours vs n=36 cores, 16 tumours). LUAD: subclonal expansion score high vs low (n=36 cores, 23 tumours vs n=36 cores, 23 tumours); LUSC: subclonal expansion score high vs low (n=24 cores, 15 tumours vs n=24 cores, 13 tumours). LUAD, lung adenocarcinoma; LUSC, lung squamous cell carcinoma; TS, Tumour/Stroma; T, Tumour; TIL, tumour-infiltrating lymphocytes; MΦ, macrophage; DP, double positive ($CD4^{+}CD8a^{+}$); TDT, terminally differentiated T cell; Trm, resident memory T cell; VIM, vimentin.

a

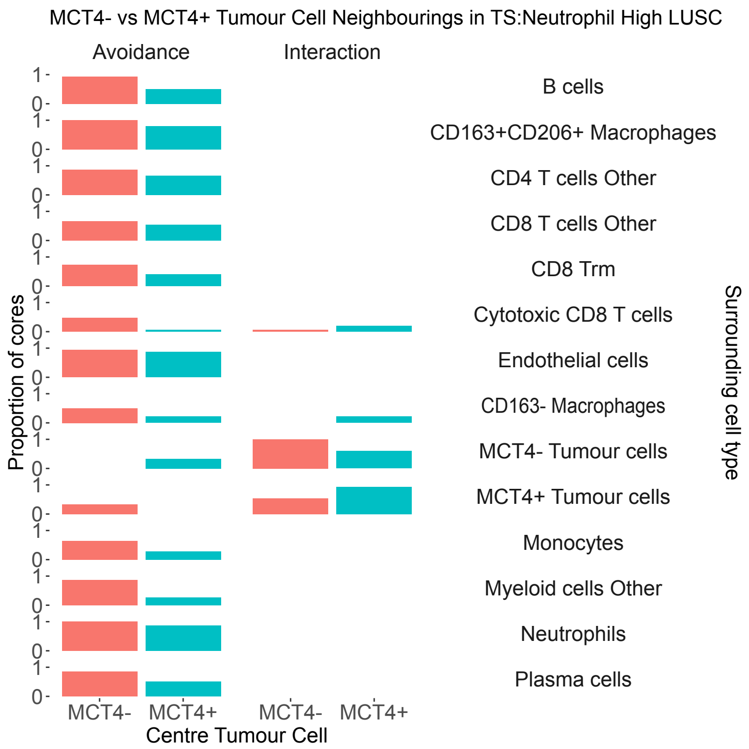

b

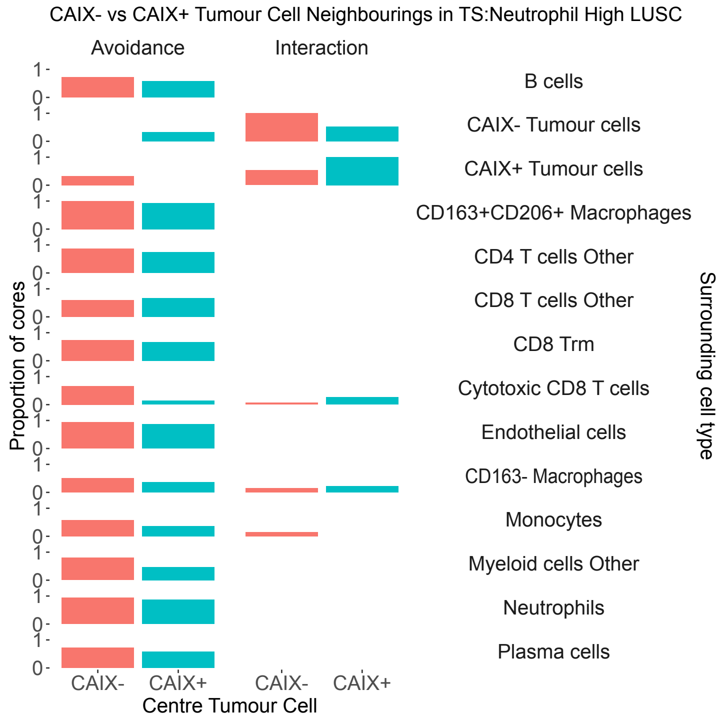

Supplementary Figure S22

Supplementary Figure S22. Cell-cell relationships based on tumour cell phenotypes.

a-b, Proportion of *TS:Neutrophil High* LUSC cores (n=15) for which each surrounding cell subtype (y-axis) exhibits a significant avoidance or interaction with the centre tumour cell subtype (x-axis) of the phenotype for MCT4 **(a)** and CAIX **(b)**. Analyses were performed using the neighbouRhood permutation approach. No significant differences were observed between positive and negative centre tumour cell phenotypes when applying a chi-square test and p-value adjustment (Benjamini-Hochberg) across all tests. Only cell subtypes for which at least two cores exhibited a significant avoidance or interaction for at least one of the two tumour phenotypes were tested, and for which ≥14 cores contained the positive tumour cell phenotype and ≥14 cores contained the negative tumour cell phenotype. LUAD, lung adenocarcinoma; LUSC, lung squamous cell carcinoma; TS, Tumour/Stroma; Trm, resident memory T cell.
