# Supplementary material for: Spatial Architecture of Myeloid and T Cells Orchestrates Immune Evasion and Clinical Outcome in Lung Cancer
Source: Cancer Discov. 2024 Apr 12;14(6):1018–47. doi: 10.1158/2159-8290.CD-23-1380 (PMC11145179; doi:10.1158/2159-8290.CD-23-1380)
Supplement: TRACERx Consortium Members — All consortium members and their affiliations. [file cd-23-1380_tracerx_consortium_members_suppsd1.docx]

TRACERx consortium

Charles Swanton^1,2,13^, Mariam Jamal-Hanjani^2,5,13^, Katey S. S. Enfield^1^, Emma Colliver^1^, Claudia Lee^1^, Alastair Magness^1^, David A. Moore^1,2,3^, Monica Sivakumar^2^, Kristiana Grigoriadis^1,2,4^, Oriol Pich^1^, Takahiro Karasaki^1,2,5^, Philip S. Hobson^6^, Selvaraju Veeriah^2^, Clare Puttick^1,2,4^, Emma L. Nye^7^, Teresa Marafioti^3^, Roberto Salgado^10,11^, Allan Hackshaw^12^, Nicholas McGranahan^2,4^, James L. Reading^2,19,20^, Sergio A. Quezada^2,20^, Crispin T. Hiley^1,2^, Mihaela Angelova^1^, Alexander M. Frankell^1,2^, Andrew Rowan^1^, Ariana Huebner^1,2,4^, Chris Bailey^1^, Cian Murphy^1^, Dhruva Biswas^1,2,24^, Emilia L. Lim^1,2^, Gareth A. Wilson^1^, Maise Al Bakir ^1,2^, Maria Zagorulya^1^, Mark S. Hill^1^, Michelle M. Leung^1,4,29^, Nicolai J Birkbak^1,2,104,106,107^, Olivia Lucas^1,2,32,100^, Rachel Rosenthal^1^, Roberto Vendramin^1,2,55^, Ryan J. Turner^1^, Sian Harries^1,2,23^, Sophia Ward^1,2,98^, Abigail Bunkum^2,5,32^, Antonia Toncheva^2^, Carlos Martínez-Ruiz^2,4^, Christopher Abbosh^2^, Corentin Richard^2^, Cristina Naceur-Lombardelli^2^, Francisco Gimeno-Valiente^2^, James R.M. Black^2,4^, Kerstin Thol^2,4^, Kevin Litchfield^2,55^, Krupa Thakkar^2^, Mariana Werner Sunderland^2^, Martin D. Forster^2,13^, Nnennaya Kanu^2^, Paulina Prymas^2^, Robert Bentham^2,4^, Sadegh Saghafinia^2^, Sharon Vanloo^2^, Simone Zaccaria^2,32^, Siow Ming Lee^2,13^, Sonya Hessey^2,5,32^, Wing Kin Liu^2,5^, Elaine Borg^3^, Mary Falzon^3^, Reena Khiroya^3^, Thomas Patrick Jones^4^, Richard Kevin Stone^7^, Abigail Sharp^12^, Anne-Marie Hacker^12^, Camilla Pilotti^12^, Harjot Kaur Dhanda^12^, Rachel Leslie^12^, Sean Smith^12^, Dionysis Papadatos-Pastos^13^, James Wilson^13^, Sarah Benafif^13,54^, Tanya Ahmad^13^, Eric Lim^22,50^, Jerome Nicod^23^, Thomas B.K. Watkins^25^, Carla Castignani^27,48^, Elizabeth Larose Cadieux^27,48^, Peter Van Loo^27,121,122^, Karl S. Peggs^28,33^, Khalid AbdulJabbar^30^, Catarina Veiga^31^, George Kassiotis^34,52^, Gary Royle^36^, Charles-Antoine Collins-Fekete^37^, Alexander James Procter^38^, Arjun Nair^38,56^, Asia Ahmed^38^, Magali N. Taylor^38^, Neal Navani ^39,47^, Ricky M. Thakrar^39,47^, David Lawrence^40^, Davide Patrini^40^, Akshay J. Patel^41^, David Chuter^42^, Mairead MacKenzie^42^, Francesco Fraioli^43^, Zoltan Kaplar^44,115^, Paul Ashford^45^, Sam M. Janes^46^, Miljana Tanic^48,116^, Stephan Beck^48^, Andrew G. Nicholson^49,50^, Alexandra Rice^50^, Anand Devaraj^50^, Chiara Proli^50^, Daniel Kaniu^50^, Harshil Bhayani^50^, Hemangi Chavan^50^, Hilgardt Raubenheimer^50^, Lyn Ambrose^50^, Mpho Malima^50^, Nadia Fernandes^50^, Paulo De Sousa^50^, Pratibha Shah^50^, Sarah Booth^50^, Silviu I Buderi^50^, Simon Jordan^50^, Sofina Begum^50^, Ekaterini Boleti^51^, Clare E. Weeden^52^, Eva Grönroos^52^, Imran Noorani^52^, Jacki Goldman^52^, Mickael Escudero^52^, Stefan Boeing^52^, Tamara Denner^52^, Vittorio Barbè^52^, Wei-Ting Lu^52^, William Hill^52^, Yutaka Naito^52^, Zoe Ramsden^52^, Anca Grapa^53^, Hanyun Zhang^53^, Jack French^54^, Kayleigh Gilbert^54^, Angela Dwornik^57^, Angeliki Karamani^57^, Benny Chain^57^, David R Pearce^57^, Despoina Karagianni ^57^, Felip Gálvez-Cancino ^57^, Georgia Stavrou^57^, Gerasimos-Theodoros Mastrokalos^57^, Helen L. Lowe^57^, Ignacio Garcia Matos^57^, John A. Hartley^57^, Kayalvizhi Selvaraju^57^, Kezhong Chen^57^, Leah Ensell^57^, Mansi Shah^57^, Maria Litovchenko^57^, Olga Chervova^57,59^, Othman Al-Sawaf^57,111^, Piotr Pawlik^57^, Robert E. Hynds^57^, Samuel Gamble^57^, Seng Kuong Anakin Ung^57^, Supreet Kaur Bola^57^, Victoria Spanswick^57^, Yin Wu^57^, Jayant K. Rane^58^, Emilie Martinoni Hoogenboom ^60^, Fleur Monk^60^, James W. Holding^60^, Junaid Choudhary^60^, Kunal Bhakhri^60^, Marco Scarci^60^, Pat Gorman^60^, Robert CM Stephens^60^, Steve Bandula^60^, Yien Ning Sophia Wong^60^, Heather Cheyne^61^, Mohammed Khalil^61^, Shirley Richardson ^61^, Tracey Cruickshank^61^, Gillian Price^62,64^, Keith M. Kerr^63,64^, Babu Naidu^65^, Gary Middleton^66,67^, Aya Osman^67^, Carol Enstone^67^, Gerald Langman^67^, Helen Shackleford^67^, Madava Djearaman^67^, Salma Kadiri^67^, Alan Kirk^68^, Mathew Thomas^68^, Mo Asif^68^, Nikos Kostoulas^68^, Rocco Bilancia^68^, Kevin G. Blyth^69,75,76^, John Le Quesne^70,71,73^, Andrew Kidd^72^, Craig Dick^74^, Madeleine Hewish^77,78^, Lily Robinson^79^, Peter Russell^79^, Gurdeep Matharu^80^, Jacqui A. Shaw^80^, Claire Wilson^81^, Amrita Bajaj^82^, Apostolos Nakas^82^, Azmina Sodha-Ramdeen^82^, Dean A. Fennell^82,83^, Mohamad Tufail^82^, Molly Scotland^82^, Rebecca Boyles^82^, Sean Dulloo^82,83^, Sridhar Rathinam^82^, Domenic Marrone^83^, Michael J. Shackcloth^84^, Caroline Dive^85,86^, Jonathan Tugwood^85,86^, Anshuman Chaturvedi^87,91^, Colin R. Lindsay^87,88^, Fiona H. Blackhall^87,88^, Katherine D. Brown^87,91^, Mathew Carter^87,91^, Pedro Oliveira^87,91^, Philip Crosbie^87,89,97^, Yvonne Summers^87,88^, Matthew G. Krebs^88^, Antonio Paiva-Correia^90^, Sarah Danson^92,93^, Judith Cave^94^, Aiman Alzetani^95^, Jennifer Richards^95^, Serena Chee^95^, Jason F. Lester^96^, Elaine Smith^97^, Eustace Fontaine^97^, Felice Granato^97^, Juliette Novasio^97^, Kendadai Rammohan^97^, Leena Joseph^97^, Paul Bishop^97^, Rajesh Shah^97^, Stuart Moss^97^, Vijay Joshi^97^, Angela Leek^99^, Jack Davies Hodgkinson^99^, Nicola Totton^99^, Hugo J.W.L. Aerts^117,118,120^, Tom L. Kaufmann^108,109^, Roland F. Schwarz^109,112^, Judit Kisistok^104,106,107^, Mateo Sokac^104,106,107^, Matthew R. Huska^110^, Zoltan Szallasi^105,113,119^, Miklos Diossy^105,114,119^, Jonas Demeulemeester^101,102,103^, Xiaoxi Pan^123^, Yinyin Yuan^123^

Affiliations

^1^Cancer Evolution and Genome Instability Laboratory, The Francis Crick Institute, London, UK.

^2^Cancer Research UK Lung Cancer Centre of Excellence, University College London Cancer Institute, London, UK.

^3^Department of Cellular Pathology, University College London Hospitals, London, UK.

^4^Cancer Genome Evolution Research Group, Cancer Research UK Lung Cancer Centre of Excellence, University College London Cancer Institute, London, UK.

^5^Cancer Metastasis Laboratory, University College London Cancer Institute, London, UK.

^7^Experimental Histopathology, The Francis Crick Institute, London, UK.

^10^Department of Pathology, ZAS Hospitals, Antwerp, Belgium.

^11^Division of Research, Peter MacCallum Cancer Centre, Melbourne, Australia.

^12^Cancer Research UK & University College London Cancer Trials Centre, London, UK.

^13^Department of Oncology, University College London Hospitals, London, UK.

^19^Pre-cancer Immunology Laboratory, University College London Cancer Institute, London, UK.

^20^Immune Regulation and Tumour Immunotherapy Group, Cancer Immunology Unit, Research Department of Haematology, University College London Cancer Institute, London, UK.

^22^Academic Division of Thoracic Surgery, Imperial College London, London, UK.

^23^Advanced Sequencing Facility, The Francis Crick Institute, London, UK.

^24^Bill Lyons Informatics Centre, University College London Cancer Institute, London, UK.

^25^Cancer Evolution and Genome Instability Laboratory, The Francis Crick Institute and University College London Cancer Institute, London, UK.

^26^Cancer Genome Evolution Research Group, University College London Cancer Institute, London, UK.

^27^Cancer Genomics Laboratory, The Francis Crick Institute, London, UK.

^28^Cancer Immunology Unit, Research Department of Haematology, University College London Cancer Institute, London, UK.

^29^Cancer Research UK Lung Cancer Centre of Excellence, University College London, Cancer Institute, London, UK.

^30^Case^45^, London, UK.

^31^Centre for Medical Image Computing, Department of Medical Physics and Biomedical Engineering, London, UK.

^32^Computational Cancer Genomics Research Group, University College London Cancer Institute, London, UK.

^33^Department of Haematology, University College London Hospitals, London, UK.

^34^Department of Infectious Disease, Faculty of Medicine, Imperial College London, London, UK.

^35^Department of Medical Oncology, University College London Hospitals, London, UK.

^36^Department of Medical Physics and Bioengineering, University College London Cancer Institute, London, UK.

^37^Department of Medical Physics and Biomedical Engineering, University College London, London, UK.

^38^Department of Radiology, University College London Hospitals, London, UK.

^39^Department of Thoracic Medicine, University College London Hospitals, London, UK.

^40^Department of Thoracic Surgery, University College London Hospital NHS Trust, London, UK.

^41^Guy’s and St Thomas’ NHS Foundation Trust, London, UK.

^42^Independent Cancer Patient's voice, London, UK.

^43^Institute of Nuclear Medicine, Division of Medicine, University College London, London, UK.

^44^Institute of Nuclear Medicine, University College London Hospitals, London, UK.

^45^Institute of Structural and Molecular Biology, University College London, London, UK.

^46^Lungs for Living Research Centre, UCL Respiratory, Department of Medicine, University College London, London, UK.

^47^Lungs for Living Research Centre, UCL Respiratory, University College London, London, UK.

^48^Medical Genomics, University College London Cancer Institute, London, UK.

^49^National Heart and Lung Institute, Imperial College London, London, UK.

^50^Royal Brompton and Harefield Hospitals, part of Guy’s and St Thomas’ NHS Foundation Trust, London, UK.

^51^Royal Free London NHS Foundation Trust, London, UK.

^52^The Francis Crick Institute, London, UK.

^53^The Institute of Cancer Research, London, UK.

^54^The Whittington Hospital NHS Trust, London, UK.

^55^Tumour Immunogenomics and Immunosurveillance Laboratory, University College London Cancer Institute, London, UK.

^56^UCL Respiratory, Department of Medicine, University College London, London, UK.

^57^University College London Cancer Institute, London, UK.

^58^University College London Cancer Institute, London, UK and Cancer Evolution and Genome Instability Laboratory, The Francis Crick Institute, London, UK.

^59^University College London Department of Epidemiology and Health Care, London, UK.

^60^University College London Hospitals, London, UK.

^61^Aberdeen Royal Infirmary NHS Grampian, Aberdeen, UK.

^62^Department of Medical Oncology, Aberdeen Royal Infirmary NHS Grampian, Aberdeen, UK.

^63^Department of Pathology, Aberdeen Royal Infirmary NHS Grampian, Aberdeen, UK.

^64^University of Aberdeen, Aberdeen, UK.

^65^Birmingham Acute Care Research Group, Institute of Inflammation and Ageing, University of Birmingham, Birmingham, UK.

^66^Institute of Immunology and Immunotherapy, University of Birmingham, Birmingham, UK.

^67^University Hospital Birmingham NHS Foundation Trust, Birmingham, UK.

^68^Golden Jubilee National Hospital, Clydebank, UK.

^69^Beatson Institute for Cancer Research, University of Glasgow, Glasgow, UK.

^70^Cancer Research UK Scotland Institute, Glasgow, UK.

^71^Institute of Cancer Sciences, University of Glasgow, Glasgow, UK.

^72^Institute of Infection, Immunity & Inflammation, University of Glasgow, Glasgow, UK.

^73^NHS Greater Glasgow and Clyde Pathology Department, Queen Elizabeth University Hospital, Glasgow, UK.

^74^NHS Greater Glasgow and Clyde, Glasgow, UK.

^75^Queen Elizabeth University Hospital, Glasgow, UK.

^76^School of Cancer Sciences, University of Glasgow, Glasgow, UK.

^77^Royal Surrey Hospital, Royal Surrey Hospitals NHS Foundation Trust, Guildford, UK.

^78^University of Surrey, Guildford, UK.

^79^Princess Alexandra Hospital, The Princess Alexandra Hospital NHS Trust, Harlow, UK.

^80^Cancer Research Centre, University of Leicester, Leicester, UK.

^81^Leicester Medical School, University of Leicester, Leicester, UK.

^82^University Hospitals of Leicester NHS Trust, Leicester, UK.

^83^University of Leicester, Leicester, UK.

^84^Liverpool Heart and Chest Hospital, Liverpool, UK.

^85^CRUK Lung Cancer Centre of Excellence, University of Manchester, Manchester, UK.

^86^CRUK Manchester Institute Cancer Biomarker Centre, University of Manchester, Manchester, UK.

^87^Cancer Research UK Lung Cancer Centre of Excellence, University of Manchester, Manchester, UK.

^88^Division of Cancer Sciences, The University of Manchester and The Christie NHS Foundation Trust, Manchester, UK.

^89^Division of Infection, Immunity and Respiratory Medicine, University of Manchester, Manchester, UK.

^90^Manchester University NHS Foundation Trust, Manchester, UK.

^91^The Christie NHS Foundation Trust, Manchester, UK.

^92^Sheffield Teaching Hospitals NHS Foundation Trust, Sheffield, UK.

^93^University of Sheffield, Sheffield, UK.

^94^Department of Oncology, University Hospital Southampton NHS Foundation Trust, Southampton, UK.

^95^University Hospital Southampton NHS Foundation Trust, Southampton, UK.

^96^Singleton Hospital, Swansea Bay University Health Board, Swansea, UK.

^97^Wythenshawe Hospital, Manchester University NHS Foundation Trust, Wythenshawe, UK.

^98^Advanced Sequencing Facility, The Francis Crick Institute, London, UK .

^99^Manchester Cancer Research Centre Biobank, Manchester, UK .

^100^University College London Hospitals, London, UK .

^101^Department of Oncology, KU Leuven, Leuven, Belgium.

^102^Integrative Cancer Genomics Laboratory, VIB Center for Cancer Biology, Leuven, Belgium.

^103^VIB Center for AI & Computational Biology, Belgium.

^104^Bioinformatics Research Centre, Aarhus University, Aarhus, Denmark.

^105^Danish Cancer Society Research Center, Copenhagen, Denmark.

^106^Department of Clinical Medicine, Aarhus University, Aarhus, Denmark.

^107^Department of Molecular Medicine, Aarhus University Hospital, Aarhus, Denmark.

^108^Berlin Institute for Medical Systems Biology, Max Delbrück Center for Molecular Medicine in the Helmholtz Association (MDC), Berlin, Germany.

^109^Berlin Institute for the Foundations of Learning and Data (BIFOLD), Berlin, Germany.

^110^Bioinformatics and Systems Biology, Method Development and Research Infrastructure, Robert Koch Institute, Berlin, Germany.

^111^Department I of Internal Medicine, University Hospital of Cologne, Cologne, Germany.

^112^Institute for Computational Cancer Biology, Center for Integrated Oncology (CIO), Cancer Research Center Cologne Essen (CCCE), Faculty of Medicine and University Hospital Cologne, University of Cologne, Germany.

^113^Department of Bioinformatics, Semmelweis University, Budapest, Hungary.

^114^Department of Physics of Complex Systems, ELTE Eötvös Loránd University, Budapest, Hungary.

^115^Integrated Radiology Department, North-buda St. John's Central Hospital, Budapest, Hungary.

^116^Experimental Oncology, Institute for Oncology and Radiology of Serbia, Belgrade, Serbia.

^117^Radiology and Nuclear Medicine, CARIM & GROW, Maastricht University, Maastricht, The Netherlands.

^118^Artificial Intelligence in Medicine (AIM) Program, Mass General Brigham, Harvard Medical School, Boston, MA, USA.

^119^Computational Health Informatics Program, Boston Children's Hospital, Boston, MA, USA.

^120^Department of Radiation Oncology, Brigham and Women’s Hospital, Dana-Farber Cancer Institute, Harvard Medical School, Boston, MA, USA.

^121^Department of Genetics, The University of Texas MD Anderson Cancer Center, Houston, TX, USA.

^122^Department of Genomic Medicine, The University of Texas MD Anderson Cancer Center, Houston, TX, USA.

^123^The University of Texas MD Anderson Cancer Center, Houston, TX, USA.
